# Slide-tags enables single-nucleus barcoding for multimodal spatial genomics

**DOI:** 10.1038/s41586-023-06837-4

**Published:** 2023-12-13

**Authors:** Andrew J. C. Russell, Jackson A. Weir, Naeem M. Nadaf, Matthew Shabet, Vipin Kumar, Sandeep Kambhampati, Ruth Raichur, Giovanni J. Marrero, Sophia Liu, Karol S. Balderrama, Charles R. Vanderburg, Vignesh Shanmugam, Luyi Tian, J. Bryan Iorgulescu, Charles H. Yoon, Catherine J. Wu, Evan Z. Macosko, Fei Chen

**Affiliations:** 1 Broad Institute of Harvard and MIT, Cambridge, MA USA; 2 Department of Stem Cell and Regenerative Biology, Harvard University, Cambridge, MA USA; 3 Biological and Biomedical Sciences Program, Harvard University, Cambridge, MA USA; 4 Department of Biomedical Informatics, Harvard University, Boston, MA USA; 5 Biophysics Program, Harvard University, Boston, MA USA; 6 Harvard-MIT Division of Health Sciences and Technology, Massachusetts Institute of Technology, Cambridge, MA USA; 7 Department of Pathology, Brigham and Women’s Hospital, Harvard Medical School, Boston, MA USA; 8 Department of Medical Oncology, Dana-Farber Cancer Institute, Boston, MA USA; 9 Department of Medicine, Brigham and Women’s Hospital, Harvard Medical School, Boston, MA USA; 10 Division of Stem Cell Transplantation and Cellular Therapies, Dana-Farber Cancer Institute, Boston, MA USA; 11 Department of Surgical Oncology, Brigham and Women’s Hospital, Harvard Medical School, Boston, MA USA; 12 Department of Psychiatry, Massachusetts General Hospital, Boston, MA USA; 13 Present Address: Guangzhou Laboratory, Guangdong, China; 14 Present Address: Molecular Diagnostics Laboratory, Department of Hematopathology, Division of Pathology and Laboratory Medicine, The University of Texas MD Anderson Cancer Center, Houston, TX USA

**Keywords:** Genomics, Gene expression analysis, Epigenetics analysis

## Abstract

Recent technological innovations have enabled the high-throughput quantification of gene expression and epigenetic regulation within individual cells, transforming our understanding of how complex tissues are constructed^1–6^. However, missing from these measurements is the ability to routinely and easily spatially localize these profiled cells. We developed a strategy, Slide-tags, in which single nuclei within an intact tissue section are tagged with spatial barcode oligonucleotides derived from DNA-barcoded beads with known positions. These tagged nuclei can then be used as an input into a wide variety of single-nucleus profiling assays. Application of Slide-tags to the mouse hippocampus positioned nuclei at less than 10 μm spatial resolution and delivered whole-transcriptome data that are indistinguishable in quality from ordinary single-nucleus RNA-sequencing data. To demonstrate that Slide-tags can be applied to a wide variety of human tissues, we performed the assay on brain, tonsil and melanoma. We revealed cell-type-specific spatially varying gene expression across cortical layers and spatially contextualized receptor–ligand interactions driving B cell maturation in lymphoid tissue. A major benefit of Slide-tags is that it is easily adaptable to almost any single-cell measurement technology. As a proof of principle, we performed multiomic measurements of open chromatin, RNA and T cell receptor (TCR) sequences in the same cells from metastatic melanoma, identifying transcription factor motifs driving cancer cell state transitions in spatially distinct microenvironments. Slide-tags offers a universal platform for importing the compendium of established single-cell measurements into the spatial genomics repertoire.

## Abstract (web summary)

Slide-tags enables multiomic sequencing of single cells and their localization within tissues.

## Main

Technology development efforts in genomics during the past decade have produced an extensive toolkit of single-cell and single-nucleus sequencing methods, enabling high-throughput molecular characterization of many macromolecules^1–6^. However, missing from these measurements is the cytoarchitectural organization of the cells being profiled. Spatially resolved sequencing technologies aim to address this drawback by barcoding macromolecules with oligonucleotides of which the spatial positions are known^7–10^. However, direct transfer of design principles from single-cell sequencing methods to spatially resolved profiling is often impossible, necessitating the reinvention of each molecular assay (such as transcriptomics^8,9^, mutations^7^ or assay for transposase-accessible chromatin with sequencing (ATAC–seq)^11–13^) in a spatial context. Furthermore, while single-cell computational tools are extremely mature^14^, additional sources of noise in spatial genomics techniques require their redesign as well, for example, to address problems with cellular mixing^15–17^. An alternative to capture-based strategies is to isolate single cells while retaining spatial barcoding information; to date, this has been demonstrated only at a limited spatial resolution and with sparse sampling of tissues^18,19^. An ideal spatial genomics technology would (1) efficiently capture cell profiles from tissue sections; (2) resolve cellular positions at low-micrometre resolutions; and (3) be generally applicable to any single-cell methodology.

Here we introduce Slide-tags, a method in which cellular nuclei from an intact fresh frozen tissue section are ‘tagged’ with spatial barcode oligonucleotides derived from DNA-barcoded beads with known positions. Isolated nuclei are then profiled using existing single-cell methods with the addition of spatial positions. We demonstrate the tissue versatility of Slide-tags by assaying adult and developing mouse brain, human cerebral cortex, human tonsil and human melanoma. Across tissues and species, we import spatially tagged nuclei into standard workflows for single-nucleus RNA-sequencing (snRNA-seq), single-nucleus ATAC–seq (snATAC–seq) and TCR sequencing. Slide-tags is also readily integrated into established single-cell computational workflows, such as copy-number variation (CNV) inference. In doing so, we take advantage of the truly single-cell, spatially resolved, multimodal capacity of Slide-tags to reveal cell-type-specific spatially varying gene expression, spatially contextualize receptor–ligand interactions and examine genetic and epigenetic factors participating in tumour microenvironments.

## Labelling nuclei with spatial barcodes

**Fig. 1:**
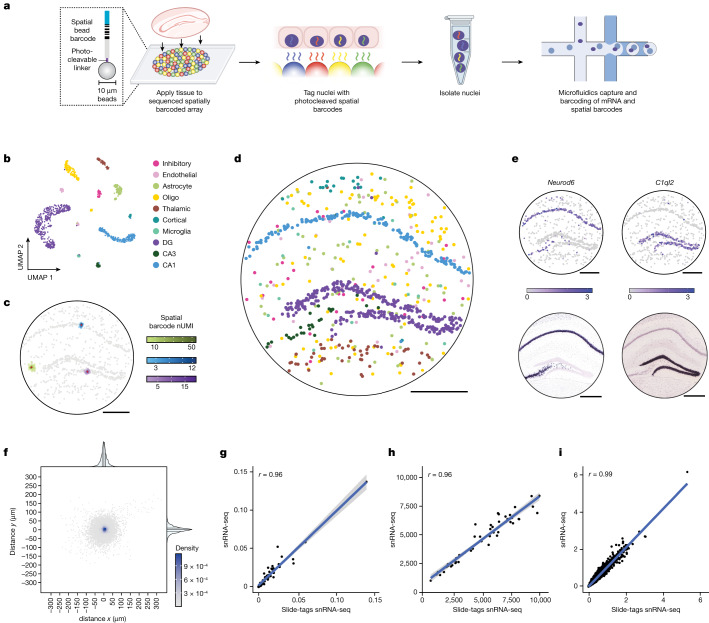
Slide-tags enables single-nucleus spatial transcriptomics in the mouse hippocampus. **a**, Schematic of Slide-tags. A 20-μm fresh-frozen tissue section is applied to a monolayer of randomly deposited, DNA-barcoded beads that have been spatially indexed. These DNA spatial barcodes are photocleaved and associate with nuclei. Spatially barcoded nuclei are then profiled using established droplet-based single-nucleus sequencing technologies. The diagram was created using BioRender. **b**, Uniform manifold approximation and projection (UMAP) embedding of snRNA-seq profiles coloured by cell type annotations. DG, dentate gyrus; oligo, oligodendrocyte. **c**, The signal spatial barcode clusters after noise filtering by DBSCAN for selected cells, coloured according to cell type annotations (as in **b**) and the number of spatial barcode UMIs. Raw plots for these cells are plotted in Extended Data Fig. 2k. **d**, Slide-tags enables localization of nuclei to spatial coordinates in the mouse hippocampus; cells are coloured according to cell type annotation (as in **b**). **e**, Spatial expression of known marker genes compared with in situ hybridization data from the Allen Mouse Brain Atlas^23^. Colour scales, normalized average counts. **f**, The distance from the centroid for each of the spatial barcodes across all signal spatial barcode clusters; points are coloured by the two-dimensional kernel density estimation with an axis-aligned bivariate normal kernel, evaluated on a square grid. Kernel density estimates are displayed for *x* and *y*. For the plots on the outside, the centre lines show the median, and the adjacent lines show the upper and lower quartiles. **g**–**i**, Comparison metrics plotted for snRNA-seq compared with Slide-tags snRNA-seq, performed on consecutive sections. **g**, Cell type proportions and mean UMIs per cell are plotted by cell type. **h**, The normalized average UMI counts were determined per gene across all cells. **i**, Normalized average counts. Expression counts for each cell were divided by the total counts for that cell and multiplied by 10,000, this value + 1 was then natural-log transformed. *r* is the Pearson correlation coefficient. The error bands denote the 95% confidence intervals. For **c**–**e**, scale bars, 500 μm.

We previously developed densely packed spatially indexed arrays of DNA-barcoded 10 μm beads, generated using split-pool phosphoramidite synthesis and indexed by sequencing-by-ligation^7,9,20^. In our original Slide-seq methodology, DNA or RNA from tissues was captured and spatially barcoded using these arrays. In our Slide-tags technology, we photocleave and diffuse these bead-derived spatial barcodes into 20 μm fresh frozen tissue sections to associate them with nuclei (Fig. 1a). We postulated that, once these barcodes are associated with nuclei, they could be used as input to established single-nucleus sequencing approaches (Methods) with only minor protocol modifications.

## Slide-tags snRNA-seq in the mouse brain

**Extended Data Fig. 1:**
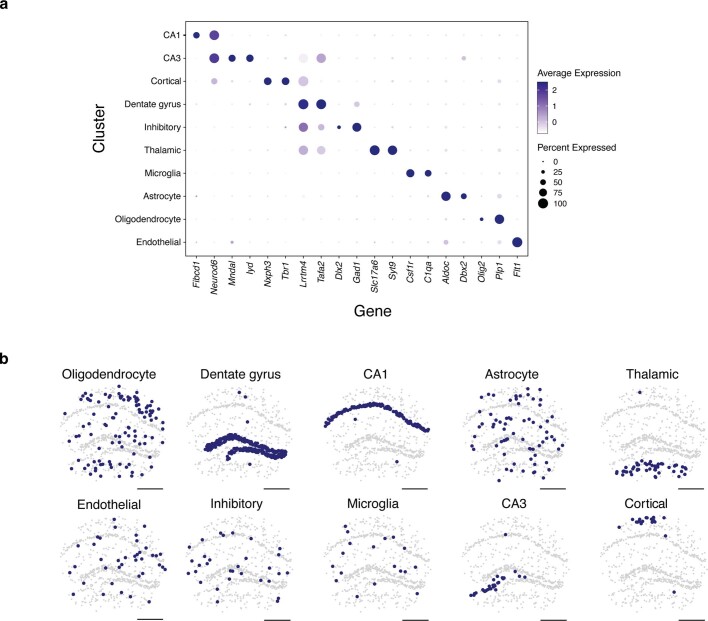
Cell type assignment and spatial mapping in the mouse hippocampus. **a**, Expression of marker genes by cell type cluster. **b**, Spatial positions of each cell by cell type cluster. All scale bars denote 500 μm. CA1 = Cornu Ammonis area 1, CA3 = Cornu Ammonis area 3. n = 839 nuclei.

**Extended Data Fig. 2:**
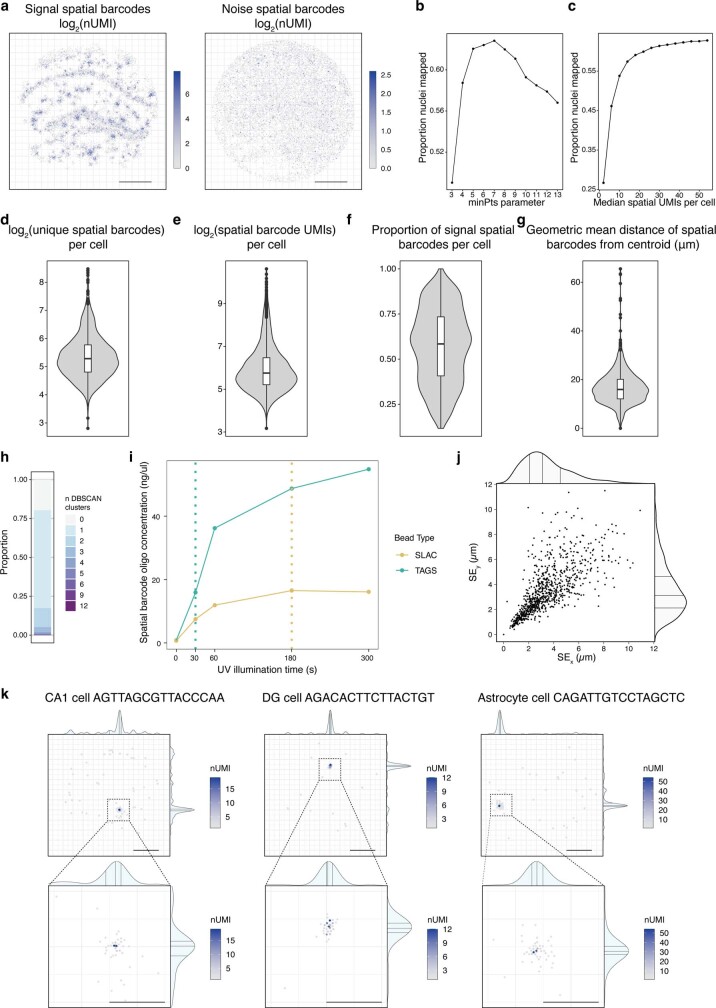
Assessing the mapping of single nuclei using spatial barcodes in the mouse hippocampus. **a**, Each recovered signal and noise spatial barcode is shown coloured by the number of detected UMIs. **b**, The proportion of nuclei mapped for each minPts parameter tested in DBSCAN. **c**, The proportion of nuclei mapped at different median spatial barcode nUMIs per cell. **d-e**, Violin plots showing different spatial barcode metrics for every cell that is a spatial singlet. **f**, Violin plot showing the proportion of spatial barcode UMIs that are assigned to the DBSCAN singlet cluster (signal) vs. all other spatial barcode UMIs recovered for that cell. **g**, Violin plot showing the mean radial distance for spatial barcodes for each spatial singlet cluster. **h**, The proportion of cells that are assigned to each number of DBSCAN clusters. **i**, plot showing the concentration of oligos released by time under illumination at the same light source power, for each bead type used in Slide-tags experiments. The time used for cleavage for each bead type is shown with the dotted lines. **j**, Plot showing the standard error (SE) for each singlet true spatial barcode cluster in x and y. Density shows: centre line, median; adjacent lines, upper and lower quartiles. **k**, The full set of spatial barcodes recovered for each of the nuclei plotted in Fig. 1c, with their xy positions, kernel density estimates, and coloured by nUMI associated with each cell are plotted (top). Points centred around the signal cluster are shown at higher magnification with final cell position shown as a cross (bottom). Scale bars denotes 500 μm, except for magnified plots, where scale bars denote 200 μm. Boxplots show: centre line, median; box limits, upper and lower quartiles; whiskers, 1.5x interquartile range; points, outliers. CA1 = Cornu Ammonis area 1, DG = dentate gyrus. a,j, n = 839 nuclei. d-g, n = 1042 nuclei. b,c,h, n = 1889 nuclei.

**Extended Data Fig. 3:**
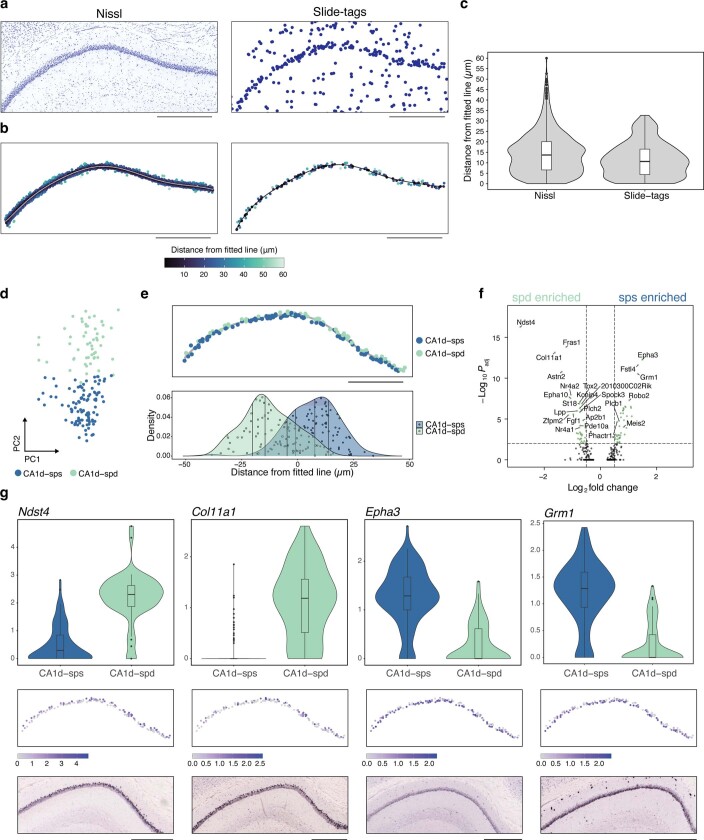
Spatial resolution measurements in the mouse hippocampus and Slide-tags snRNA-seq enables characterization of the deep and superficial sublayers in the mouse hippocampal CA1 field. **a**, A 10 um nissl-stained section (left) was taken adjacently to the Slide-tags profiled section (right). **b**, The CA1 nuclei were subsetted in each case and a line was fitted to measure the midpoint of this structure. For Slide-tags, nuclei were selected based on their cell type assignment in Figure S1, with 2 spatial outliers removed. For Nissl, nuclei were computationally segmented. Orthogonal distances from this midpoint were then calculated and points are coloured by this distance. **c**, Violin plots showing the distribution of distances from the fitted line in b. **d**, PCA plot showing cells from the CA1 cluster after subsetting, reprojection, and reclustering. Cells are coloured according to their new sub-cluster assignment. **e**, Cells from a are plotted according to their spatial location (top). The spatial density of nuclei from each population is plotted (bottom). **f**, Volcano plot showing differentially expressed genes between sps and spd. **g**, Violin plots showing gene expression differences between each subcluster (top) and the expression of these genes spatially (middle), as well as in situ hybridization data (bottom) from Allen Mouse Brain Atlas^23^. Genes were selected based on being discovered as differentially expressed between these two sub-clusters in our dataset and also identified in previous studies as defining these two sub-layers^68,69^. Boxplots show: centre line, median; box limits, upper and lower quartiles; whiskers, 1.5x interquartile range; points, outliers. All scale bars denote 500 μm. For Slide-tags CA1, n = 155 nuclei. For imaging data, n = 898 segmented nuclei.

**Extended Data Fig. 4:**
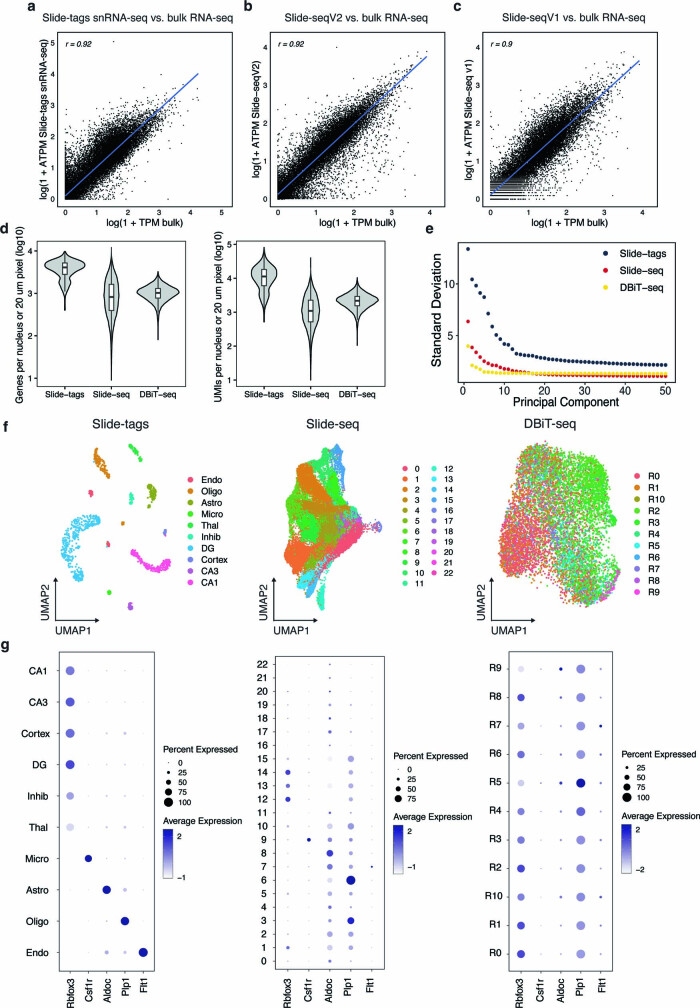
Comparison of Slide-tags to Bulk RNA-seq, Slide-seqV2 and DBiT-seq. **a**, Slide-tags snRNA-seq vs. bulk RNA-seq. **b**, Slide-seqV2 vs. bulkRNA-seq. **c**, Slide-seqV1 vs. bulkRNA-seq. Log_10_ transformations are shown in each bulk comparison case. **d**, Violin plots of log_10_-transformed genes and UMIs per nucleus (Slide-tags) or 20 μm spatial spot (Slide-seqV2 and DBiT-seq / spatial-ATAC-RNA-seq) in the mouse brain. n = 839 nuclei for Slide-tags, n = 18,950 20 μm pixels for Slide-seq, and n = 9,215 pixels for DBiT-seq. **e**, Elbow plot of standard deviations of principal components from Slide-tags snRNA-seq, Slide-seqV2, and DBiT-seq in the mouse brain. **f**, UMAP embeddings of snRNA-seq profiles from Slide-tags snRNA-seq (cell type labels), Slide-seqV2 (de novo clusters), and DBiT-seq (RNA clusters from Zhang et al.^13^) in the mouse brain. **g**, Dotplot expression of select markers across transcriptome clusters from Slide-tags snRNA-seq, Slide-seqV2, and DBiT-seq in the mouse brain. Boxplots show: centre line, median; box limits, upper and lower quartiles; whiskers, 1.5x interquartile range; points, outliers.

To benchmark our approach, we performed Slide-tags followed by droplet-based snRNA-seq on a 20 μm coronal section of the adult mouse hippocampus, which has a highly stereotyped architecture that is useful for validating spatial techniques^9^. We dissociated and sequenced 1,661 nuclei from a 3 mm^2^ area coronal tissue section, clustering the data using a standard single-cell pipeline^21^ (Fig. 1b) and annotating clusters using well-established cell class markers (Extended Data Fig. 1). Multiple spatial barcodes were detected per nucleus, enabling higher assignment confidence than when using protocols in which only one spatial barcode is associated with a cell (Fig. 1c). To spatially position our single-nucleus transcriptomes, we used density-based spatial clustering of applications with noise (DBSCAN)^22^ to separate background spatial barcodes from the true signal (Methods, Extended Data Fig. 2 and Supplementary Fig. 1). Nuclei are then assigned a spatial coordinate using the unique molecular identifier (UMI)-weighted centroid of the DBSCAN-clustered spatial barcodes denoted the true signal (Methods). Using this procedure, we assigned spatial locations to 839 high-quality nucleus profiles (50.5% of profiled nuclei, 11,250 median UMIs per nucleus). Examination of the spatial positions of individual clusters recapitulated the expected cytoarchitectural arrangement of the hippocampus (Fig. 1d). Furthermore, spatial expression profiles of individual genes matched existing in situ hybridization data^23^ (Fig. 1e). To quantify spatial positioning accuracy, we first compared the width of the hippocampal subfield cornu ammonis area 1 (CA1) in Slide-tags with a Nissl-stained serial section and found that the width of the Slide-tags feature was congruent with the Nissl image (Extended Data Fig. 3). Moreover, we found that we could accurately localize sub-cell types in the deep and superficial layers of the CA1 (Extended Data Fig. 3 and Supplementary Table 1). Second, we calculated the standard error for each centroid in *x* and *y*, and estimated the accuracy to be 3.5 ± 1.9 μm in *x* and 3.6 ± 2 μm in *y* (mean ± s.d., *n* = 839 nuclei; Extended Data Fig. 2j). Third, we quantified the nucleus misassignment rate by leveraging the stereotyped structure of the CA1 and dentate gyrus. We found that 98.7% of CA1 (155 out of 157) and dentate granule (312 out of 316) neurons were localized in the CA1 pyramidal layer and the dentate gyrus, respectively (Extended Data Fig. 1b). We investigated whether the tagging procedure affected the resultant snRNA-seq data quality by comparing standard snRNA-seq with Slide-tags followed by snRNA-seq on adjacent sections of the mouse hippocampus. We found that recovered cell type proportions (Pearson’s *r* = 0.96, *P* < 2.2 × 10^−16^), UMIs recovered per cell (Pearson’s *r* = 0.96, *P* < 2.2 × 10^−16^) and gene expression (Pearson’s *r* = 0.99, *P* < 2.2 × 10^−16^) were all unaffected by the tagging procedure (Fig. 1g–i). Slide-tags is also well correlated to bulk-RNA-seq from the same tissue region (Extended Data Fig. 4a; Pearson’s *r* = 0.92). Thus, Slide-tags generated data that are almost indistinguishable from snRNA-seq with a theoretical ~3 μm spatial localization accuracy.

**Extended Data Fig. 5:**
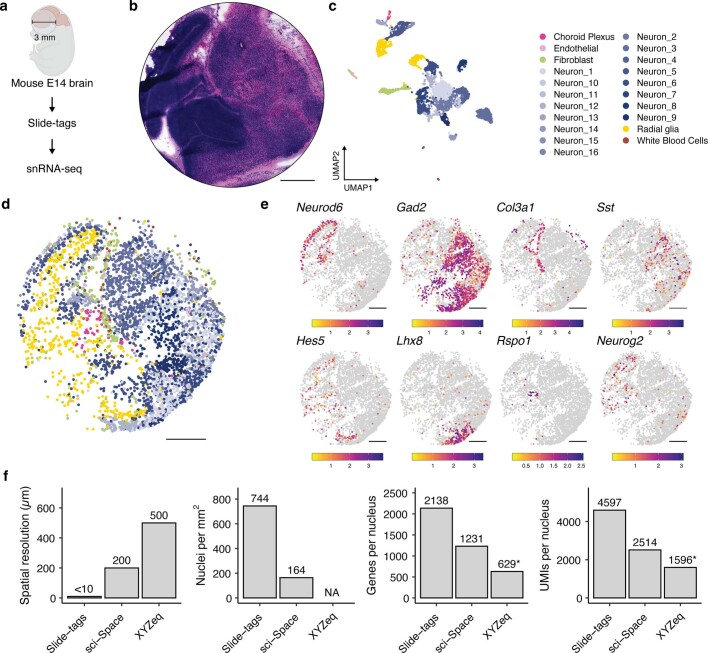
Slide-tags snRNA-seq applied to the embryonic mouse brain at E14. **a**, Schematic of Slide-tags snRNA-seq on a 3 mm diameter region of the embryonic mouse brain at E14. **b**. A haematoxylin and eosin stained section which was adjacent to the profiled section. **c**. UMAP embedding of snRNA-seq profiles coloured by cell-type annotations. **d**. Spatial positions of cells coloured as in C. **e**. Spatial marker gene expression. Expression counts for each cell were divided by the total counts for that cell and multiplied by 10,000, this value + 1 is then natural-log transformed. **f**. Comparison metrics plotted for Slide-tags snRNA-seq on the mouse E14 embryonic brain. * = XYZeq was not performed on embryonic mouse brain at E14 and so these metrics may not be directly comparable due to tissue-specific effects. All scale bars denote 500 μm.

We next performed Slide-tags snRNA-seq on a 7 mm^2^ area sagittal section of the embryonic mouse brain at embryonic day 14 (E14; Extended Data Fig. 5a,b), which has been frequently used for benchmarking new spatial transcriptomics technologies^10,20^. We sequenced and spatially positioned 4,584 nuclei (4,594 median UMIs per nucleus), which we clustered and annotated by cell type (Extended Data Fig. 5c–e). Compared with existing approaches, sci-Space and XYZ-seq, for single-cell spatial placement, Slide-tags achieved 20–50-fold higher spatial resolution and recovered 4.5-fold more nuclei per unit area. We also recovered 1.8-fold more UMIs and 1.7-fold more genes per nucleus than adjacent technologies at a sequencing saturation of 48% (Extended Data Fig. 5f).

Finally, we also benchmarked Slide-seq performance in relation to Slide-seq and DBIT-seq in the adult mouse brain. We found that Slide-tags achieves a significantly higher molecular sensitivity (13,142 transcripts per nucleus versus 1,702 and 2,538 transcripts per 20 μm^2^ pixel for Slide-seq (binned) and DBIT-seq, respectively; Extended Data Fig. 4)). Note that, even in high-resolution capture-based spatial transcriptomics, pixels capture mixtures of transcriptomes from nearby cells, hindering unsupervised clustering of cell type identity and marker gene identification (Extended Data Fig. 4).

## Slide-tags snRNA-seq in the human cortex

**Fig. 2:**
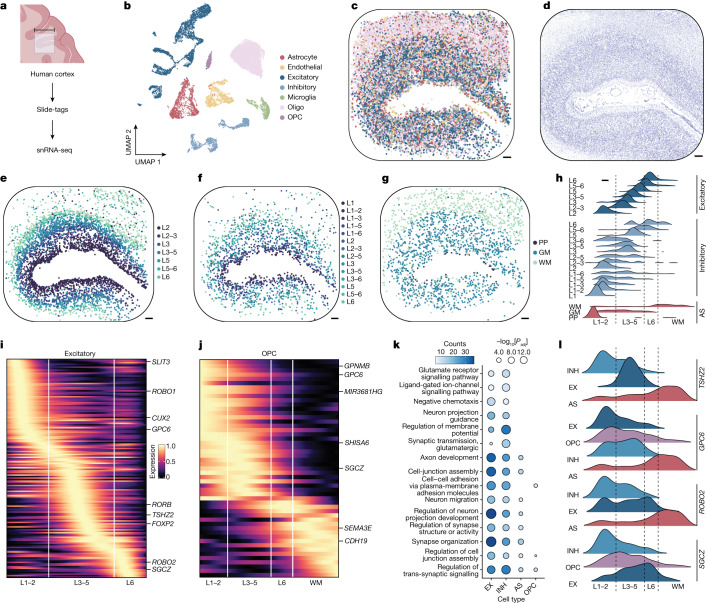
Spatially resolved cell-type-specific expression in the human brain using Slide-tags snRNA-seq. **a**, Schematic of Slide-tags snRNA-seq analysis of a 10-mm square region of the human prefrontal cortex. Scale bar, 10 mm. The diagram was created using BioRender. **b**, UMAP embedding of snRNA-seq profiles, coloured according to cell type assignment. **c**, Spatial mapping of snRNA-seq profiles, coloured by cell type as in **b**. **d**, A Nissl-stained tissue section adjacent to the profiled section. **e**, Spatial mapping of grouped excitatory neuron subtypes. L1–6, cortical layers 1–6. **f**, Spatial mapping of grouped inhibitory neuron subtypes. **g**, Spatial mapping of astrocyte (AS) subtypes. GM, grey matter; PP, protoplasmic; WM, white matter. **h**, The layer specificity of grouped excitatory neuron, grouped inhibitory neuron and AS subtypes. **i**,**j** Heat maps of one-dimensional gene expression for excitatory neurons (**i**) and OPCs (**j**). **k**, Gene Ontology analysis of the highest spatially variable genes in each cell type. EX, excitatory; INH, inhibitory; *P*_adj_, adjusted *P* value. **l**, The spatial expression of genes with contrasting gradients across cell types. For **c**–**g**, scale bars, 500 μm.

**Extended Data Fig. 6:**
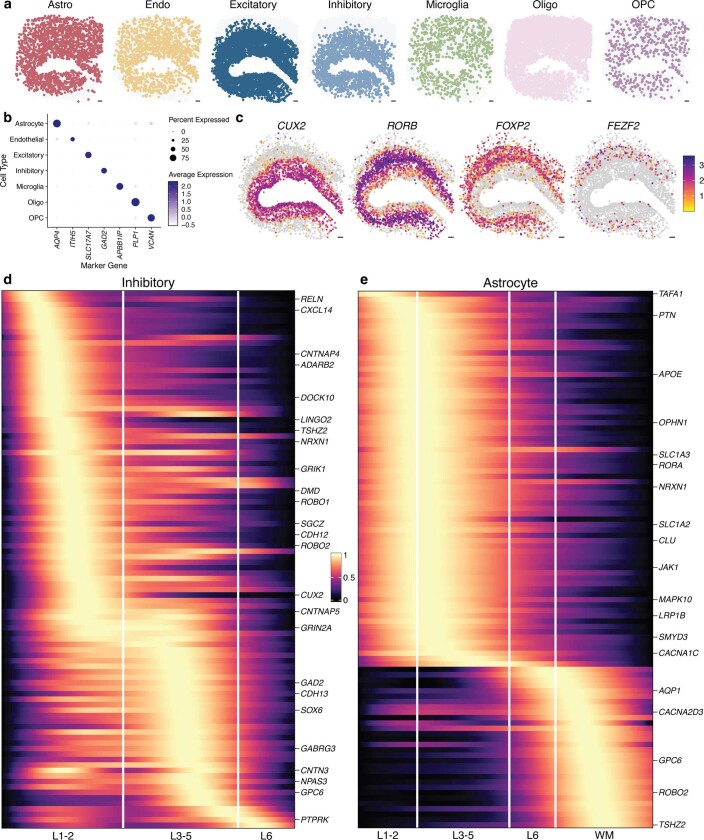
Slide-tags snRNA-seq applied to the human brain enables spatial mapping of cell types and cell-type specific spatially varying gene expression. **a**, Individual plots of per-cell type spatial distribution. The diagram was created using BioRender. **b**, Dotplot showing the marker genes used to assign cell types to clusters. **c**, The gene expression distribution of four canonical layer marker genes in excitatory neurons. **d,e**, A 1D gene expression heatmap for genes in inhibitory neurons and astrocytes. All scale bars represent 500 µm. Oligo = Oligodendrocyte, OPC = Oligodendrocyte precursor cell, Astro = Astrocyte, Endo = Endothelial, WM = White matter. Gene names and details in Supplementary Table 2.

The human cerebral cortex has a well-characterized cytoarchitecture in which specific subpopulations of neurons are arranged in discrete layers. Existing spatial sequencing approaches can resolve broad patterns of spatially varying gene expression in human cortex^24^, but assignment of spatially variable genes to specific cell types is challenging using these methods. We reasoned that Slide-tags could be used for facile profiling of human brain tissue, most especially to discover cell-type-specific spatial gene expression patterns. We profiled a 100 mm^2^ region of the human prefrontal cortex from a neurotypical donor aged 78 years (Methods), recovering 17,441 high-quality spatially mapped nuclei with a median of 3,196 UMIs per nucleus (Fig. 2a). Clustering analysis revealed the expected neuronal and glial cell types, recapitulating known layer distributions and spatial structures (Fig. 2b–d and Extended Data Fig. 6a,b). We computationally integrated (Methods) an existing snRNA-seq dataset^25^ that includes layer annotations for 91 neuron subtypes, recovering the expected spatial distributions across subtypes (Fig. 2e,f and Supplementary Figs. 2 and 3). Similarly, astrocytes could be clustered into two distinct populations that spatially segregated between white and grey matter regions (Fig. 2g). Quantification of the laminar position of each of these excitatory, inhibitory and astrocytic populations showed them to be accurately positioned within the white matter and cortical layers (Fig. 2h).

We next used our whole-transcriptome, spatially resolved snRNA-seq profiles to systematically identify spatially varying genes in each cell type. We plotted the layer distributions of the highest spatially varying genes (Methods and Supplementary Table 2) for excitatory neurons (Fig. 2i and Supplementary Fig. 4), recovering many well-known laminar markers such as *CUX2*, *RORB* and *FOXP2* (Extended Data Fig. 6c), as well as for inhibitory neurons (Extended Data Fig. 6d and Supplementary Fig. 5a) and astrocytes (Extended Data Fig. 6e and Supplementary Fig. 6). Notably, we also identified spatially varying genes within oligodendrocyte precursor cells (OPCs), which had not previously been known to have areal specializations (Fig. 2j and Supplementary Fig. 5b). Gene Ontology analysis of these spatially varying genes revealed a relationship with biological processes including cell–cell adhesion, cell junction assembly and axon development (Fig. 2k and Supplementary Table 3).

Genes can show spatially variable expression that may derive from several cell types, but assigning such expression variability to individual cell types can be very challenging using traditional spatial transcriptomics approaches owing to the mixing of individual pixels. Among our spatially varying genes, we identified several that were variable across multiple cell types, such as *SGCZ*, of which the spatial expression variation in excitatory and inhibitory neurons was anticorrelated, and showed an orthogonal spatial distribution in OPCs (Fig. 2l). We performed additional Slide-tags snRNA-seq analysis of the human cortex, from this donor and another donor, and found that the nucleus mapping rate and subsequent density were congruent (Supplementary Table 4). Together, these results demonstrate the ability of Slide-tags to reproducibly and systematically uncover transcriptional variation within the cytoarchitecturally complex tissues of the human brain.

## Slide-tags snRNA-seq analysis of the human tonsil

**Fig. 3:**
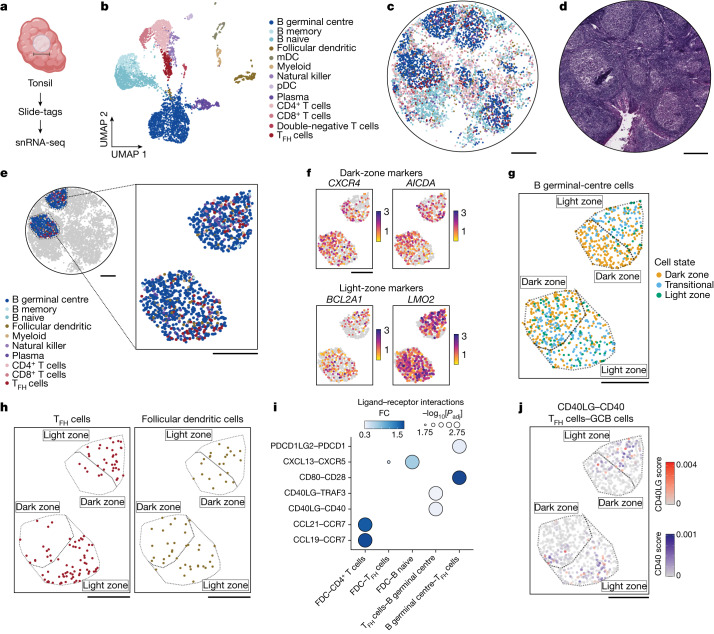
Slide-tags enables cell-type-specific spatially varying gene expression analysis and spatial receptor–ligand interaction prediction within the human tonsil. **a**, Schematic of Slide-tags snRNA-seq analysis of a 3-mm-diameter region of human tonsil tissue. Scale bar, 3 mm. The diagram was created using BioRender. **b**, UMAP embedding of snRNA-seq profiles coloured by cell type annotations. mDC, myeloid dendritic cells; pDC, plasmacytoid dendritic cells; T_FH_ cells, T follicular helper cells. **c**, Spatial mapping of snRNA-seq profiles, coloured by cell type as in **b**. **d**, Adjacent haematoxylin and eosin (H&E)-stained section of the profiled region. **e**, Magnified view of two germinal centres coloured by cell type. **f**, Expression of dark-zone and light-zone marker genes identified as spatially varying within germinal centres. **g**, GCB cell state classification and zone segmentation on the basis of the cluster density of dark-zone GCB cells. **h**, Spatial mapping of T_FH_ cells and follicular dendritic cells on zoned germinal centres. **i**, Selected spatially co-occurring receptor–ligand interactions within certain sender–receiver cell type pairs. **j**, Spatial mapping of interaction intensity scores for CD40 in GCB cells and CD40LG in T_FH_ cells. For **c**–**h** and **j**, scale bars, 500 μm.

**Extended Data Fig. 7:**
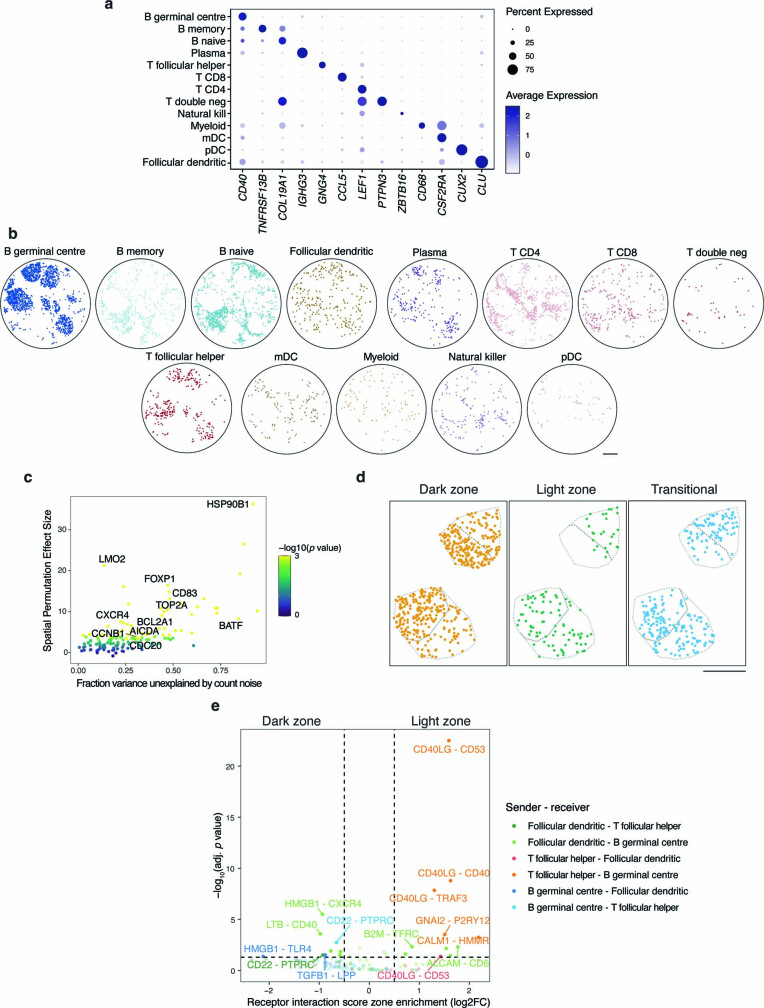
Receptor-ligand prediction from Slide-tags human tonsil data. **a**, Expression of select marker genes by cell type cluster. **b**, Spatial mapping of cell types. **c**, Scatter plot of gene expression variance not explained by count noise and spatial permutation effect size of previously reported dark zone and light zone marker genes. **d**, Spatial mapping of dark zone, light zone, and transitional germinal centre B cells in two representative germinal centres. **e**, Volcano plot of receptor interaction intensity scores compared between zones in two representative germinal centres. All scale bars denote 500 μm. T double neg = T double negative, mDC = myeloid dendritic cells, pDC = plasmacytoid dendritic cells.

A key challenge for spatial genomics technologies is the proper segmentation of densely packed tissues, such as those of immune origin. We reasoned that Slide-tags would be ideal in this setting, given that segmentation is accomplished automatically by dissociating the tissue into individual nuclei. We therefore performed Slide-tags snRNA-seq analysis of the human tonsil (Fig. 3a–d), recovering 81,000 nuclei after dissociation from 7 mm^2^ of tissue. We sequenced 8,747 of these nuclei, spatially mapping 5,778 high-quality snRNA-seq profiles (2,377 median UMIs per nucleus and 1,557 median genes per nucleus). Clustering of the data identified subpopulations of B and T cells, some of which are known to be spatially segregated (Extended Data Fig. 7a,b). Indeed, examination of the spatial positions of these clusters revealed the expected spatial architecture of the tissue, with B and T cell zones, as well as germinal centres composed of germinal centre B (GCB) cells, T follicular helper cells and follicular dendritic cells (Fig. 3c,d and Extended Data Fig. 7b). Subclassification of GCB cells into light-zone and dark-zone GCB cells is challenging using snRNA-seq data alone, as variation in gene expression space is low, requiring many cells to be sampled to uncover the distinction^26^. However, as reactive germinal centres are spatially polarized into light zones and dark zones, we reasoned that we could classify GCB cells by harnessing the combined spatial and single-cell data. To do so, we computed spatially varying genes within GCB cells through spatial permutation testing^20^, identifying key markers of light-zone and dark-zone GCB cells (Fig. 3e,f and Supplementary Table 5). Dark-zone marker genes included *CXCR4* (double-sided permutation test, *Z* score = 7.6, *P* < 0.001) and *AICDA* (*Z* score = 6.9, *P* < 0.001)—genes associated with dark-zone organization and somatic hypermutation, respectively^27–29^. Light-zone-enriched genes included *BCL2A1* (*Z* score = 9.1, *P* < 0.001), an apoptosis regulator gene^30^, and *LMO2* (*Z* score = 21.3, *P* < 0.001), a transcription factor^31^. A subset of expected light-zone and dark-zone markers had relatively low variance in gene expression, but high spatial permutation effect sizes, demonstrating that spatial positions enhance interpretation of transcriptomic profiles (Extended Data Fig. 7c and Supplementary Table 6). Reclustering GCB cells on the basis of spatially varying genes enabled classification into dark-zone, light-zone and transitional cell states (Fig. 3g and Methods). We then segmented the two largest profiled germinal centres into light zones and dark zones through spatial clustering of dark-zone GCB cells, the most abundant GCB cell state (Extended Data Fig. 7d). In corroboration of our zone segmentation, we found that T follicular helper cells were enriched in light zones while follicular dendritic cells were dispersed between the light zone and the dark zone (Fig. 3h; *χ*^2^ = 43.7, *P* = 3.7 × 10^−11^ (T follicular helper cells); and *χ*^2^ = 0.58, *P* = 0.45 (follicular dendritic cells)).

Immune cells engage in extensive cross-talk within and around germinal centres^32^. We wondered whether Slide-tags could reveal receptor–ligand interactions that drive such intercellular communication. We first nominated putative receptor–ligand interactions in a spatially agnostic manner using LIANA^33^. We next incorporated spatial information by performing a spatial permutation test to identify interactions that significantly co-occur spatially (Methods). Using this approach, we predicted 645 receptor–ligand interactions, many of which are well-characterized axes of communication during B cell maturation (Fig. 3i and Supplementary Table 7). For example, we predicted interactions between CD40 and CD40LG within GCB cells and T follicular helper cells, a fundamental driver of the germinal-centre reaction^34^. We also identified downstream targets of canonical receptor–ligand interactions, such as TRAF3, important in regulating the intracellular effects of CD40–CD40LG binding^35^.

Finally, we reasoned we could spatially contextualize receptor–ligand interactions within native tissue niches. Our predicted interactions can be decomposed into interaction intensity scores for individual cells based on expression and spatial co-occurrence of the receptor and ligand. For the 99 nominated receptor–ligand pairs between GCB cells, follicular dendritic cells and T follicular helper cells, we used our germinal-centre zone segmentations to assess light-zone and dark-zone enrichment in predicted interaction intensity. We revealed light-zone enrichment of 11 interactions and dark-zone enrichment of 9 interactions (Extended Data Fig. 7e and Supplementary Table 8). GCB CD40 receptor in interaction with T follicular helper cell CD40LG was highly enriched in light zones (Fig. 3j; Wilcoxon rank-sum test, log_2_[fold change (FC)] = 1.6, adjusted *P* (*P*_adj_) = 1.6 × 10^−9^), whereas CD40 receptor expression alone was modestly dark-zone biased (Wilcoxon rank-sum test, log_2_[FC] = −0.04, *P* = 0.047). We also revealed zone-biased interactions with lesser-known importance in the germinal-centre reaction, such as the light-zone-enriched interaction between T follicular helper cell CD40LG and GCB CD53 (Extended Data Fig. 7e; Wilcoxon rank-sum test, log_2_[FC] = 1.6, *P* = 2.3 × 10^−23^). Together, Slide-tags enabled spatial contextualization of cell-type-specific receptor–ligand interactions that are not obvious by analysis of expression alone.

## Slide-tags multiome of human melanoma

Epigenetic dysregulation in cancer facilitates drug resistance and pro-metastatic cell state transitions^36–38^. Numerous studies of tumour heterogeneity have revealed clone-specific niches and immune compartments^7,39,40^, but the role of epigenetic regulation in establishing and maintaining these spatial niches remains difficult to study. Concurrent spatial mapping of the genome, transcriptome and epigenomic landscape of the tumour microenvironment could offer insights into the complex mechanisms of tumour evolution. We therefore developed Slide-tags multiome, enabling simultaneous single-cell spatial profiling of mRNA and chromatin accessibility, along with CNV inference.

**Fig. 4:**
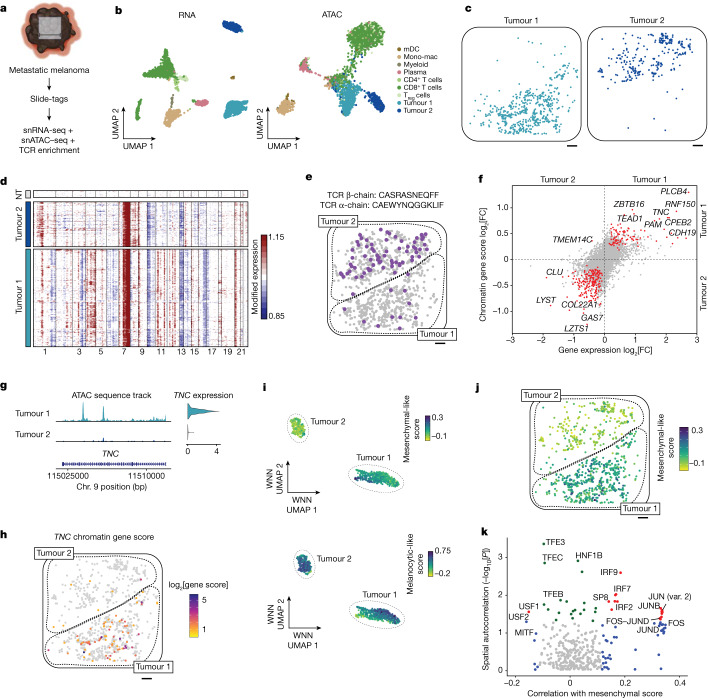
Multiomic Slide-tags captures spatially resolved clonal relationships between single nuclei in human melanoma. **a**, Schematic of joint snATAC–seq and snRNA-seq analysis of a 5.5-mm square region of a human melanoma lymph node metastasis. Scale bar, 5.5 mm. The diagram was created using BioRender. **b**, UMAP embeddings of snRNA-seq and snATAC–seq profiles coloured by cell type. Mono-mac, monocyte-derived macrophages, T_reg_ cells, regulatory T cells. **c**, Spatial mapping of tumour cluster 1 and tumour cluster 2. **d**, Inferred copy-number alterations from transcriptomic data. NT, a representative subset of non-tumour cells. **e**, Spatial mapping of a TCR β-chain clonotype expanded in the tumour cluster 2 compartment, with the matched α-chain indicated above. Grey cells show the positions of all CD8^+^ T cells. **f**, Differential gene expression and differential chromatin gene scores between tumour cluster 1 and tumour cluster 2. The red points have *P*_adj_ < 0.05 for both tests. **g**, Genome coverage track and gene expression violin plot of *TNC* between tumour clusters. The range of the normalized chromatin accessibility signal is 0–50. Chr., chromosome. **h**, The spatial distribution of *TNC* chromatin accessibility gene scores. Gene scores are log_2_-transformed. **i**, Weighted nearest-neighbour (WNN) UMAP embedding of tumour cells, with cells coloured according to mesenchymal-like and melanocytic-like cell state scores. **j**, Spatial mapping of mesenchymal-like cell state scores in tumour cells. **k**, Spatial autocorrelation of accessibility in chromVAR transcription factor motifs correlated with mesenchymal-like cell state scores. The red points indicate spatial autocorrelation Moran’s *I* raw *P* < 0.05 and significant correlation with mesenchymal-like score (*P*_adj_ < 0.05). The green points indicate only spatial autocorrelation raw *P* < 0.05. The blue points indicate only significant correlation with mesenchymal-like score (*P*_adj_ < 0.05). Only chromVAR transcription factor motifs with a positive Moran’s *I* are shown. For **c**, **e**, **h** and **j**, scale bars, 500 μm.

**Extended Data Fig. 8:**
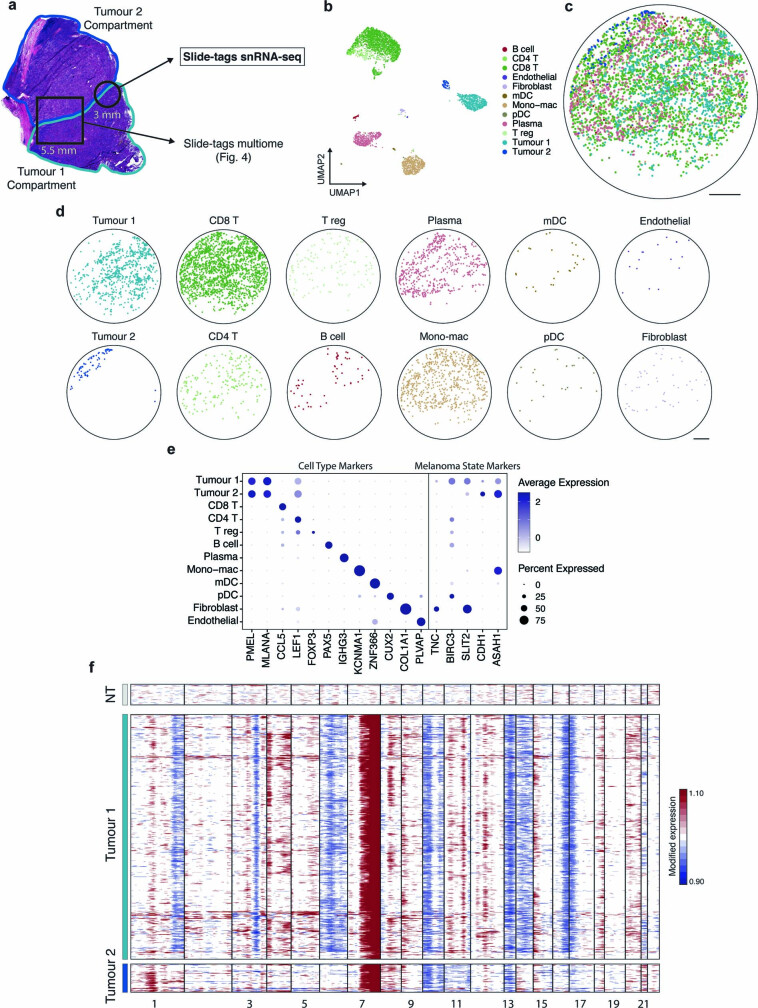
Slide-tags snRNA-seq on human melanoma. **a**, Schematic representation of Slide-tags snRNA-seq and Slide-tags multiome (Fig. 4) profiled regions across tumour 1 and 2 compartments. **b**, UMAP embeddings of snRNA-seq profiles coloured by cell type. **c**, Spatial mapping of cell types. **d**, Spatial mapping of profiled cell types. **e**, Expression of select cell type marker genes and melanoma cell state marker genes. **f**, Inferred copy number alterations from transcriptomic data. NT indicates a representative subset of non-tumour cells. All scale bars denote 500 μm. T reg = T regulatory cells, mDC = myeloid dendritic cells, Mono-mac = monocyte-derived macrophages, pDC = plasmacytoid dendritic cells.

**Extended Data Fig. 9:**
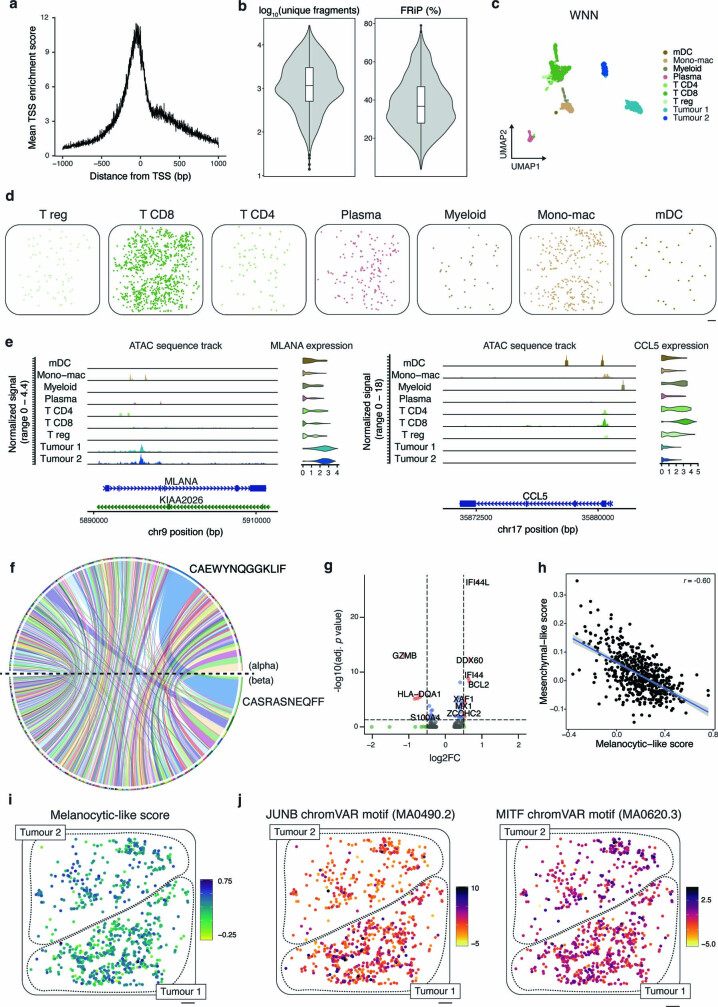
Slide-tags multiome on human melanoma. **a**, Mean TSS enrichment score. **b**, Violin plots of log_10_-transformed unique fragments and fraction of reads in peaks (FRiP) percentage. n = 2,529 nuclei. Boxplots show: centre line, median; box limits, upper and lower quartiles; whiskers, 1.5x interquartile range; points, outliers. **c**, Weighted nearest neighbour UMAP embeddings of snRNA-seq and snATAC-seq profiles coloured by cell type. **d**, Spatial mapping of cell types. **e**, ATAC sequence track and gene expression violin plot of *MLANA* and *CCL5* across cell types. **f**, TCR pairing chord plot of alpha and beta chain pairing frequencies in CD8 T cells. **g**, Differential gene expression volcano plot between CD8 T cells in tumour compartment 1 vs tumour compartment 2. **h**, Scatter plot of melanocytic-like scores and mesenchymal-like scores of tumour cluster 1 cells in tumour compartment 1. Pearson’s *r* value is reported. Error band represents the 95% confidence interval. **i**, Mesenchymal-like cell state score spatial distribution. **j**, Spatial distribution of JUNB and MITF chromVAR motif scores. All scale bars denote 500 μm. T reg = T regulatory cells, mDC = myeloid dendritic cells, Mono-mac = monocyte-derived macrophages.

We first performed Slide-tags snRNA-seq analysis of a metastatic melanoma sample (Extended Data Fig. 8a–f). We recovered 10,960 nuclei after dissociation from 7 mm^2^ of tissue, sequencing 6,464 of these nuclei and spatially mapping 4,804 high-quality snRNA-seq profiles (2,110 median UMIs per nucleus and 1,317 median genes per nucleus). In an adjacent section, we applied Slide-tags multiome, profiling the tagged nuclei using droplet-based combinatorial snATAC–seq and snRNA-seq (Fig. 4a,b). We spatially mapped 2,529 nuclei from a 38.3 mm^2^ section and both modalities displayed high-quality data on the basis of basic technical performance metrics (Fig. 4b,c and Extended Data Fig. 9a–e; median UMIs per nucleus = 5,228, median genes per nucleus = 2,429, transcription start site enrichment score = 11.5, median fragments per nucleus = 1,159, median fraction of unique fragments in peaks = 36.7%).

Unsupervised clustering of snRNA-seq and multiome data identified immune, stromal and tumour cell types (Fig. 4b and Extended Data Fig. 8d,e). The tumour cells were split into two subpopulations, denoted as tumour cluster 1 and tumour cluster 2, that segregated into spatially distinct compartments (Fig. 4b,c and Extended Data Fig. 8d). As CNV has an important role in melanoma tumour evolution^41,42^, we sought to identify whether these transcriptional subpopulations represented distinct genetic clones. We inferred copy-number alterations using inferCNV^43^, a standard scRNA-seq CNV inference tool, from the transcriptomes of each spatially mapped nucleus (Methods). Indeed, across both the snRNA-seq and the multiome data, we uncovered genomic differences consistent with the spatial and transcriptional separation between tumour cluster 1 and 2 (for example, CNV on chromosome 6; Fig. 4d and Extended Data Fig. 8f).

**Extended Data Fig. 10:**
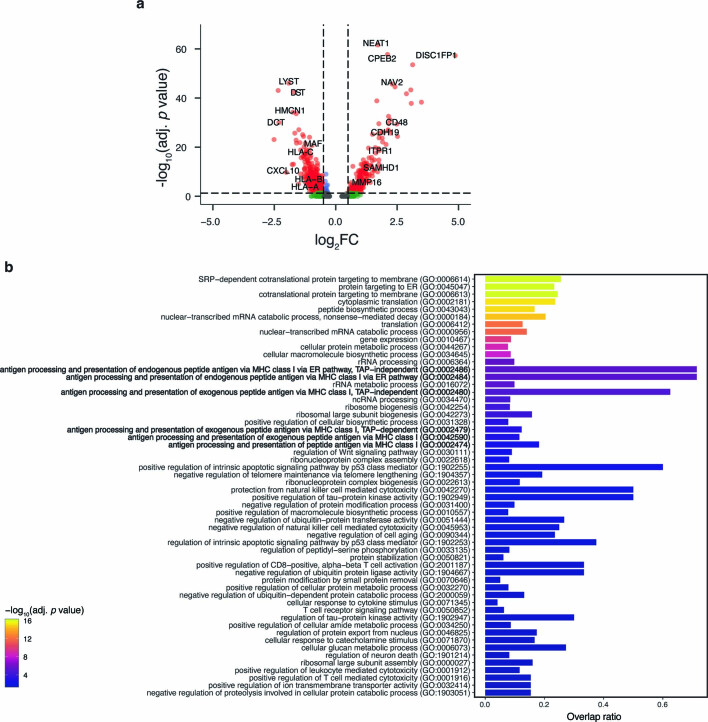
Differential gene expression and gene set enrichment analysis between tumour cluster 1 and 2. **a**, Volcano plot of differentially expressed genes comparing tumour cluster 1 against tumour cluster 2 from the Slide-tags snRNA-seq run. **b**, Gene ontology biological process (GO_Biological_Process_2021) gene set enrichment analysis on genes upregulated in tumour cluster 2 (negative log_2_FC) compared with tumour cluster 1 from the Slide-tags snRNA-seq experiment.

Our basic clustering analysis showed extensive T cell infiltration into both tumour compartments (Extended Data Figs. 8d and 9d). We wondered whether there might exist heterogeneous T cell responses to these genetically distinct compartments. First, we enriched for TCR sequences in our 1,020 spatially positioned CD8^+^ T cell cDNA profiles, recovering 419 cells with α-chains (279 unique), 761 cells with β-chains (410 unique) and 358 cells with paired α- and β-chains (265 unique) (Supplementary Table 9). We found a TCRβ clonotype that was significantly expanded in tumour compartment 2 compared with in tumour compartment 1 (Fig. 4e; Fisher’s exact test, odds ratio = 6.8, *P* = 1.1 × 10^−11^), in agreement with our previous report^44^. Given our high TCR pairing rate (Extended Data Fig. 9f), we also noted tumour compartment 2 expansion of CD8^+^ T cells with this β-chain and a paired α-chain (Fisher’s exact test, odds ratio = 11.9, *P* = 9.6 × 10^−6^). We observed that CD8^+^ T cells in tumour compartment 2 were upregulated in cytotoxic *GZMB* expression (Extended Data Fig. 9g and Supplementary Table 10). In addition to this T cell variation, we noted decreased expression of MHC class I endogenous antigen presentation genes in tumour cluster 1 relative to tumour cluster 2 (Extended Data Fig. 10 and Supplementary Table 11; gene set enrichment analysis, GO:0002484; overlap ratio = 0.71; *P*_adj_ = 6.6 × 10^−6^), potentially contributing to differential T cell clone infiltration between the tumour compartments. Thus, we observed a cytotoxic T cell clone specifically infiltrating into a spatially and genetically distinct tumour compartment. Although TCR expression has previously been spatially mapped^44,45^, Slide-tags enables unambiguous assignment of receptor pairs to single cells.

To further investigate how chromatin accessibility and transcription informs tumour cell state and how this relates to the tumour microenvironment, we identified spatially segregated differential gene expression and differential chromatin gene scores between tumour subpopulations (Fig. 4f and Supplementary Table 12). *TNC* and other mesenchymal-like cell state markers were found to be differentially expressed (Methods and Supplementary Figs. 7 and 8; log_2_[FC] = 2.1, *P*_adj_ = 2.4 × 10^−61^) and differentially accessible by chromatin gene score (Wilcoxon rank-sum test, log_2_[FC] = 0.81, *P*_adj_ = 1.0 × 10^−12^) in tumour cluster 1 compared with in tumour cluster 2 (Fig. 4g,h). We observed heterogeneity in *TNC* chromatin accessibility and gene expression within tumour cluster 1, which has previously been associated with a mesenchymal-like cell state^46,47^. We therefore hypothesized that tumour cluster 1 may comprise two cell states: melanocytic like and mesenchymal like. We scored tumour cells for melanocytic-like and mesenchymal-like cell states using genes that were previously implicated in this transition^46^. While tumour cluster 2 was largely a melanocytic-like population, we observed melanocytic-like and mesenchymal-like scores were negatively correlated and heterogeneous in tumour cluster 1 (Fig. 4i,j and Extended Data Fig. 9h,i; Pearson’s *r* = −0.60, *P* < 2.2 × 10^−16^). To uncover *trans*-acting factors associated with this transition, we first identified accessible transcription factor motifs that were correlated with mesenchymal-like score within tumour cluster 1 using chromVAR^48^ (Fig. 4k (*x* axis) and Supplementary Table 13; *P*_adj_ < 0.05); positively correlated transcription factor motifs included FOS/JUN-family members, which have previously been implicated in mesenchymal-like melanoma states, and IRF-family transcription factors. Negatively correlated transcription factor motifs included MITF, a factor involved in maintaining the melanocytic lineage^49,50^. Although such epigenomic signatures driving mesenchymal-like state have previously been identified in single cells, their localization within tissues is lacking. To answer whether such epigenetic signatures were spatially non-random, we performed spatial autocorrelation analysis of transcription factor motif scores in the tumour cluster 1 compartment (Fig. 4k (*y* axis) and Extended Data Fig. 9j). The top spatially autocorrelated transcription factor motifs associated with a mesenchymal-like state were JUN-, FOS and IRF family members with positive autocorrelation scores, suggesting that these epigenomic signatures are locally clustered. Local clustering of epigenetic states is suggestive of inheritance of epigenetically reprogrammed states in cell division, or local signalling environmental drivers^37,51^.

## Discussion

Here we developed Slide-tags, a spatial single-nucleus genomics technology that is widely applicable to tissues spanning different scales, species and disease states. We profiled Slide-tags nuclei isolated from the mouse and human adult brain using snRNA-seq, showing indistinguishable RNA data quality and high spatial positioning accuracy, and identifying cell-type-specific spatially varying genes across cortical layers. Applying Slide-tags snRNA-seq to densely packed human tonsil enabled spatial contextualization of predicted receptor–ligand interactions. Finally, to demonstrate the multimodal capacity of Slide-tags, we simultaneously profiled the transcriptome, epigenome and TCR repertoire of metastatic melanoma tissue, and inferred CNV from transcriptome data. We inferred copy-number alterations from transcriptome data and revealed spatial immune cell differences between genomically distinct clones. In a cytogenetically homogenous subclone, we identified two transitional tumour cell states and leveraged our single-nucleus spatial chromatin accessibility data to identify spatially autocorrelated transcription factor motifs likely to be participating in this mesenchymal-like transition.

Slide-tags offers several unique advantages as a spatial genomics technology. First, it is easily imported into frozen-tissue snRNA-seq experiments and enables the addition of spatially resolved data without requiring specialized equipment or sacrificing data quality. Second, the technique generates data intrinsically at the single-cell resolution, without the need for deconvolution and segmentation, and has a high sensitivity (2,000–10,000 UMIs per cell across our datasets). This substantially improves the ability to unbiasedly discover cell types and cell-type-specific gene expression in spatial data compared with pixel-based spatial transcriptomic technologies. Third, the technology is high-throughput, enabling many tissue sections to be profiled at once, and coverage of larger tissue sections through the construction of bigger bead arrays. Fourth, Slide-tags is easily adapted to many different single-cell and single-nucleus methodologies. Beyond our demonstration of spatial snRNA-seq + snATAC–seq, we envision that future adaptations of Slide-tags will enable the profiling of DNA^5,52^, additional epigenetic modifications^6,53,54^ and proteins^55^. Computational analyses of such data are uniquely enabled by the ability of Slide-tags to seamlessly leverage many existing single-cell computational workflows (for example, Seurat^21^, InferCNV^43^, ArchR^56^).

Although immediately useful in many applications, Slide-tags could be improved in two key ways. First, our method assays only a subset of nuclei in a tissue section. We estimate that the combination of dissociation and microfluidic losses during nuclei barcoding collectively account for around 75% of the nuclei lost. This loss reduces power in the discovery of pairwise interactions between cells, as well as molecular interactions between cells, which may be overcome through the scalability of Slide-tags profiling. This represents a path for substantial improvement through tissue-specific optimizations to the dissociation, and improved droplet microfluidics or, potentially, microfluidics-free single-nucleus methods that may barcode nuclei more efficiently^57^. Second, Slide-tags is currently limited to single-nucleus sequencing methods, primarily due to the ease of recovering nuclei from frozen tissues. Some methodologies strongly benefit from single-cell data, such as lineage tracing using mitochondrial genomic variants^58^ and quantification of transcriptional kinetics^59^. Future iterations of our technology may be compatible with tagging whole single cells. Nonetheless, for routine tissue profiling, our current default approach is snRNA-seq (versus scRNA-seq), owing to advantages in protocol flexibility, increased nucleus yields, reduced tissue dissociation artefacts and improvements to cell sampling bias^60^.

In recent years, a common experimental paradigm has evolved that pairs the collection of single-cell (or single-nucleus) data with spatial data to discover cell types, compare across conditions and discover spatial patterns within and across these types. Slide-tags represents a method to merge these experimental modalities into a unified approach, integrating the ascertainment of cytoarchitectural features with the standard collection of single-cell sequencing data. By importing the single-cell sequencing toolkit into the spatial repertoire, Slide-tags will serve as an invaluable tool to study tissue biology across organisms, ages and diseases.

## Methods

### Sample information and processing

#### Mouse brain

##### Mouse housing

Mice were group-housed under a 12 h–12 h light–dark schedule and allowed to acclimatize to their housing environment for 2 weeks after arrival. All of the procedures involving animals at the Broad Institute were conducted in accordance with the US National Institutes of Health Guide for the Care and Use of Laboratory Animals under protocol number 0120-09-16 and approved by the Broad Institutional Animal Care and Use Committee.

##### Brain preparation

At 56 days of age, male C57BL/6J mice were anaesthetized by administration of isoflurane in a gas chamber flowing 3% isoflurane for 1 min. Anaesthesia was confirmed by checking for a negative tail-pinch response. Animals were moved to a dissection tray and anaesthesia was prolonged through a nose cone flowing 3% isoflurane for the duration of the procedure. Transcardial perfusions were performed with ice-cold pH 7.4 HEPES buffer containing 110 mM NaCl, 10 mM HEPES, 25 mM glucose, 75 mM sucrose, 7.5 mM MgCl_2_ and 2.5 mM KCl to remove blood from the brain and other organs sampled. For use in regional tissue dissections, the brain was removed immediately and frozen for 3 min in liquid nitrogen vapour and then moved to −80 °C for long term storage.

Whole C57BL/6J mouse embryos at E14 (MF-104-14-Ser) were purchased from Zyagen and stored at −80 °C until use. A pregnant mouse was perfused with PBS before collection and snap-freezing of the whole embryo.

#### Human brain

Post-mortem autopsy tissue (Brodmann area 9 cortex) from a healthy, older, female, control individual was obtained from the University of Miami Brain Endowment Bank at the Miller School of Medicine. Tissue was collected in accordance with the standard patient informed consent procedures of the Brain Endowment Bank in effect at the time of collection and subject to approval or an exemption determination by their Institutional Review Board. Use of the tissue at the Broad Institute was approved by the Office of Research Subject Protection project NHSR-4235. This cortical sample was stored at −80 °C until use after equilibration at −20 °C in the cryostat. As a quality-control step, the tissue architecture was assessed by Nissl staining after frozen sectioning at 20 µm, and the RNA integrity was determined using TRIzol extraction followed by an RNA-integrity number (RIN) assay using the Agilent RNA nano 6000 Bioanalyzer method (RIN = 7.2).

#### Human tonsil

Anonymized excess tissue specimens were obtained from a patient who underwent a palatine tonsillectomy procedure for tonsillar enlargement. The specimens were embedded in OCT, snap-frozen and stored at −80 °C. As a quality-control step, the tissue architecture was assessed using H&E staining, and the RNA integrity was determined using the Tapestation RNA ScreenTape system (RIN^e^ > 7.5). The use of the tissue at the Broad Institute was approved by the Office of Research Subject Protection project IRB-6429.

#### Human metastatic melanoma

Samples were acquired from a patient who underwent axillary lymphadenectomy for metastatic *BRAF*-mutant melanoma before starting PD-1 inhibitor. The sample was embedded in OCT, snap-frozen after surgery and stored at −80 °C. The use of the tissue at the Broad Institute was approved by the Office of Research Subject Protection project NHSR-4182.

### Histological processing

For sections that were stained using Nissl, glass-mounted frozen tissue sections (10 or 20 µm) were equilibrated to room temperature and excess condensate was wiped off. Sections were fixed in 70% ethanol for 2 min, followed by rehydration in ultrapure water for 30 s. Excess water was wiped off and slides were stained with Arcturus Histogene Solution (Thermo Fisher Scientific, 12241-05) for 4 min. Excess dye was tapped off and the slides were rehydrated in water for 10 s for destaining. Slides were sequentially fixed in 70, 90 and 100% ethanol for 30 s, 10 s and 1 min, respectively, post-fixed in xylene solution for 1 min then mounted with Fisher Chemical Permount (SP15-100) and cover-slipped. Images were acquired using the Keyence BZ-800XE microscope under a Nikon Apo ×10 objective or the Leica Aperio VERSA Brightfield, Fluorescence & FISH Digital Pathology Scanner under a ×10 objective.

For sections that were stained using H&E, glass-mounted frozen tissue sections (10 or 20 µm) were equilibrated to room temperature and the excess condensate was wiped off. Sections were dipped in xylene, processed through a graded ethanol series and stained with haematoxylin. The nuclei were ‘blued’ by treatment with a weakly alkaline solution, and washed with water. Sections were stained with eosin, processed through a graded ethanol series, xylene, dehydrated and cover-slipped. Bright-field images were taken using the Leica Aperio VERSA Brightfield, Fluorescence & FISH Digital Pathology Scanner under a ×10 objective.

### Barcoded bead synthesis, array fabrication and sequencing

PLRP-S resin (1,000 Å, 10 μm; Agilent Technologies, PL1412-4102) was used for the barcoded oligonucleotide synthesis. The loading of the non-cleavable linker on resin was adjusted to approximately 30 µmol g^−1^. The Akta OligoPilot 10 oligonucleotide synthesizer was used for synthesis (850 mg scale). The PC linker (10-4920-90) and reverse phosphoramidites (10-0001, 10-9201, 10-0301 and 10-5101-10) were purchased from Glen Research. A 0.1 M solution of phosphoramidites was prepared in anhydrous acetonitrile, and 0.3 M BMT (BI0166-1005, Sigma-Aldrich) was used as an activator for coupling (single coupling, 6 min). Two capping steps (before and after oxidation) were performed with the cap A (BI0224-0505, Sigma-Aldrich) and cap B (B1:B2 1:1; BI0347-0505, BI0349-0505 Sigma-Aldrich) reagents. For the 6.3 ml column, capping was performed by 1 CV or 1.5 CV for 1 min; and, for the 1.2 ml column, 2 CV for 0.5 min. The oxidation (5 equiv) was performed with 0.05 M iodine in pyridine (BI0424-1005, Sigma-Aldrich). The detritylation step was performed using 3% dichloroacetic acid in toluene (BI0832-2505, Sigma-Aldrich).

After the oligonucleotide synthesis, the protecting groups were removed by incubating the resin in 40% aqueous methylamine for 24 h at room temperature (20 mg resin per 2 ml). The beads were washed twice with water (1 ml), three times with methanol (1 ml), three times with 1:1 acetonitrile:water and three times with acetonitrile (1 ml). Finally, the beads were washed three times with 10 mM Tris buffer pH 7.5 containing 0.01% Tween-20 and stored in the same buffer at 4 °C. It was observed that oligos were released in the buffer if the beads were stored for long periods of time. To remove the released oligos, beads were washed with 70% acetonitrile/water and resuspended in storage buffer.

Synthesized sequences for the Slide-tags experiments (PC in the sequences denote photocleavable linker) were as follows: (1) incorporation of capture sequence by ligation: the bold bases denote the region that is complementary to the sequence of the 10x gel beads (SLAC beads): 5′-TTT_PC_zCCGGTAATACGACTCACTATAGGGCTACACGACGCTCTTCCGATCTJJJJJJJJTCTTCAGCGTTCCCGAGAJJJJJJJNNNNNNNVVGCTCGGACACATGGGCG-3’, 10x FB1 extension: 5′-GAGCTTTGCTAACGGTCGAG**GCTTTAAGGCCGGTCCTAGCAA**-3′, splint: 3′-CTGTGTACCCGCCTCGAAACGATTGC-5′; (2) Direct synthesis of capture sequence on beads (TAGS beads): 5′-TTT-PC-GTGACTGGAGTTCAGACGTGTGCTCTTCCGATCTJJJJJJJJTCTTCAGCGTTCCCGAGAJJJJJJJNNNNNNNVV**GCTTTAAGGCCGGTCCTAGCAA**-3’; (3) poly(A) beads: 5′-TTT-PC-GTGACTGGAGTTCAGACGTGTGCTCTTCCGATCTJJJJJJJJTCTTCAGCGTTCCCGAGAJJJJJJJNNNNNNNVVA30.

Array preparation and sequencing were performed as described previously^20^.

### Slide-tags procedure

Fresh frozen tissues were cryo-sectioned to 20 μm on a Cryostat (CM1950, Leica) at −16 °C. Precooled 2 mm circular (3331P/25, Integra), 3 mm circular (3332P/25, Integra) or 5.5 mm square custom-made biopsy punches were used to isolate regions of interest from tissue sections. The punched tissue regions were then placed onto the puck, ensuring that there were no folds. A finger was placed onto the bottom of the puck to melt the tissue while trying to prevent rolling. Immediately, this puck was placed onto the glass slide and placed on ice, and 6–10 µl of dissociation buffer (82 mM Na_2_SO_4_, 30 mM K_2_SO_4_, 10 mM glucose, 10 mM HEPES, 5 mM MgCl_2_) was placed on top of the puck so that the buffer covered the whole puck. The puck was then placed under an ultraviolet (365 nm) light source (0.42 mW mm^−2^, Thorlabs, M365LP1-C5, Thorlabs, LEDD1B) for 30 s (TAGS beads) or 3 min (SLAC beads), to cleave the same amount of spatial barcode oligonucleotides between bead designs (Extended Data Fig. 2). After photo-cleavage, the puck was incubated for 7.5 min (TAGS beads) or 5 min (SLAC beads) and then placed into a 12-well plate (Corning, 3512). Using a 200 µl pipette, ten 200 μl aliquots of extraction buffer (dissociation buffer, 1% Kollidon VA64, 1% Triton X-100, 0.01% BSA, 666 U ml^−1^ RNase-inhibitor (Biosearch technologies, 30281-1)) were dispensed onto the puck for a total volume of 2 ml. Dispensed extraction buffer was triturated up and down on the puck 10–15 times to release the tissue. This step was repeated until the tissue was completely removed from the puck. The puck was removed, and mechanical dissociation of the supernatant was performed using 1 ml pipette 20–25 times trituration to fully dissociate the tissue. Dissociated nuclei were removed from the well and the well was rinsed twice with 1 ml of wash buffer (82 mM Na_2_SO_4_, 30 mM K_2_SO_4_, 10 mM glucose, 10 mM HEPES, 5 mM MgCl_2_, 50 µl of RNase-inhibitor (Biosearch technologies, 30281-1)), which was added to the nucleus suspension. Wash buffer was added to the tube to a final volume of 20 ml. This 20 ml was mixed and divided equally into another 50 ml falcon tube. Nuclei were centrifuged in a precooled swinging bucket centrifuge at 600*g* for 10 min at 4 °C. After centrifugation, 19.5 ml of the supernatant was removed, leaving 500 μl in each tube. The pellet was resuspended and pooled. This pooled suspension was then filtered using a precooled 40 µm cell strainer (Corning, 431750). DAPI (Thermo Fisher Scientific, 62248) was added to the filtered solution at a 1:1,000 dilution and incubated for 5–7 min at 4 °C. This was then centrifuged at 200*g* for 10 min at 4 °C. The supernatant was removed, leaving 50 μl of pellet. The pellet was resuspended and nuclei were counted manually using a C-Chip Fuchs-Rosenthal disposable haemocytometer (INCYTO, DHC-F01-5).

### Sequencing library preparation

#### snRNA-seq library preparation

For Slide-tags snRNA-seq experiments, 43.3 µl of counted nuclei was loaded into the 10x Genomics Chromium controller using the Chromium Next GEM Single Cell 3′ Kit v3.1 (10x Genomics, PN-1000268). The Chromium Next GEM Single Cell 3′ Reagent Kits v3.1 (Dual Index) with Feature Barcode Technology for Cell Surface Protein CG000317 was used according to the manufacturer’s recommendations with slight modifications. Spatial barcode libraries were prepared as cell-surface protein library preparations. The number of PCR cycles used for the index PCR step in the cell-surface protein library preparation (step 4.1f) for 5.5 × 5.5 mm TAGS arrays was 7; for 3 mm diameter TAGS arrays the number of cycles was 9.

For the mouse brain sample, ligated pucks (see sequence in the ‘Barcoded bead synthesis, array fabrication and sequencing’ section) were used for spatial barcoding. For this sample, a custom PCR protocol was used instead of step 4.1: 10 μl of cleaned supernatant from step 2.3, 50 µl NEBNext High-Fidelity 2× PCR Master Mix (NEB, M0541S), 2.5 µl STAG_P701_NEX (10 μM), 2.5 µl 10 μM P5-Truseq Hybrid oligo and 35 µl ultrapure DNase/RNase-free distilled water (Invitrogen, 10977015). In this sample, ten PCR cycles were performed according to the manufacturer’s recommendations.

#### snATAC-seq and snRNA-seq library preparation

For Slide-tags multiomic snATAC-seq and snRNA-seq experiments, 43.3 µl of counted nuclei was loaded into the 10x Genomics Chromium controller using the Chromium Next GEM Single Cell Multiome ATAC + Gene Expression Reagent Bundle (10x Genomics, PN-1000283). The Chromium Next GEM Single Cell Multiome ATAC + Gene Expression CG000338 Rev F user guide was used according to the manufacturer’s recommendations with slight modifications. During step 4.1, 1 μl of 0.329 μM spike-in primer (5′-GTGACTGGAGTTCAGACGT-3′) was added. For spatial barcode libraries, a custom PCR protocol was used: 5 μl of cleaned supernatant from step 4.3, 50 µl NEBNext High-Fidelity 2× PCR Master Mix (NEB, M0541S), 2.5 µl 10 μM STAG_iP7_a1 oligo (5′-CAAGCAGAAGACGGCATACGAGATATTTACCGCAGTGACTGGAGTTCAGACGT*G*T-3′), 2.5 µl 10 μM P5-STAG_ip5_a1 oligo (5′-AATGATACGGCGACCACCGAGATCTACACGACAATAAAGACACTCTTTCCCTACACGACGC*T*C-3′), 40 µl ultrapure DNase/RNase-free distilled water (Invitrogen, 10977015). In this sample, 15 PCR cycles were performed according to the protocol used in the Chromium Next GEM Single Cell 3′ Reagent Kits v3.1 (Dual Index) with Feature Barcode technology for Cell Surface Protein CG000317 Rev C user guide step 4.1.

#### TCR enrichment and library preparation

We enriched TCRs from Slide-tags multiome cDNA as previously described^44^ with the following modifications (https://www.protocols.io/view/slide-tcr-seq-v3-ivt-n92ldp6w8l5b/v2).

### Sequencing

We sequenced scRNA-seq and spatial barcode libraries on the Illumina NextSeq 1000 instrument using a p2 100 cycle kit (Illumina, 20046811). For some libraries, resequencing was performed to improve the sequencing depth, on an Illumina NovaSeq instrument using the S Prime platform.

### Slide-tags data preprocessing

#### snRNA-seq data

We used Cell Ranger (v.6.1.2)^1^ mkfastq (10x Genomics) to generate demultiplexed FASTQ files from the raw sequencing reads. We aligned these reads to either the human GRCh38 or mouse mm10 genome while including intronic reads with --include-introns, and quantified gene counts as UMIs using Cell Ranger count (10x Genomics). For mouse embryo, human brain, tonsil and melanoma, we used CellBender v.0.2.0 for background noise correction and cell calling^61^, setting --expected-cells to the number of Cell Ranger cell calls, --total-droplets-included to 40,000 and --learning-rate to 0.00005 (only when the default parameters were insufficient to produce cell probability calls of majority zero and one).

#### Multiomic snATAC-seq and snRNA-seq data

We used Cell Ranger-arc (v.2.0.2) mkfastq (10x Genomics) to generate demultiplexed FASTQ files from the raw sequencing reads. We aligned these reads to the human GRCh38 genome, and quantified gene counts as UMIs using Cell Ranger-arc count (10x Genomics). For the gene expression data, we then used CellBender for background noise correction and cell calling as described above.

#### Spatial barcode data

After creating demultiplexed FASTQ files, we searched using grep for reads containing the spatial barcode universal primer constant sequence. We then downsampled the spatial barcode-containing FASTQ file to 25 million reads using seqtk v.1.3-r106 for computational efficiency and consistency across runs. We then matched candidate cell barcodes in the spatial barcode FASTQ file with true cell barcodes outputted from either Cell Ranger v.6.1.2 or CellBender^61^ (Supplementary Table 14), generating a data frame of candidate spatial barcode sequences per true cell barcode. From this data frame, we matched candidate spatial barcode sequences with a whitelist of in situ sequenced spatial barcodes, assigning each true spatial barcode a spatial coordinate.

#### Assignment of spatial locations to nuclei

Slide-tags nuclei are assigned *x*,*y* coordinates corresponding to the distribution of spatial barcodes per nucleus (Supplementary Fig. 1). First, snRNA-seq or multiome data are preprocessed as described above to generate a gene-by-cell-barcode count matrix. The whitelist of cell barcodes from Cell Ranger and spatial barcodes from in situ bead array sequencing are matched in the spatial barcode FASTQ to generate a spatial-barcode-by-cell-barcode matrix. Spatial barcodes with outlier UMI counts (that is, UMI > 256) are removed as these probably represent beads dislodged from the glass slide during nucleus isolation and encapsulated in droplets with nuclei (data not shown). Then, taking the set of spatial barcodes and their *x*,*y* coordinates for each cell barcode, DBSCAN^62,63^ (v.1.1−11) is used to filter out noise spatial barcodes before spatial positioning of nuclei (Supplementary Fig. 1c). DBSCAN outputs a cluster assignment for each spatial barcode. Cluster = 0 corresponds to noise spatial barcodes without a clear spatial distribution, and cluster of numbers greater than zero correspond to signal spatial barcodes with discrete spatial clustering. We did not assign spatial positions to nuclei with all spatial barcodes denoted noise, or to nuclei with multiple signal clusters. From the remaining nuclei with one distinct spatial barcode signal cluster, we filtered out noise spatial barcodes and computed a UMI-weighted centroid of spatial barcode coordinates in the signal cluster. DBSCAN required two parameters as input: minPts and eps. To determine the optimal parameter set for each Slide-tags run, we iterated through minPts parameters from minPts = 3 to minPts = 15 under a constant eps = 50 and chose the parameter set with the highest proportion of nuclei that are assigned a spatial position (a single DBSCAN signal cluster). Sankey plots were generated using Sankeymatic (https://sankeymatic.com/).

#### TCR sequences

TCR sequences were identified using MiXCR (v.4.1.0)^64,65^ and assigned to cell barcodes using a hamming distance 1 collapse.

### Mouse brain analysis

#### Quality control and cell type assignment

The output generated by Cell Ranger was read into R (v.4.1.1) using Seurat (v.4.3.0)^21^. Filtering steps are quantified in Supplementary Fig. 1b. We normalized the total UMIs per nucleus to 10,000 (CP10K) and log-transformed these values to report gene expression as *E* = log[CP10K + 1]. We identified the top 2,000 highly variable genes after using variance-stabilizing transformation correction^66^. All gene expression values were scaled and centred. For visualization in two dimensions, we embedded nuclei in a UMAP^67^ using the top 30 principal components, with number of neighbours = 40, min_dist = 0.3, spread = 15, local connectivity = 12 and the cosine distance metric. We identified shared nearest neighbours using the top 30 principal components. Clusters of similar cells were detected using the Louvain method for community detection, implemented using FindClusters, with resolution = 0.8. Each cell was then assigned a predicted identity based on mapping to a mouse adult brain reference dataset^16^, using FindTransferAnchors and then TransferData, with the first 25 principal components in both cases. For each computed cell cluster, an identity was assigned using the highest proportion of transferred labels, and confirmed using known markers genes.

#### Assessment of spatial positioning accuracy

##### Spatial barcode metrics calculations

We measured the accuracy of spatial positioning for the 839 cell barcodes corresponding to high-quality mapped cells in our mouse hippocampus dataset (Fig. 1). For each of these cells, we used the spatial barcodes belonging to the DBSCAN singlet cluster and calculated the standard error for both *x* and *y* coordinates using:

$${\rm{s.e.}}=\frac{\sigma }{\sqrt{N}},$$

where *N* is the number of spatial barcode UMIs in the cluster, and *σ* is the s.d. of each of the spatial barcode UMIs from the centroid of the cluster.

In addition to the s.e., other metrics were calculated for each DBSCAN singlet cluster. Namely, the geometric mean distance of spatial barcodes from the centroid:

$${\underline{x}}_{{\rm{geom}}}={(\mathop{\prod }\limits_{i=1}^{n}| {x}_{i}-C| )}^{\frac{1}{n}},$$

where *n* is the number of spatial barcode UMIs in the cluster, and *x*_*i*_ − *C* is the absolute distance between each spatial barcode UMI and the cluster centroid.

For each cell that had only a single DBSCAN cluster, additional metrics were calculated (Extended Data Fig. 2d–g). The total number of unique spatial barcode sequences, and spatial barcode UMIs associated with each cell was calculated, regardless of whether it was in the singlet DBSCAN cluster or not. The ratio of spatial barcode UMIs within and outside the DBSCAN singlet cluster was then calculated as the proportion of signal spatial barcodes per cell.

##### CA1 width analysis

A serial section of the profiled region was stained using Nissl and imaged. Cells were segmented from this image using watershed segmentation in MATLAB (release 2021b) and the centroid of each segment was calculated. Next, these coordinates were read into R and DBSCAN was used to isolate cells belonging to the CA1 region, with the following parameters: eps = 35, minPts = 20. The image region was cropped to match that of the profiled Slide-tags region. For both datasets, a tenth-order (Nissl) or nineth-order (Slide-tags) linear model was fitted through these points, generating a central curve. For each spatial barcode UMI, the nearest neighbour on this curve in Euclidean space was determined and the distance from these two points was recorded as the distance from the fitted line.

##### CA1 sublayer analysis

Nuclei that belonged to the CA1 cluster were subsetted, and the top 1,000 highly variable genes in this subset of nuclei was identified after using variance-stabilizing transformation correction^66^. Principal component analysis (PCA) was performed using these variable genes. We identified shared nearest neighbours using the top 25 principal components. Clusters of similar cells were detected using the Louvain method for community detection, implemented using FindClusters, with resolution = 0.5. Differentially expressed genes between the two clusters were identified using FindMarkers with the default parameters. Sublayer labels were assigned to each cluster using previously identified gene expression markers^68,69^. In situ hybridization data for comparative plots were obtained from the Allen Mouse Brain Atlas^23^.

#### Comparison of Slide-tags snRNA-seq versus snRNA-seq data

For each sample, Cell Ranger was run as described above, and the outputs were run through Cell Ranger aggr (v6.1.2) to account for differences in the sequencing depth per cell. The result was a combined matrix of 25,158 nuclei, with 25,107 mean reads per cell, 2,309 median UMIs per cell and 1,438 median genes per cell. The filtered feature–barcode matrix generated by Cell Ranger was then read into R (v.4.1.1) using Seurat (v.4.3.0)^21^. We normalized the total UMIs per nucleus to 10,000 (CP10K) and log-transformed these values to report gene expression as *E* = log[CP10K + 1]. We identified the top 2,000 highly variable genes after using variance-stabilizing transformation correction^66^. All gene expression values were scaled and centred. For visualization in two dimensions, we embedded nuclei in a UMAP^67^ using the top 40 principal components, with number of neighbours = 40, min_dist = 0.3, spread = 15, local connectivity = 12 and the cosine distance metric. We identified shared nearest neighbours using the top 40 principal components. Clusters of similar cells were detected using the Louvain method for community detection, implemented using FindClusters, with resolution = 1. Each cell was then assigned a predicted identity based on mapping to a mouse adult brain reference dataset^16^ using FindTransferAnchors and then TransferData, with the first 25 principal components in both cases. These cell type designations were then used for comparative analysis going forward. Cells designated Unk_1 or Unk_2 were removed from the analysis as these cells showed low quality metrics and were not interpretable labels.

#### Comparison of Slide-tags snRNA-seq versus bulk RNA-seq

To compare the capture of both Slide-tags snRNA-seq and Slide-seq to bulk RNA-seq data, we used a bulk RNA-seq dataset from the mouse brain that we published previously^9^. To generate this dataset, the stranded mRNA Truseq kit (Illumina, 20020594) was used to prepare stranded poly(A) selection libraries from a dissected sagittal mouse hippocampus. The libraries were sequenced and transcripts per million (TPM) for each gene were generated using RSEM^70^ post-alignment with STAR^71^. For Slide-seq data, we used two previously published datasets: Slide-seqV1 (ref. ^9^) Puck_180819_6 and Slide-seqV2 (ref. ^20^) Puck_200115_08. The average TPM (APTM) was computed by summing counts for each gene across all beads on a puck and dividing by the sum of all UMIs on the puck, and dividing by 1 million (total UMI counts/1 million). For Slide-tags snRNA-seq data, to make an appropriate comparison, data were quantified to exclude intronic reads. The APTM was then computed by summing counts for each gene across all nuclei on the puck used in Fig. 1g–i, and dividing by the sum of all UMIs across all nuclei, and dividing by 1 million (total UMI counts/1 million). The per-gene distribution for each of these values (bulk TPM and Slide-seq ATPM) was plotted and linear regression was performed to calculate the Pearson’s correlation coefficient.

#### Comparison of Slide-tags snRNA-seq with Slide-seqV2 and DBiT-seq

Slide-tags snRNA-seq mouse hippocampus data were compared with Slide-seqV2 (ref. ^20^) mouse brain data and DBiT-seq mouse brain data (Spatial-ATAC-RNA-seq^13^). For gene and UMI count comparisons, Slide-seq data were spatially binned to 20 μm spatial square pixels. Slide-tags snRNA-seq data were processed and nuclei were embedded in UMAP space as described above. Slide-seqV2 and DBiT-seq total UMIs per spatial spot (10 μm beads in Slide-seqV2) were normalized to 10,000 (CP10K) and log-transformed to report gene expression as *E* = log[CP10K + 1]. The top 2,000 highly variable genes were identified after using variance-stabilizing transformation correction^66^. Gene expression values were scaled and centred. For visualization in two dimensions, we embedded spatial spots in UMAP space using the top 30 principal components, with number of neighbours =30, min_dist = 0.3, spread =1, local connectivity = 1 and the cosine distance metric. We identified shared nearest neighbours using the top 30 principal components. For Slide-seqV2, clusters of similar cells were detected using the Louvain method for community detection, implemented using FindClusters, with resolution = 1. RNA clusters from the Spatial-ATAC–RNA-seq publication^13^ were used for DBiT-seq data. Standard deviations for the top 30 principal components were plotted using ElbowPlot in Seurat. Dot plots display the SCTransformed expression values for DBiT-seq from the Spatial-ATAC–RNA-seq publication.

### Mouse embryonic brain at E14 analysis

The output generated by Cell Ranger was read into R (v.4.1.1) using Seurat (v.4.3.0)^21^. We normalized the total UMIs per nucleus to 10,000 (CP10K) and log-transformed these values to report gene expression as *E* = log[CP10K + 1]. We identified the top 2,000 highly variable genes after using variance-stabilizing transformation correction^66^. All gene expression values were scaled and centred. For visualization in two dimensions, we embedded nuclei in a UMAP^67^ using the top 30 principal components, with number of neighbours = 40, min_dist = 0.3, spread = 15, local connectivity  = 12 and the cosine distance metric. We identified shared nearest neighbours using the top 30 principal components. Clusters of similar cells were detected using the Louvain method for community detection, implemented using FindClusters, with resolution = 0.8. Each cell was then assigned a predicted identity based on mapping to a mouse embryo at E14 reference dataset^18^, using FindTransferAnchors and then TransferData, with the first 25 principal components in both cases. For each computed cell cluster, an identity was assigned using the highest proportion of transferred labels, and confirmed using known marker genes.

### Human brain analysis

#### Quality control and cell type assignment

The output generated by Cell Ranger was filtered by CellBender and read into R (v.4.2.2). The matrix was subsetted down to cells that had exactly one DBSCAN location and fewer than 5% mitochondrial UMIs, which were then loaded into Seurat (v.4.3.0)^21^ to perform normalization, finding variable features, scaling, PCA, finding neighbours (dims=30) and finding clusters, and to create a UMAP, all with the default parameters (unless otherwise specified). Each cluster was assigned a cell class (excitatory neuron, inhibitory neuron, oligodendrocyte, OPC, Astroce, endothelial cell, microglia) by plotting canonical cell type marker genes on the UMAP and manually assigning each cluster a cell type. Subsequently, excitatory and inhibitory neuron subtypes were mapped from a published human cortex dataset^25^ by label transfer using Harmony v.0.1.1 and spatially plotted in Supplementary Figs. 2b and 3b.

#### Identification of layers and layer-dependent gene expression

The layer assignment of each cell (L1–2, L3–5, L6, WM) was calculated by manually drawing boundaries between the layer-specific mapped neuron subtypes and assigning each cell a label depending on which two boundaries it was between. The numerical laminar coordinate was then calculated by taking the Euclidean distance of each cell to the nearest boundary and dividing it by the sum of the distances to the two neighbouring boundaries, adding a constant factor depending on the layer assignment.

Before computing the spatial variation score for each gene, nuclei were removed if they contained expression above a Z-score of 2 for a marker gene of a different cell type. Subsequently, each gene was assigned a spatial variation score by computing the kernelized density of the gene expression along the laminar coordinate of filtered cells using a uniform kernel and taking the difference between the highest and lowest expression density values (Supplementary Table 2). Complex gradients were found by taking the intersection of each cell type’s spatially variable gene list, and a visually selected interesting subset is shown in Fig. 2l.

Gene Ontology analysis was performed on all genes with a spatial variation *Z* score above 7.0 using EnrichGO from clusterProfiler v.4.6.0 (using the default parameters) and using annotations from org.Hs.eg.db v.3.16.0 (Supplementary Table 3) under the biological process ontology. For display in Fig. 2k, the terms were further subsetted to include only terms with *P*_adj_ < 1 × 10^−8^ in at least one cell type.

Genes with a spatial variation *Z* score above 10 in excitatory/inhibitory neurons and above 8 in astrocytes/OPCs are shown in the heat maps in Fig. 2i,j and Extended Data Fig. 6d,e. Genes that additionally had a minimum expression below 0.8 were spatially plotted in Supplementary Fig. 4–6.

#### Reproducibility analysis

The percentage of high-quality nuclei that were spatially positioned and the density of mapped nuclei were compared across four human cortex Slide-tags runs (Supplementary Table 4). For each run, the cell calls generated as output by Cell Ranger were used and low-quality cells were removed if they belonged to a cluster with an average mitochondrial nUMIs percentage of greater than 5%. Then, the percentage of mapped nuclei was computed by dividing the number of nuclei with exactly one DBSCAN location by the total number of nuclei. The nucleus density was calculated by selecting a window of tissue with equal white and grey matter area and dividing the number of spatially positioned nuclei in the window by the window area.

### Tonsil analysis

#### Quality control and cell type assignment

The output generated by Cell Ranger and filtered by CellBender was read into R (v.4.1.1) using Seurat (v.4.3.0)^21^. We normalized the total UMIs per nucleus to 10,000 (CP10K) and log-transformed these values to report gene expression as *E* = log[CP10K + 1]. We identified the top 2,000 highly variable genes after using variance-stabilizing transformation correction^66^. All gene expression values were scaled and centred. For visualization in two dimensions, we embedded nuclei in a UMAP^67^ using the top 30 principal components, with number of neighbours =30, min_dist = 0.3, spread =1, local connectivity = 1 and the cosine distance metric. We identified shared nearest neighbours using the top 30 principal components. Clusters of similar cells were detected using the Louvain method for community detection, implemented using FindClusters, with resolution = 1. Annotation of de novo clusters was aided by marker genes and Azumith^21^ reference-based mapping from the human tonsil atlas^72^.

#### Spatially varying gene expression

Significantly non-random genes were discovered in GCB cells as described previously^9^. In brief, for each single-nucleus assigned as a germinal centre B cell that was positioned in one of the four largest germinal centres that we profiled, we first calculated the matrix of pairwise Euclidean distances between cells for each germinal centre individually. We then compared the distribution of pairwise distances between the cells expressing at least one count of that transcript to the distribution of pairwise distances between an identical number of cells, sampled randomly from all mapped beads within the set with probability proportional to the total number of UMIs per cell. Specifically, we generated 1,000 such random samples, and for each sample calculated the distribution of pairwise distances. We then calculated the average distribution of pairwise distances, averaged across all 1,000 samples. Finally, we calculated the L1 norm between the distribution of pairwise distances for the true sample of cells and the average distribution. We defined *p* to be the fraction of random samples with distributions closer to the average distribution (under the L1 norm) than the true sample. We calculated an *Z* score for the true sample given the distribution distances from the average distribution of random samples. Finally, we aggregated *p* values for spatial variation from each of the four tested germinal centres using Fisher’s method.

We intersected our computed spatially varying genes with genes that were previously implicated in germinal centre zone distinction^73^. We calculated the percentage variance in gene expression space and plotted it against the spatial effect size from our spatial permutation test to identify genes with relatively low gene expression variance but high spatial variance.

#### Germinal-centre zonation

We used spatially varying genes (*P* < 0.05) identified as described above to classify GCB cells into light-zone, dark-zone and transitional states. Specifically, we subsetted our data to GCB cells, rescaled and recentred values, and ran PCA on the 1,068 significant spatially varying genes. We then identified shared nearest neighbours using the top 15 principal components. Clusters of similar cells were detected using the Louvain method for community detection, implemented using FindClusters, with resolution = 0.4. We annotated clusters as light-zone, dark-zone and transitional states using marker genes and Azumith^21^ reference-based mapping from the human tonsil atlas^72^.

After classifying GCB cells into states, we spatially segmented germinal centres into light zones and dark zones using dark-zone B cell spatial density. We ran DBSCAN^62^ on dark-zone B cells of the two largest germinal centres, using eps = 60 and minPts = 6 for the largest germinal centre, and eps = 60 and minPts = 10 for the second-largest germinal centre. We considered cells within the top DBSCAN cluster to constitute the dark zone and segmented around the outer cells. The remaining cells in both germinal centres were considered to be in the light zone and segmentation borders were drawn accordingly. We tested for zone bias of T follicular helper cells and follicular dendritic cells using chisq.test from the stats package in R (v.4.2.2).

#### Spatial receptor–ligand prediction

To detect receptor–ligand interactions between cell type pairs, we computed a receptor–ligand score based on a spatial correlation index^74^, SCI, which we defined as:

$${\rm{SCI}}=\frac{\mathop{\sum }\limits_{i}^{N}\mathop{\sum }\limits_{j}^{M}{w}_{ij}{r}_{i}{l}_{j}}{\mathop{\sum }\limits_{i}^{N}\mathop{\sum }\limits_{j}^{M}{w}_{ij}}$$

between *N* cells of ‘sender cell type’ expressing receptor *r* and *M* cells of ‘receiver cell type’ expressing ligand *l*, where expression is sctransform counts^75^. We defined the spatial weights matrix of dimensionality *N* × *M* as an adjacency matrix, denoting 1 for when sender cell *i* is within 100 μm of receiver cell *j* and 0 otherwise. We first ran LIANA^33^ (v.0.1.12) to generate a putative list of receptor–ligand interactions between cell type pairs in a spatial agnostic manner, filtering to receptor–ligand interactions that are expressed in at least 50 cells of sender and receiver cell types (log[CPM] > 0), or in 30% of sender and receiver cells. We then computed a spatial correlation index for each receptor–ligand interaction to determine whether the receptor and ligand are spatially co-expressed in a given cell type pair.

To determine the spatial significance of a receptor–ligand score, we used an adaptive spatial permutation test, running 1,000 permutations for each receptor–ligand interaction. In each permutation, we randomly permuted the spatial locations of cells within a given cell-type. For interactions with a nominal *P* value less than or equal to 0.005, we ran an additional 9,000 permutations. We corrected for multiple-hypothesis testing using the Benjamini–Hochberg procedure. We also computed the log-transformed FC between the observed SCI statistic and the median SCI statistic of the empirical null distribution. This enabled us to compare SCI log-transformed FC values between receptor–ligand interactions for different cell types without explicitly correcting for the number of cells of each cell type.

#### Spatial contextualization of receptor–ligand interactions

To spatially contextualize receptor–ligand interactions, we decomposed spatial correlation indices for each significant interaction between GCB cells, T follicular helper cells and follicular dendritic cells (*P*_adj_ < 0.05) into interaction intensity scores for individual cells^76^. These decomposed scores reflect each individual cell’s contribution to the total spatial correlation index, defined as follows for receiving cell *i* and vice versa for sender cell *j*:

$${\rm{LISA}}=\frac{{r}_{i}\mathop{\sum }\limits_{j}^{M}{w}_{ij}{l}_{j}}{\mathop{\sum }\limits_{j}^{M}{w}_{ij}}$$

comparing interaction intensity scores of the receptor of each cell between dark zones and light zones. We corrected *P* values using the Benjamini–Hochberg method. Zone-specific receptor expression was tested using SCTransformed expression values compared between dark zones and light zones also using wilcox.test in R.

### Melanoma analysis

#### Quality control and cell type assignment

##### snRNA-seq data

The Cell Ranger output was filtered by CellBender and read into R (v.4.1.1) using Seurat (v.4.3.0)^21^. We normalized the total UMIs per nucleus to 10,000 (CP10K) and log-transformed these values to report gene expression as *E* = log[CP10K + 1]. We identified the top 2,000 highly variable genes after using variance-stabilizing transformation correction^66^. All gene expression values were scaled and centred. For visualization in two dimensions, we embedded nuclei in a UMAP^67^ using the top 30 principal components, with number of neighbours =30, min_dist = 0.3, spread =1, local connectivity = 1 and the cosine distance metric. We identified shared nearest neighbours using the top 30 principal components. Clusters of similar cells were detected using the Louvain method for community detection, implemented using FindClusters, with resolution = 1. Annotation of de novo clusters was aided by marker genes.

##### Multiome ATAC and snRNA-seq data

The RNA expression matrix generated by Cell Ranger was read into R (v.4.1.1) using Seurat^21^. The ATAC-filtered feature-barcode matrix generated by Cell Ranger was read into R (v.4.1.1) using Signac (v.1.9.0)^77^, and added as its own assay slot in the Seurat object containing RNA expression counts. Peaks were recalled using the CallPeaks function, which uses MACS2 (v.2.2.7.1)^78^, across all cells. Fragments were mapped to the MACS2-called peaks and assigned to nuclei using the FeatureMatrix function in Signac. Peaks in non-standard chromosomes were removed using keepStandardChromosomes from GenomeInfoDb (v.1.35.15)^79^ and problematic regions of the hg38 genome were removed using subsetByOverlaps according to the blacklist available at GitHub (https://github.com/Boyle-Lab/Blacklist)^80^. This final peaks–barcode matrix was then added to the ‘peaks’ assay within the Seurat object.

For cell type annotation, the snRNA-seq data from the multiome experiment were normalized for the total UMIs per nucleus to 10,000 (CP10K) and log-transformed to report gene expression as *E* = log[CP10K + 1]. The top 2,000 highly variable genes were identified after using variance-stabilizing transformation correction^66^. We then integrated the gene expression data from Slide-tags multiome with gene expression data from Slide-tags snRNA-seq using SelectIntegrationFeatures, FindIntegrationAnchors and IntegrateData across all features with the default parameters of Seurat (v.4.3.0). Integrated gene expression values were scaled and centred. For visualization in two dimensions, we embedded nuclei in a UMAP^67^ using the top 30 principal components, with number of neighbours =30, min_dist = 0.3, spread =1, local connectivity = 1 and the cosine distance metric. We identified shared nearest neighbours using the top 30 principal components. Clusters of similar cells were detected using the Louvain method for community detection, implemented using FindClusters, with resolution = 1. Cells from Slide-tags multiome were annotated based on marker genes and co-clustering with Slide-tags snRNA-seq cells. Gene expression counts from Slide-tags multiome were rescaled and reclustered as described above using the non-integrated object for subsequent analyses.

#### Inferring CNV

InferCNV (v.1.3.3) was used to infer large-scale CNVs from standard snRNA-seq data and from snRNA-seq data from a 10x multiome experiment as previously recommended (inferCNV of the Trinity CTAT Project; https://github.com/broadinstitute/inferCNV). CellBender-corrected counts were extracted from annotated Seurat objects, where normal reference cells were specified as all cells that were not labelled as tumour. InferCNV was run under the following parameters: cutoff = 0.1, cluster_by_groups = T, denoise = T, HMM = T, num_threads = 60.

#### TCR analysis

TCR analyses focused on CD8^+^ T cells; we used Fisher’s exact test to test whether (1) the β-chain sequence CASRASNEQFF was tumour-compartment biased compared against all CD8^+^ T cells with profiled β-chains, where tumour compartment segmentation was performed manually based on tumour subpopulation density; and (2) paired CD8^+^ T cells with TCR α-chain CAEWYNQGGKLIF and β-chain CASRASNEQFF were tumour-compartment biased.

#### ATAC analysis

Latent semantic indexing (LSI) was performed on the peaks assay using Signac, with the RunTFIDF and RunSVD functions. For visualization in two dimensions, we embedded nuclei in a UMAP^67^ using LSI dimensions 2–30. Nuclei were visualized using the combination of modalities profiled, with weighted-nearest neighbour analysis. Multimodal neighbours were identified using Seurat’s FindMultiModalNeighbors function, with the RNA PCA dimensions 1:50, and the ATAC LSI dimensions 2:50. These neighbours were then used as an input into RunUMAP for visualization.

To annotate the motifs present in peaks, the Signac function CreateMotifObject was used to create a motif object, with all human motifs from the Jaspar 2020 database. Motif accessibility *Z* scores were then calculated using Signac’s RunChromVAR function (chromVAR v.1.16.0). Gene activity scores were calculated using the Signac function GeneActivity. We normalized these gene scores by normalizing the total gene score per nucleus to the median nUMI for the RNA assay (NGS) and log-transformed these values to report gene expression as *E* = log[NGS + 1].

#### Differential gene expression, differential chromatin gene scores and gene set enrichment analysis

Differential gene expression analyses were performed using the MAST implemented in FindMarkers from Seurat^81^. Analysis comparing tumour cluster 1 and tumour cluster 2 from Slide-tags snRNA-seq and comparing compartment-specific CD8^+^ T cells from Slide-tags multiome data used min.pct = 0.25 and log2fc.threshold = 0.25. Analysis comparing tumour cluster 1 and tumour cluster 2 from Slide-tags multiome data used min.pct = 0.1 and log2fc.threshold = 0.25. Gene Ontology biological process (GO_Biological_Process_2021) gene set enrichment analysis was performed using the Enrichr package (v.3.1) in R^82–84^ on tumour cluster 2 enriched differentially expressed genes with log_2_[FC] < −0.5 and *P*_adj_ < 0.05. Differential chromatin gene score analysis was conducted using the Wilcoxon rank-sum test implemented in FindMarkers from Seurat with min.pct = 0.1 and log2fc.threshold = 0.

#### Melanocytic-like and mesenchymal-like signatures

We scored tumour cells on melanocytic-like and mesenchymal-like signatures using AddModuleScore in Seurat with a list of genes adapted from previous work^46,85^ (Supplementary Table 15). Correlations of chromVAR motif scores with mesenchymal scores were tested using Pearson’s correlation coefficient and *P* values were corrected using the Benjamini–Hochberg procedure. Spatial autocorrelations of chromVAR motifs were tested using Moran.I from the ape package (v.5.6-2) in R^86^, where the weights matrix was specified as 1/distance^2^.

### Reporting summary

Further information on research design is available in the Nature Portfolio Reporting Summary linked to this article.

## Online content

Any methods, additional references, Nature Portfolio reporting summaries, source data, extended data, supplementary information, acknowledgements, peer review information; details of author contributions and competing interests; and statements of data and code availability are available at 10.1038/s41586-023-06837-4.

### Supplementary information

Supplementary Information Supplementary Methods 1–7 and Supplementary References.

Reporting Summary

Supplementary Figures Supplementary Figs. 1–8.

Supplementary Table 1 CA1 sublayer differential expression analysis results.

Supplementary Table 2 Per-cell-type gene list ranked by spatial variation, human cortex.

Supplementary Table 3 GO enrichment analysis results, human cortex.

Supplementary Table 4 Summary statistics for replicate Slide-tags snRNA-seq runs in the human cortex.

Supplementary Table 5 Spatial varying genes in germinal centres, human tonsil.

Supplementary Table 6 Spatial effect size from spatial varying gene expression results and percentage variance in gene expression, human tonsil.

Supplementary Table 7 Receptor–ligand interaction prediction results, human tonsil.

Supplementary Table 8 Germinal centre zone enrichment test of receptor–ligand interactions, human tonsil.

Supplementary Table 9 Compartment-specific TCR sequences of CD8 T cells, human melanoma.

Supplementary Table 10 Differential gene expression between CD8 T cells in tumour compartment 1 and tumour compartment 2 from Slide-tags multiome performed on human melanoma.

Supplementary Table 11 Differential gene expression between tumour cluster 1 and tumour cluster 2 from Slide-tags snRNA-seq performed on human melanoma.

Supplementary Table 12 Differential gene expression (*x*) and differential chromatin gene scores (*y*) between tumour cluster 1 and tumour cluster 2 from Slide-tags multiome performed on human melanoma.

Supplementary Table 13 Spatial autocorrelation of chromVAR transcription factor motifs correlated with mesenchymal-like scores in tumour cluster 1, human melanoma.

Supplementary Table 14 Metadata and data preprocessing details for each Slide-tags experiment.

Supplementary Table 15 Mesenchymal-like and melanocytic-like genes used to score tumour cells, human melanoma.

## Data Availability

Slide-tags datasets have been deposited at the Broad Institute Single Cell Portal under the following accession numbers: SCP2162 (mouse brain), SCP2170 (mouse embryonic brain), SCP2167 (human brain), SCP2169 (human tonsil), SCP2171 (human melanoma) and SCP2176 (human melanoma multiome). Raw and processed mouse data have been deposited at the Gene Expression Omnibus under accession number GSE244355.

## Code availability

Code for processing spatial sequencing libraries is available at GitHub (https://github.com/broadchenf/Slide-tags (10.5281/zenodo.2571615).
