# Publisher Correction: Slide-tags enables single-nucleus barcoding for multimodal spatial genomics

**DOI:** 10.1038/s41586-023-06961-1

**Published:** 2023-12-18

**Authors:** Andrew J. C. Russell, Jackson A. Weir, Naeem M. Nadaf, Matthew Shabet, Vipin Kumar, Sandeep Kambhampati, Ruth Raichur, Giovanni J. Marrero, Sophia Liu, Karol S. Balderrama, Charles R. Vanderburg, Vignesh Shanmugam, Luyi Tian, J. Bryan Iorgulescu, Charles H. Yoon, Catherine J. Wu, Evan Z. Macosko, Fei Chen

**Affiliations:** 1 Broad Institute of Harvard and MIT, Cambridge, MA USA; 2 Department of Stem Cell and Regenerative Biology, Harvard University, Cambridge, MA USA; 3 Biological and Biomedical Sciences Program, Harvard University, Cambridge, MA USA; 4 Department of Biomedical Informatics, Harvard University, Boston, MA USA; 5 Biophysics Program, Harvard University, Boston, MA USA; 6 Harvard-MIT Division of Health Sciences and Technology, Massachusetts Institute of Technology, Cambridge, MA USA; 7 Department of Pathology, Brigham and Women’s Hospital, Harvard Medical School, Boston, MA USA; 8 Department of Medical Oncology, Dana-Farber Cancer Institute, Boston, MA USA; 9 Department of Medicine, Brigham and Women’s Hospital, Harvard Medical School, Boston, MA USA; 10 Division of Stem Cell Transplantation and Cellular Therapies, Dana-Farber Cancer Institute, Boston, MA USA; 11 Department of Surgical Oncology, Brigham and Women’s Hospital, Harvard Medical School, Boston, MA USA; 12 Department of Psychiatry, Massachusetts General Hospital, Boston, MA USA; 13 Present Address: Guangzhou Laboratory, Guangdong, China; 14 Present Address: Molecular Diagnostics Laboratory, Department of Hematopathology, Division of Pathology and Laboratory Medicine, The University of Texas MD Anderson Cancer Center, Houston, TX USA

**Keywords:** Genomics, Gene expression analysis, Epigenetics analysis

Correction to: *Nature* 10.1038/s41586-023-06837-4 Published online 13 December 2023

This article was originally published under a standard Springer Nature licence (© The Author(s), under exclusive licence to Springer Nature Limited). It is now available as an open-access paper under a Creative Commons Attribution 4.0 International licence, © The Author(s). The error has been corrected in the HTML and PDF versions of the article.

