# Supplementary material for: Slide-tags enables single-nucleus barcoding for multimodal spatial genomics
Source: Nature. 2023 Dec 13;625(7993):101–9. doi: 10.1038/s41586-023-06837-4 (PMC10764288; doi:10.1038/s41586-023-06837-4)
Supplement: Supplementary file 1 — Supplementary Methods 1–7 and Supplementary References. [file 41586_2023_6837_MOESM1_ESM.docx]

Materials and Methods

1 - Experimental methods

1.1 - Sample information and processing

1.2 - Histological processing

1.3 - Spatial barcoding of bead arrays

1.4 - Slide-tags procedure

1.5 - Sequencing library preparation

1.6 - Sequencing

2 - Data preprocessing

2.1 - snRNA-seq data

2.2 - Multiomic snATAC and snRNA-seq data

2.3 - Spatial barcode data

2.4 - Assignment of spatial locations to nuclei

2.5 - TCR sequences

3 - Mouse brain analysis

3.1 - Quality control and cell type assignment

3.2 - Assessment of spatial positioning accuracy

3.3 - Comparison of Slide-tags snRNA-seq vs. snRNA-seq data

3.4 - Comparison of Slide-tags snRNA-seq vs. bulk RNA-seq

3.5 - Comparison of Slide-tags snRNA-seq with Slide-seqV2 and DBiT-seq

4 - Mouse embryonic brain at E14

4.1 - Quality control and cell type assignment

5 - Human brain analysis

5.1 - Quality control and cell type assignment

5.2 -  Identification of layers and layer-dependent gene expression

6 - Human tonsil analysis

6.1 - Quality control and cell type assignment

6.2 - Spatial varying gene expression

6.3 - Germinal centre zonation

` 6.4 - Spatial receptor-ligand prediction

6.5 - Spatial contextualization of receptor-ligand interactions

7 - Human metastatic melanoma analysis

7.1 - Quality control and cell type assignment

7.2 - Inferring copy number variation

7.3 - T cell receptor analysis

7.4 - ATAC analysis

7.5 - Differential gene expression, differential chromatin gene scores, and gene set enrichment analysis

7.6 - Melanocytic-like and mesenchymal-like signatures

**1 - Experimental methods**

**1.1 Sample information and processing**

*Mouse brain*

*Animal housing.* Animals were group-housed with a 12-hour light-dark schedule and allowed to acclimate to their housing environment for two weeks post arrival. All procedures involving animals at the Broad Institute were conducted in accordance with the US National Institutes of Health Guide for the Care and Use of Laboratory Animals under protocol number 0120-09-16 and approved by the Broad Institutional Animal Care and Use Committee.

*Brain preparation.* At 56 days of age, C57BL/6J mice were anaesthetised by administration of isoflurane in a gas chamber flowing 3% isoflurane for 1 minute. Anaesthesia was confirmed by checking for a negative tail pinch response. Animals were moved to a dissection tray and anaesthesia was prolonged via a nose cone flowing 3% isoflurane for the duration of the procedure. Transcardial perfusions were performed with ice cold pH 7.4 HEPES buffer containing 110 mM NaCl, 10 mM HEPES, 25 mM glucose, 75 mM sucrose, 7.5 mM MgCl_2_, and 2.5 mM KCl to remove blood from brain and other organs sampled. For use in regional tissue dissections, the brain was removed immediately and frozen for 3 minutes in liquid nitrogen vapour and then moved to -80 ^o^C for long term storage.

Whole C57 mouse embryos at E14 (MF-104-14-Ser) were purchased from Zyagen and stored at −80 °C until use. A pregnant mouse was perfused with PBS prior to harvesting and snap freezing of the whole embryo.

*Human brain*. Postmortem autopsy tissue (Brodmann area 9 cortex) from a healthy, aged, female, control case was obtained from the University of Miami Brain Endowment Bank at the Miller School of Medicine. Tissue was collected in accordance with the standard patient informed consent procedures of the Brain Endowment Bank in effect at the time of collection and subject to approval or an exemption determination by their Institutional Review Board.  Use of the tissue at the Broad Institute was approved by the Office of Research Subject Protection project NHSR-4235. This cortical specimen was stored at -80 °C until use following equilibration at -20 °C in the cryostat. As a quality control step, tissue architecture was assessed by Nissl staining following frozen sectioning at 20 µm, and RNA integrity was determined using trizol extraction followed by RIN assay via the Agilent RNA nano 6000 bioanalyzer method (RIN = 7.2).

*Human tonsil*. Anonymized excess tissue specimens were obtained from a patient who underwent a palatine tonsillectomy procedure for tonsillar enlargement. The specimens were embedded in OCT, snap-frozen and stored at -80°C. As a quality control step, tissue architecture was assessed by hematoxylin and eosin staining, and RNA integrity was determined using the Tapestation RNA ScreenTape system (RIN^e^ > 7.5). Use of the tissue at the Broad Institute was approved by the Office of Research Subject Protection project IRB-6429.

*Human metastatic melanoma*. Specimens were acquired from a patient who underwent axillary lymphadenectomy for metastatic BRAF-mutant melanoma prior to starting PD-1 inhibitor. The specimen was embedded in OCT, snap frozen following surgery, and stored at -80 °C. Use of the tissue at the Broad Institute was approved by the Office of Research Subject Protection project NHSR-4182.

**1.2 Histological processing**

For sections that were stained using Nissl, glass-mounted frozen tissue sections (10 or 20 µm) were equilibrated to RT and excess condensate was wiped off. Sections were fixed in 70% ethanol for 2  min, followed by rehydration in ultrapure water for 30  s. Excess water was wiped off and slides were stained with Arcturus Histogene Solution (ThermoFisher, no. 12241-05) for 4  min. Excess dye was tapped off and slides were rehydrated in water for 10  s for destaining. Slides were sequentially fixed in 70, 90 and 100 % ethanol for 30  s, 10  s and 1  min, respectively, post-fixed in xylene solution for 1  min then mounted with Fisher Chemical Permount (no. SP15-100) and coverslipped. Images were acquired with a Keyence BZ-800XE microscope under a Nikon Apo 10x  objective or the the Leica Aperio VERSA Brightfield, Fluorescence & FISH Digital Pathology Scanner under a 10x objective.

For sections that were stained using hematoxylin and eosin H&E, glass-mounted frozen tissue sections (10 or 20 µm) were equilibrated to RT and excess condensate was wiped off. Sections were dipped in xylene, processed through a graded ethanol series, and stained with hematoxylin. The nuclei were “blued” by treatment with a weakly alkaline solution, and washed with water. Sections were stained with eosin, processed through a graded ethanol series, xylene, dehydrated, and coverslipped. Brightfield images were taken using the Leica Aperio VERSA Brightfield, Fluorescence & FISH Digital Pathology Scanner under a 10x objective.

**1.3 Barcoded bead synthesis, array fabrication, and sequencing**

PLRP-S resin (1000 A, 10-μm; Agilent Technologies, PL1412-4102) was used for the barcoded oligonucleotide synthesis. The loading of the non-cleavable linker on resin was adjusted to approximately 30 µmol/g. The Akta OligoPilot 10 oligonucleotide synthesizer was used for synthesis (850 mg scale). The PC linker (cat. no. 10-4920-90) and reverse phosphoramidites (10-0001, 10-9201, 10-0301, and 10-5101-10) were purchased from Glen Research. A 0.1 M solution of phosphoramidites was prepared in anhydrous acetonitrile (ACN) and 0.3 M BMT (BI0166-1005, Sigma-Aldrich) was used as an activator for coupling (single coupling, 6 min). Two capping steps (before and after oxidation) were performed with Cap A (BI0224-0505, Sigma-Aldrich) and Cap B (B1:B2 1:1; BI0347-0505, BI0349-0505 Sigma-Aldrich) reagents. For the 6.3 mL column, capping was performed by 1 CV or 1.5 CV with one min and for 1.2 mL column, 2 CV for 0.5 min. The oxidation (5 equiv) was carried out with 0.05 M iodine in pyridine (BI0424-1005, Sigma-Aldrich). The detritylation step was performed using 3% dichloroacetic acid in toluene (BI0832-2505, Sigma-Aldrich).

After the oligonucleotide synthesis, the protecting groups were removed by incubating the resin in 40% aqueous methylamine for 24 hr at room temperature (20 mg resin/ 2mL). The beads were washed twice with water (1 mL), three times with methanol (1 mL), three times with 1:1 acetonitrile: water, and three times with acetonitrile (1 mL). Finally, beads were washed three times with 10 mM Tris buffer pH 7.5 containing 0.01% tween-20 and stored in the same buffer at 4 °C. It was observed that oligos were released in the buffer if the beads were stored for long periods of time. In order to remove the released oligos, beads were washed with 70% acetonitrile/ water and resuspended in storage buffer.

Synthesised sequences for the Slide-tags experiments (PC in the sequences denote photocleavable linker):

1) Incorporation of capture sequence by ligation: blue colour letters denote the region that is complementary to the sequence of the 10x Gel beads (SLAC beads).

5'-TTT_PC_GCCGGTAATACGACTCACTATAGGGCTACACGACGCTCTTCCGATCTJJJJJJJJTCTTCAGCGTTCCCGAGAJJJJJJJNNNNNNNVVGCTCGGACACATGGGCG-3'

10X FB1 extension:  5’-GAGCTTTGCTAACGGTCGAGGCTTTAAGGCCGGTCCTAGCAA-3’

Splint: 3’-CTGTGTACCCGCCTCGAAACGATTGC-5’

2) Direct synthesis of capture sequence on beads (TAGS beads):

5’-TTT-PC-GTGACTGGAGTTCAGACGTGTGCTCTTCCGATCTJJJJJJJJTCTTCAGCGTTCCCGAGAJJJJJJJNNNNNNNVVGCTTTAAGGCCGGTCCTAGCAA-3’

3) Poly A beads:

5’-TTT-PC-GTGACTGGAGTTCAGACGTGTGCTCTTCCGATCTJJJJJJJJTCTTCAGCGTTCCCGAGAJJJJJJJNNNNNNNVVA30

Array preparation and sequencing were performed as described previously[^20^](https://paperpile.com/c/64twp1/rJlT).

**1.4 Slide-tags procedure**

Fresh frozen tissues were cryo-sectioned to 20 μm on a cryostat (CM1950, Leica) at -16 °C. Pre-cooled 2 mm circular (3331P/25, Integra), 3 mm circular (3332P/25, Integra), or 5.5 mm square custom-made biopsy punches were used to isolate regions of interest from tissue sections. The punched tissue regions were then placed on the puck, ensuring there were no folds. A finger was placed on the bottom of the puck to melt the tissue whilst trying to prevent rolling. Immediately this puck was placed on the glass slide and placed on ice, and 6-10 µL of dissociation buffer (82 mM Na_2_SO_4_, 30 mM K_2_SO_4_, 10 mM Glucose, 10mM HEPES, 5 mM MgCl_2_) was placed on top of the puck so that the buffer covered the whole puck. The puck was then placed under a UV (365 nm) light source (0.42 mW/mm^2^, Thorlabs, M365LP1-C5, Thorlabs, LEDD1B) for 30 s (TAGS beads), or 3 mins (SLAC beads), in order to cleave the same amount of spatial barcode oligonucleotides between bead designs (Extended Data Fig. 2). After photo-cleavage, the puck was incubated for 7.5 mins (TAGS beads) or 5 mins (SLAC beads) and then placed into a 12-well plate (Corning, 3512). Using a 200 µL pipette, 10 x 200 µL aliquots of extraction buffer (Dissociation Buffer, ​1%​ Kollidon VA64, 1% Triton X100, 0.01% BSA, 666 units/mL RNase-inhibitor (Biosearch technologies, 30281-1)) were dispensed onto the puck for a total volume of 2 mL. Dispensed extraction buffer was triturated up and down on the puck for 10-15 times to release the tissue. This step was repeated until the tissue was completely removed from the puck. The puck was removed, and mechanical dissociation of the supernatant was performed using 1 mL pipette 20-25 times trituration to fully dissociate the tissue. Dissociated nuclei were removed from the well and the well was rinsed twice with 1 mL of wash buffer (82 mM Na_2_SO_4_, 30 mM K_2_SO_4_, 10 mM Glucose, 10mM HEPES, 5 mM MgCl_2_, 50 µl of RNase-inhibitor (Biosearch technologies, 30281-1)) which was added to nuclei suspension. Wash buffer was added to the tube to a final volume of 20 mL. This 20 mL was mixed and divided equally into another 50 mL falcon tube. Nuclei were spun in a pre-cooled swinging bucket centrifuge at 600 g for 10 min at 4 °C. After centrifugation, 19.5 mL of supernatant was removed, leaving 500 µL in each tube. The pellet was resuspended and pooled. This pooled suspension was then filtered using a pre-cooled 40 µm cell strainer (Corning, 431750). DAPI (Thermo Fisher Scientific, 62248) was added to the filtered solution at a 1:1000 dilution and incubated for 5-7 mins at 4 °C. This was then centrifuged at 200 g for 10 mins at 4 °C. The supernatant was removed, leaving 50 µL of pellet. The pellet was resuspended and nuclei were counted manually using a C-Chip Fuchs-Rosenthal disposable hemocytometer (INCYTO, DHC-F01-5).

**1.5 Sequencing library preparation**

*snRNA-seq library preparation*

For Slide-tags snRNA-seq experiments, 43.3 µL of counted nuclei were loaded into the 10x Genomics Chromium controller using the Chromium Next GEM Single Cell 3’ Kit v3.1 (10x Genomics, PN-1000268). The Chromium Next GEM Single Cell 3’ Reagent Kits v3.1 (Dual Index) with Feature Barcode technology for Cell Surface Protein CG000317 was used according to the manufacturer’s recommendations with slight modifications. Spatial barcode libraries were prepared as Cell Surface Protein Library preparations. The number of PCR cycles used for the index PCR step in the Cell Surface Protein Library preparation (step  4.1f) for 5.5x5.5 mm TAGS arrays was 7; for 3 mm diameter TAGS arrays the number of cycles was 9.

For the mouse brain sample, ligated pucks (see sequence in section 1.3)  were used for spatial barcoding. For this sample, a custom PCR protocol was used instead of step 4.1: 10 uL of cleaned supernatant from step 2.3, 50 µL NEBNext High-Fidelity 2X PCR Master Mix (NEB, M0541S), 2.5 µL STAG_P701_NEX (10 uM), 2.5 µL 10 μM P5-Truseq Hybrid oligo, 35 µL UltraPure DNase/RNase-Free Distilled Water (Invitrogen, 10977015). In this sample, 10 PCR cycles were performed according to the manufacturer’s recommendations.

*snATAC-seq and snRNA-seq library preparation*

For Slide-tags multiomic snATAC-seq and snRNA-seq experiments, 43.3 µL of counted nuclei were loaded into the 10x Genomics Chromium controller using the Chromium Next GEM Single Cell Multiome ATAC + Gene Expression Reagent Bundle (10x Genomics, PN-1000283). The Chromium Next GEM Single Cell Multiome ATAC + Gene Expression CG000338 Rev F user guide was used according to the manufacturer’s recommendations with slight modifications. During step 4.1, 1 uL of 0.329 uM spike-in primer (5’-GTGACTGGAGTTCAGACGT-3’) was added. For spatial barcode libraries, a custom PCR protocol was used: 5 uL of cleaned supernatant from step 4.3, 50 µL NEBNext High-Fidelity 2X PCR Master Mix (NEB, M0541S), 2.5 µL 10 μM STAG_iP7_a1 oligo (5’-CAAGCAGAAGACGGCATACGAGATATTTACCGCAGTGACTGGAGTTCAGACGT*G*T-3’), 2.5 µL 10 μM P5-STAG_ip5_a1 oligo (5’-AATGATACGGCGACCACCGAGATCTACACGACAATAAAGACACTCTTTCCCTACACGACGC*T*C-3’), 40 µL UltraPure DNase/RNase-Free Distilled Water (Invitrogen, 10977015). In this sample, 15 PCR cycles were performed according to the protocol used in The Chromium Next GEM Single Cell 3’ Reagent Kits v3.1 (Dual Index) with Feature Barcode technology for Cell Surface Protein CG000317 Rev C user guide step 4.1.

*T cell receptor enrichment and library preparation*

We enriched TCRs from Slide-tags multiome cDNA as previously described[^44^](https://paperpile.com/c/64twp1/qrzGP) with the following modifications (<https://www.protocols.io/view/slide-tcr-seq-v3-ivt-n92ldp6w8l5b/v2>).

**1.6 Sequencing**

We sequenced scRNA-seq and spatial barcode libraries on an Illumina Nextseq 1000 instrument using a p2 100 cycle kit (Illumina, 20046811). For some libraries, resequencing was performed to improve sequencing depth, on an Illumina Novaseq instrument using the S Prime platform.

**2 - Slide-tags data preprocessing**

**2.1 snRNA-seq data**

We used Cell Ranger v6.1.2[^1^](https://paperpile.com/c/64twp1/6wOiw) mkfastq (10x Genomics) to generate demultiplexed FASTQ files from the raw sequencing reads. We aligned these reads to either the human GRCh38 or mouse mm10 genome whilst including intronic reads with --include-introns, and quantified gene counts as UMIs using Cell Ranger count (10x Genomics). For mouse embryo, human brain, tonsil, and melanoma, we used CellBender v0.2.0 for background noise correction and cell calling[^61^](https://paperpile.com/c/64twp1/ignMc), setting --expected-cells to the number of Cell Ranger cell calls, --total-droplets-included to 40,000, and --learning-rate to 0.00005 (only when default parameters were insufficient to produce cell probabilities calls of majority zero and one).

**2.2 Multiomic snATAC-seq and snRNA-seq data**

We used Cell Ranger-arc v2.0.2 mkfastq (10x Genomics) to generate demultiplexed FASTQ files from the raw sequencing reads. We aligned these reads to the human GRCh38 genome , and quantified gene counts as UMIs using Cell Ranger-arc count (10x Genomics). For the gene expression data, we then used CellBender for background noise correction and cell calling as described above.

**2.3 Spatial barcode data**

After creating demultiplexed FASTQ files, we grepped for reads containing the spatial barcode universal primer constant sequence. We then downsampled the spatial barcode-containing FASTQ file to 25 million reads using seqtk v1.3-r106 for computational efficiency and consistency across runs. We then matched candidate cell barcodes in the spatial barcode FASTQ file with true cell barcodes outputted from either Cell Ranger v6.1.2 or CellBender[^61^](https://paperpile.com/c/64twp1/ignMc) (Table S14), generating a data frame of candidate spatial barcode sequences per true cell barcode. From this data frame, we matched candidate spatial barcode sequences with a whitelist of *in situ* sequenced spatial barcodes, assigning each true spatial barcode a spatial coordinate.

**2.4 Assignment of spatial locations to nuclei**

Slide-tags nuclei are assigned x, y coordinates corresponding to the distribution of spatial barcodes per nucleus (Supplementary Fig. 1). First, snRNA-seq or multiome data is preprocessed as described above to generate a gene by cell barcode count matrix. The whitelist of cell barcodes from *cellranger* and spatial barcodes from *in situ* bead array sequencing are matched in the spatial barcode FASTQ to generate a spatial barcode by cell barcode matrix. Spatial barcodes with outlier UMI counts (i.e., UMI > 256) are removed as these likely represent beads dislodged from the glass slide during nuclei isolation and encapsulated in droplets with nuclei (data not shown). Then, taking the set of spatial barcodes and their x, y coordinates for each cell barcode, density-based spatial clustering of applications with noise (DBSCAN)[^62,63^](https://paperpile.com/c/64twp1/Me0Mn+ucdjL) v1.1-11 is used to filter out noise spatial barcodes prior to spatial positioning of nuclei (Supplementary Fig. 1c). DBSCAN outputs a cluster assignment for each spatial barcode. Cluster = 0 corresponds to “noise” spatial barcodes without a clear spatial distribution, and cluster numbers greater than zero correspond to “signal” spatial barcodes with discrete spatial clustering. We did not assign spatial positions to nuclei with all spatial barcodes denoted noise, or to nuclei with multiple signal clusters. From the remaining nuclei with one distinct spatial barcode signal cluster, we filtered out noise spatial barcodes and computed a UMI-weighted centroid of spatial barcode coordinates in the signal cluster. DBSCAN required two parameters as input: *minPts* and *eps*. To determine the optimal parameter set for each Slide-tags run, we iterated through *minPts* parameters from *minPts* = 3 to *minPts* = 15 under a constant *eps* = 50 and chose the parameter set with the highest proportion of nuclei that are assigned a spatial position (a single DBSCAN signal cluster). Sankey plot was generated using <https://sankeymatic.com/>.

**2.5 T cell receptor sequences**

TCR sequences were identified using MiXCR v4.1.0[^64,65^](https://paperpile.com/c/64twp1/p88p5+Y4u1A) and assigned to cell barcodes using a hamming distance 1 collapse.

**3 - Mouse brain analysis**

**3.1 - Quality control and cell type assignment**

The output generated by Cell Ranger was read into R (4.1.1) using Seurat (4.3.0)[^21^](https://paperpile.com/c/64twp1/CnKl1). Filtering steps are quantified in Supplementary Figure 1b. We normalised the total UMIs per nucleus to 10,000 (CP10K) and log-transformed these values to report gene expression as E = log(CP10K + 1). We identified the top 2000 highly variable genes after using variance-stabilizing transformation correction[^66^](https://paperpile.com/c/64twp1/pzq2o). All gene expression values were scaled and centred. For visualisation in two dimensions, we embedded nuclei in a Uniform Manifold Approximation and Projection (UMAP)[^67^](https://paperpile.com/c/64twp1/xHAtd) using the top 30 PCs, with: number of neighbours = 40, min_dist = 0.3, spread = 15, local connectivity = 12, and the cosine distance metric. We identified shared nearest neighbours using the top 30 principal components. Clusters of similar cells were detected using the Louvain method for community detection, implemented using *FindClusters*, with a  resolution = 0.8. Each cell was then assigned a predicted identity based on mapping to a mouse adult brain reference dataset[^16^](https://paperpile.com/c/64twp1/O6UAT), using FindTransferAnchors and then TransferData, with the first 25 PCs in both cases. For each computed cell cluster, an identity was assigned using the highest proportion of transferred labels, and confirmed using known markers genes

**3.2 - Assessment of spatial positioning accuracy**

*Spatial barcode metrics calculations*

We measured the accuracy of spatial positioning for the 839 cell barcodes corresponding to high-quality mapped cells in our mouse hippocampus dataset (Fig. 1). For each of these cells, we used the spatial barcodes belonging to the DBSCAN singlet cluster and calculated the standard error for both x and y coordinates using:

$$SE = \frac{\sigma}{\sqrt{N}}$$

Where: N is the number of spatial barcode UMIs in the cluster, and $\sigma$ is the standard deviation of each of the spatial barcode UMIs from the centroid of the cluster.

In addition to the SE, other metrics were calculated for each DBSCAN singlet cluster. Namely, the geometric mean distance of spatial barcodes from the centroid:

$$\overline{x}_{geom}=\left( \prod_{i=1}^{n} \left| \mathcal{x}_{i}-C \right| \right)^{\frac{1}{n}}$$

Where: n is the number of spatial barcode UMIs in the cluster, and xi-C is the absolute distance between each spatial barcode UMI and the cluster centroid.

For each cell that had only a single DBSCAN cluster, additional metrics were calculated (Extended Data Fig. 2d-g). The total number of unique spatial barcode sequences, and spatial barcode UMIs associated with each cell was calculated, regardless of whether it was in the singlet DBSCAN cluster or not. The ratio of spatial barcode UMIs within and outside the DBSCAN singlet cluster was then calculated as the proportion of signal spatial barcodes per cell.

*CA1 width analysis*

A serial section of the profiled region was stained using Nissl and imaged. Cells were segmented from this image via watershed segmentation in Matlab (Release 2021b) and the centroid of each segment was calculated. Next, these coordinates were read into R and DBSCAN was used to isolate cells belonging to the CA1 region, with the following parameters: eps = 35, minPts = 20. The image region was cropped to match that of the profiled Slide-tags region. For both datasets, a 10th (Nissl) or 9th (Slide-tags) order linear model was fitted through these points, generating a central curve. For each spatial barcode UMI, the nearest neighbour on this curve in euclidean space was determined and the distance from these two points was recorded as the distance from the fitted line.

*CA1 sublayer analysis*

Nuclei that belonged to the CA1 cluster were subsetted, and the top 1000 highly variable genes in this subset of nuclei was identified after using variance-stabilizing transformation correction[^66^](https://paperpile.com/c/64twp1/pzq2o). PCA was performed using these variable genes. We identified shared nearest neighbours using the top 25 principal components. Clusters of similar cells were detected using the Louvain method for community detection, implemented using *FindClusters*, with a  resolution = 0.5. Differentially expressed genes between the two clusters were identified using *FindMarkers* with default parameters. Sublayer labels were assigned to each cluster using previously identified gene expression markers[^68,69^](https://paperpile.com/c/64twp1/SLg9i+DJpr3). In situ hybridization data for comparative plots was obtained from the Allen Mouse Brain Atlas[^23^](https://paperpile.com/c/64twp1/OmASk).

**3.3 - Comparison of Slide-tags snRNA-seq vs. snRNA-seq data**

For each sample, cellranger was run as above, and the outputs were run through cellranger aggr (v6.1.2), in order to account for differences in sequencing depth per cell. The result was a combined matrix of 25,158 nuclei, with 25,107 mean reads per cell, 2,309 median UMIs per cell, and 1,438 median genes per cell. The filtered feature-barcode matrix generated by Cell Ranger was then read into R (4.1.1) using Seurat (4.3.0)[^21^](https://paperpile.com/c/64twp1/CnKl1). We normalised the total UMIs per nucleus to 10,000 (CP10K) and log-transformed these values to report gene expression as E = log(CP10K + 1). We identified the top 2000 highly variable genes after using variance-stabilizing transformation correction[^66^](https://paperpile.com/c/64twp1/pzq2o). All gene expression values were scaled and centred. For visualisation in two dimensions, we embedded nuclei in a Uniform Manifold Approximation and Projection (UMAP)[^67^](https://paperpile.com/c/64twp1/xHAtd) using the top 40 PCs, with: number of neighbours = 40, min_dist = 0.3, spread = 15, local connectivity = 12, and the cosine distance metric. We identified shared nearest neighbours using the top 40 principal components. Clusters of similar cells were detected using the Louvain method for community detection, implemented using *FindClusters*, with a  resolution = 1. Each cell was then assigned a predicted identity based on mapping to a mouse adult brain reference dataset[^16^](https://paperpile.com/c/64twp1/O6UAT), using FindTransferAnchors and then TransferData, with the first 25 PCs in both cases. These cell type designations were then used for comparative analysis going forward. Cells designated "Unk_1" or "Unk_2" were removed from the analysis as these cells showed low quality metrics and were not interpretable labels.

**3.4 - Comparison of Slide-tags snRNA-seq vs.** **bulk RNA-seq**

To compare the capture of both Slide-tags snRNA-seq and Slide-seq to bulk RNA-seq data, we used a bulk RNA-seq dataset from the mouse brain that we have previously published[^9^](https://paperpile.com/c/64twp1/Alrq).  To generate this dataset, a stranded mRNA Truseq kit (Illumina #20020594) was used to prepare stranded PolyA selection libraries from a dissected sagittal mouse hippocampus. The libraries were sequenced and transcripts per million (TPM) for each gene were generated using RSEM[^70^](https://paperpile.com/c/64twp1/kWc9H) post alignment with STAR[^71^](https://paperpile.com/c/64twp1/riBtS). For Slide-seq data, we used two previously published datasets: Slide-seqV1[^9^](https://paperpile.com/c/64twp1/Alrq) Puck_180819_6, Slide-seqV2[^20^](https://paperpile.com/c/64twp1/rJlT) Puck_200115_08. Average transcripts per million (APTM) was computed by summing counts for each gene, across all beads on a puck, and dividing by the sum of all UMIs on the puck, and dividing by 1 million (total UMI count/1million). For Slide-tags snRNA-seq data, in order to make an appropriate comparison, data was quantified to exclude intronic reads. Average transcripts per million (APTM) was then computed by summing counts for each gene, across all nuclei on the puck used in Figure 1g-i, and dividing by the sum of all UMIs across all nuclei, and dividing by 1 million (total UMI count/1million). The per-gene distribution for each of these values (bulk TPM and Slide-seq ATPM) was plotted and linear regression was performed to calculate pearson’s correlation coefficient.

**3.5 - Comparison of Slide-tags snRNA-seq with Slide-seqV2 and DBiT-seq**

Slide-tags snRNA-seq mouse hippocampus data was compared with Slide-seqV2[^20^](https://paperpile.com/c/64twp1/rJlT) mouse brain data and DBiT-seq mouse brain data (Spatial-ATAC-RNA-seq[^13^](https://paperpile.com/c/64twp1/fD3LN)). For gene and UMI count comparisons, Slide-seq data was spatially binned to 20 μm spatial square pixels. Slide-tags snRNA-seq data was processed and nuclei were embedded in UMAP space as described above. Slide-seqV2 and DBiT-seq total UMIs per spatial spot (10 μm beads in Slide-seqV2) were normalised to 10,000 (CP10K) and log-transformed to report gene expression as E = log(CP10K + 1). The top 2000 highly variable genes were identified after using variance-stabilizing transformation correction[^66^](https://paperpile.com/c/64twp1/pzq2o). Gene expression values were scaled and centred. For visualisation in two dimensions, we embedded spatial spots in a UMAP space using the top 30 PCs, with: number of neighbours =30, min_dist = 0.3, spread =1, local connectivity = 1, and the cosine distance metric. We identified shared nearest neighbours using the top 30 principal components. For Slide-seqV2 clusters of similar cells were detected using the Louvain method for community detection, implemented using *FindClusters*, with a  resolution = 1. RNA clusters from the Spatial-ATAC-RNA-seq publication[^13^](https://paperpile.com/c/64twp1/fD3LN) were used for DBiT-seq data. Standard deviations for the top 30 principal components were plotted using *ElbowPlot* in Seurat. Dotplots display SCTransformed expression values for DBiT-seq from the Spatial-ATAC-RNA-seq publication.

**4 - Mouse embryonic brain at E14 analysis**

The output generated by Cell Ranger was read into R (4.1.1) using Seurat (4.3.0)[^21^](https://paperpile.com/c/64twp1/CnKl1). We normalised the total UMIs per nucleus to 10,000 (CP10K) and log-transformed these values to report gene expression as E = log(CP10K + 1). We identified the top 2000 highly variable genes after using variance-stabilizing transformation correction[^66^](https://paperpile.com/c/64twp1/pzq2o). All gene expression values were scaled and centred. For visualisation in two dimensions, we embedded nuclei in a Uniform Manifold Approximation and Projection (UMAP)[^67^](https://paperpile.com/c/64twp1/xHAtd) using the top 30 PCs, with: number of neighbours = 40, min_dist = 0.3, spread = 15, local connectivity = 12, and the cosine distance metric. We identified shared nearest neighbours using the top 30 principal components. Clusters of similar cells were detected using the Louvain method for community detection, implemented using *FindClusters*, with a  resolution = 0.8. Each cell was then assigned a predicted identity based on mapping to a mouse embryo at E14 reference dataset[^18^](https://paperpile.com/c/64twp1/h4CNm), using FindTransferAnchors and then TransferData, with the first 25 PCs in both cases. For each computed cell cluster, an identity was assigned using the highest proportion of transferred labels, and confirmed using known marker genes.

**5 - Human brain analysis**

**5.1 - Quality control and cell type assignment**

The output generated by Cell Ranger was filtered by CellBender and read into R (4.2.2). The matrix was subsetted down to cells that had exactly one DBSCAN location and fewer than 5% mitochondrial UMIs, which were then loaded into Seurat (4.3.0)[^21^](https://paperpile.com/c/64twp1/CnKl1) to perform normalisation, finding variable features, scaling, PCA, finding neighbours (dims=30), finding clusters, and creating a UMAP, all with default parameters (unless specified otherwise). Each cluster was assigned a cell class (Excitatory neuron, Inhibitory neuron, Oligodendrocyte (Oligo), Oligo precursor cell (OPC), Astrocyte (AS), Endothelial cell, Microglia) by plotting canonical cell type marker genes on the UMAP and manually assigning each cluster a cell type. Subsequently, excitatory and inhibitory neuron subtypes were mapped from a published human cortex dataset[^25^](https://paperpile.com/c/64twp1/GgYS6) by label transfer using Harmony v0.1.1 and spatially plotted in Supplementary Fig. 2b and 3b.

**5.2 - Identification of layers and layer-dependent gene expression**

The layer assignment of each cell (L1-2, L3-5, L6, WM) was calculated by manually drawing boundaries between the layer-specific mapped neuron subtypes and assigning each cell a label depending on which two boundaries it was between. The numerical laminar coordinate was then calculated by taking the Euclidean distance of each cell to the nearest boundary and dividing it by the sum of the distances to the two neighbouring boundaries, adding a constant factor depending on the layer assignment.

Before computing the spatial variation score for each gene, nuclei were removed if they contained expression above a Z-score of 2 for a marker gene of a different cell type. Subsequently, each gene was assigned a spatial variation score by computing the kernelized density of the gene expression along the laminar coordinate of filtered cells using a uniform kernel and taking the difference between the highest and lowest expression density values (Table S2). Complex gradients were found by taking the intersection of each cell type’s spatially variable gene list, and a visually-selected interesting subset is shown in Fig. 2l.

Gene ontology analysis was performed on all genes with a spatial variation Z-score above 7.0 using EnrichGO from clusterProfiler 4.6.0 (default parameters) and using annotations from org.Hs.eg.db v3.16.0 (Table S3) under the Biological Process (BP) ontology. For display in Fig. 2k, the terms were further subsetted to only include terms with an adjusted p-value below 1e-8 in at least one cell type.

Genes with a spatial variation Z-score above 10 in excitatory/inhibitory neurons and above 8 in astrocytes/OPCs are shown in the heatmaps in Fig. 2i-j, Extended Data Fig. 6d-e. Genes that additionally had a minimum expression below 0.8 were spatially plotted in Supplementary Fig. 4-6.

**5.3 - Reproducibility Analysis**

The percentage of high-quality nuclei that were spatially positioned and the density of mapped nuclei were compared across four human cortex slide-tags runs for display in Supplementary Table 4. For each run, the cell calls generated as output by Cell Ranger were used and low-quality cells were removed if they belonged to a cluster with an average mitochondrial nUMIs % greater than 5%. Then, the percentage of mapped nuclei was computed by dividing the number of nuclei with exactly one DBSCAN location by the total number of nuclei. The nuclei density was calculated by selecting a window of tissue with equal white and grey matter area and dividing the number of spatially positioned nuclei in the window by the window area.

**6 - Tonsil analysis**

**6.1 - Quality control and cell type assignment**

The output generated by Cell Ranger and filtered by CellBender was read into R (4.1.1) using Seurat (4.3.0)[^21^](https://paperpile.com/c/64twp1/CnKl1). We normalised the total UMIs per nucleus to 10,000 (CP10K) and log-transformed these values to report gene expression as E = log(CP10K + 1). We identified the top 2000 highly variable genes after using variance-stabilising transformation correction[^66^](https://paperpile.com/c/64twp1/pzq2o). All gene expression values were scaled and centred. For visualisation in two dimensions, we embedded nuclei in a Uniform Manifold Approximation and Projection (UMAP)[^67^](https://paperpile.com/c/64twp1/xHAtd) using the top 30 PCs, with: number of neighbours =30, min_dist = 0.3, spread =1, local connectivity = 1, and the cosine distance metric. We identified shared nearest neighbours using the top 30 principal components. Clusters of similar cells were detected using the Louvain method for community detection, implemented using *FindClusters*, with a  resolution = 1. Annotation of *de novo* clusters was aided by marker genes and Azumith[^21^](https://paperpile.com/c/64twp1/CnKl1) reference-based mapping from the human tonsil atlas[^72^](https://paperpile.com/c/64twp1/bAS6J).

**6.2 - Spatially varying gene expression**

Significantly nonrandom genes were discovered in germinal centre B cells as described previously[^9^](https://paperpile.com/c/64twp1/Alrq). Briefly, for each single-nucleus assigned as a germinal centre B cell that was positioned in one of the four largest germinal centres we profiled,  we first calculated the matrix of pairwise Euclidean distances between cells for each germinal centre individually. We then compared the distribution of pairwise distances between the cells expressing at least one count of that transcript to the distribution of pairwise distances between an identical number of cells, sampled randomly from all mapped beads within the set with probability proportional to the total number of UMIs per cell. Specifically, we generated 1000 such random samples, and for each sample calculated the distribution of pairwise distances. We then calculated the average distribution of pairwise distances, averaged across all 1000 samples. Finally, we calculated the L1 norm between the distribution of pairwise distances for the true sample of cells and the average distribution. We defined p to be the fraction of random samples having distributions closer to the average distribution (under the L1 norm) than the true sample. We calculated an Z-score for the true sample given the distribution distances from the average distribution of random samples. Finally, we aggregated *p* values for spatial variation from each of the four tested germinal centres using Fisher’s method.

We intersected our computed spatially varying genes with genes previously implicated in germinal centre zone distinction[^73^](https://paperpile.com/c/64twp1/T9yF1). We calculated percent variance in gene expression space and plotted against spatial effect size from our spatial permutation test to identify genes with relatively low gene expression variance but high spatial variance.

**6.3 - Germinal centre zonation**

We used spatially varying genes (*p* value < 0.05) identified as described above to classify germinal centre B cells into light zone, dark zone, and transitional states. Specifically, we subsetted our data to germinal centre B cells, re-scaled and re-centred values, and ran PCA on the 1068 significant spatially varying genes. We then identified shared nearest neighbours using the top 15 principal components. Clusters of similar cells were detected using the Louvain method for community detection, implemented using *FindClusters*, with resolution = 0.4. We annotated clusters as light zone, dark zone, and transitional states using marker genes and Azumith[^21^](https://paperpile.com/c/64twp1/CnKl1) reference-based mapping from the human tonsil atlas[^72^](https://paperpile.com/c/64twp1/bAS6J).

After classifying germinal centre B cells into states, we spatially segmented germinal centres into light zones and dark zones using dark zone B cell spatial density. We ran DBSCAN[^62^](https://paperpile.com/c/64twp1/Me0Mn) on dark zone B cells of the two largest germinal centres, using *eps* = 60 and *minPts* = 6 for the largest germinal centre, and *eps* = 60 and *minPts* = 10 for the second largest germinal centre. We considered cells within the top DBSCAN cluster to constitute the dark zone and segmented around the outer cells. The remaining cells in both germinal centres were considered to be in the light zone and segmentation borders were drawn accordingly. We tested for zone bias of T follicular helper cells and follicular dendritic cells using *chisq.test* from the stats package in R (4.2.2).

**6.4 - Spatial receptor-ligand prediction**

To detect receptor-ligand interactions between cell-type pairs, we computed a receptor-ligand score based on a spatial correlation index[^74^](https://paperpile.com/c/64twp1/p9CKs), SCI, which we defined as:

$$SCI = \frac{\sum_{i}^{N} \sum_{j}^{M} w_{ij}r_{i}l_{j}}{\sum_{i}^{N} \sum_{j}^{M} w_{ij}}$$

between N cells of “sender cell type” expressing receptor r and M cells of “receiver cell type” expressing ligand l, where expression is sctransform counts[^75^](https://paperpile.com/c/64twp1/MQ8pZ). We defined the spatial weights matrix of dimensionality NxM as an adjacency matrix, denoting 1 for when sender cell i is within 100 um of receiver cell j and 0 otherwise. We first ran LIANA[^33^](https://paperpile.com/c/64twp1/ZloZH) (0.1.12) to generate a putative list of receptor-ligand interactions between cell-type pairs in a spatial agnostic way, filtering to receptor-ligand interactions that are expressed in at least 50 cells of sender and receiver cell types (log CPM > 0), or in 30% of sender and receiver cells. We then computed a spatial correlation index for each receptor-ligand interaction to determine if the receptor and ligand are spatially co-expressed in a given cell-type pair.

To determine the spatial significance of a receptor-ligand score, we employed an adaptive spatial permutation test, running 1000 permutations for each receptor-ligand interaction. In each permutation, we randomly permuted the spatial locations of cells within a given cell-type. For interactions that have a nominal *p* value less than or equal to 0.005, we ran an additional 9000 permutations. We corrected for multiple hypothesis testing using the Benjamini-Hochberg procedure. We also computed the log-fold change between the observed SCI statistic and the median SCI statistic of the empirical null distribution. This allowed us to compare SCI log-fold change values between receptor-ligand interactions for different cell types without explicitly correcting for the number of cells of each cell type.

**6.5 - Spatial contextualization of receptor-ligand interactions**

To spatially contextualise receptor-ligand interactions, we decomposed spatial correlation indices for each significant interaction between germinal centre B cells, T follicular helper cells, and follicular dendritic cells (adj. *p* value < 0.05) into interaction intensity scores for individual cells[^76^](https://paperpile.com/c/64twp1/DhMmO). These decomposed scores reflect each individual cell’s contribution to the total spatial correlation index, defined as follows for receiving cell i and vice-vera's for sender cell j:

$$LISA = \frac{r_{i}\sum_{j}^{M} w_{ij}l_{j}}{\sum_{j}^{M} w_{ij}}$$

We tested germinal centre zone specificity via *wilcox.test* in R comparing interaction intensity scores of the receptor of each cell between dark zones and light zones. We corrected *p* values using the Benjamini-Hochberg method. Zone-specific receptor expression was tested using SCTransformed expression values compared between dark zones and light zones also using *wilcox.test* in R.

**7 - Melanoma analysis**

**7.1 - Quality control and cell type assignment**

*snRNA-seq data*

The Cell Ranger output was filtered by CellBender and read into R (4.1.1) using Seurat (4.3.0)[^21^](https://paperpile.com/c/64twp1/CnKl1). We normalised the total UMIs per nucleus to 10,000 (CP10K) and log-transformed these values to report gene expression as E = log(CP10K + 1). We identified the top 2000 highly variable genes after using variance-stabilising transformation correction[^66^](https://paperpile.com/c/64twp1/pzq2o). All gene expression values were scaled and centred. For visualisation in two dimensions, we embedded nuclei in a Uniform Manifold Approximation and Projection (UMAP)[^67^](https://paperpile.com/c/64twp1/xHAtd) using the top 30 PCs, with: number of neighbours =30, min_dist = 0.3, spread =1, local connectivity = 1, and the cosine distance metric. We identified shared nearest neighbours using the top 30 principal components. Clusters of similar cells were detected using the Louvain method for community detection, implemented using *FindClusters*, with a  resolution = 1. Annotation of *de novo* clusters was aided by marker genes.

*Multiome ATAC & snRNA-seq data*

The RNA expression matrix generated by Cell Ranger was read into R (4.1.1) using Seurat[^21^](https://paperpile.com/c/64twp1/CnKl1). The ATAC filtered feature-barcode matrix generated by Cell Ranger was read into R (4.1.1) using Signac (1.9.0)[^77^](https://paperpile.com/c/64twp1/bWr4w), and added as its own assay slot in the Seurat object containing RNA expression counts. Peaks were recalled using the CallPeaks function, which uses MACS2 (2.2.7.1)[^78^](https://paperpile.com/c/64twp1/OgZrr), across all cells. Fragments were mapped to the MACS2-called peaks and assigned to nuclei using the FeatureMatrix function in Signac. Peaks in non-standard chromosomes were removed using keepStandardChromosomes from GenomeInfoDb (1.35.15)[^79^](https://paperpile.com/c/64twp1/mZP2j), and problematic regions of the hg38 genome were removed using subsetByOverlaps according to the blacklist available at: https://github.com/Boyle-Lab/Blacklist[^80^](https://paperpile.com/c/64twp1/HbnrU). This final peaks-barcode matrix was then added to the “peaks” assay within the Seurat object.

For cell type annotation, the snRNA-seq data from the multiome experiment was normalised for the total UMIs per nucleus to 10,000 (CP10K) and log-transformed to report gene expression as E = log(CP10K + 1). The top 2000 highly variable genes were identified after using variance-stabilising transformation correction[^66^](https://paperpile.com/c/64twp1/pzq2o). We then integrated the gene expression data from Slide-tags multiome with gene expression data from Slide-tags snRNA-seq using SelectIntegrationFeatures, FindIntegrationAnchors, and IntegrateData across all features with default parameters from Seurat (4.3.0). Integrated gene expression values were scaled and centred. For visualisation in two dimensions, we embedded nuclei in a Uniform Manifold Approximation and Projection (UMAP)[^67^](https://paperpile.com/c/64twp1/xHAtd) using the top 30 PCs, with: number of neighbours =30, min_dist = 0.3, spread =1, local connectivity = 1, and the cosine distance metric. We identified shared nearest neighbours using the top 30 principal components. Clusters of similar cells were detected using the Louvain method for community detection, implemented using *FindClusters*, with a resolution = 1. Cells from Slide-tags multiome were annotated based on marker genes and co-clustering with Slide-tags snRNA-seq cells. Gene expression counts from Slide-tags multiome were re-scaled and re-cluster as described above using the non-integrated object for subsequent analyses.

**7.2 - Inferring copy number variation**

InferCNV (1.3.3) was used to infer large-scale copy number variation from standard snRNA-seq data and from snRNA-seq data from a 10x multiome experiment as previously recommended (inferCNV of the Trinity CTAT Project, <https://github.com/broadinstitute/inferCNV>). CellBender-corrected counts were extracted from annotated Seurat objects, where normal reference cells were specified as all cells not labelled as tumour. InferCNV was run under the following parameters: cutoff = 0.1, cluster_by_groups = T, denoise = T, HMM = T, num_threads = 60.

**7.3 - T cell receptor analysis**

TCR analyses focused on CD8 T cells where we used Fisher’s exact test to test if: (1)  the beta chain sequence CASRASNEQFF was tumour compartment biassed compared against all CD8 T cells with profiled beta chains, where tumour compartment segmentation was performed manually based on tumour subpopulation density; and (2) paired CD8 T cells with TCR alpha chain CAEWYNQGGKLIF and beta chain CASRASNEQFF were tumour compartment biassed.

**7.4 - ATAC analysis**

Latent semantic indexing (LSI) was performed on the peaks assay using Signac, with the RunTFIDF and RunSVD functions.  For visualisation in two dimensions, we embedded nuclei in a Uniform Manifold Approximation and Projection (UMAP)[^67^](https://paperpile.com/c/64twp1/xHAtd) using LSI dimensions 2-30. Nuclei were visualised using the combination of modalities profiled, with weighted-nearest neighbour (WNN) analysis. Multimodal neighbours were identified using Seurat’s FindMultiModalNeighbors function, with the RNA PCA dimensions 1:50, and the ATAC LSI dimensions 2:50. These neighbours were then used as input into RunUMAP for visualisation.

In order to annotate the motifs present in peaks, the Signac function CreateMotifObject was used to create a motif object, with all human motifs from the Jaspar 2020 database. Motif accessibility z-scores were then calculated using Signac’s RunChromVAR function (ChromVAR 1.16.0). Gene activity scores were calculated using the Signac function GeneActivity. We normalised these gene scores by the total gene score per nucleus to the median nUMI for the RNA assay (NGS) and log-transformed these values to report gene expression as E = log(NGS + 1).

**7.5 - Differential gene expression, differential chromatin gene scores, and gene set enrichment analysis**

Differential gene expression analyses were performed using the MAST implemented in FindMarkers from Seurat[^81^](https://paperpile.com/c/64twp1/105uS). Analysis comparing tumour cluster 1 and tumour cluster 2  from Slide-tags snRNA-seq and comparing compartment-specific CD8 T cells from Slide-tags multiome data used min.pct = 0.25 and log2fc.threshold = 0.25. Analysis comparing tumour cluster 1 and tumour cluster 2 from Slide-tags multiome data used min.pct = 0.1 and log2fc.threshold = 0.25. Gene ontology biological process (GO_Biological_Process_2021) gene set enrichment analysis was performed with the Enrichr package (3.1) in R[^82–84^](https://paperpile.com/c/64twp1/mdRLY+xcj6C+Q38lQ) on tumour cluster 2 enriched differentially expressed genes with log2FC < -0.5 and adjust *p* value < 0.05. Differential chromatin gene score analysis was conducted using the Wilcoxon Rank Sum test implemented in FindMarkers from Seurat with min.pct = 0.1 and log2fc.threshold = 0.

**7.6 - Melanocytic-like and mesenchymal-like signatures**

We scored tumour cells on melanocytic-like and mesenchymal-like signatures using AddModuleScore in Seurat with a list of genes adapted from previous work (Table S15)[^46,85^](https://paperpile.com/c/64twp1/ySeoB+ho82z). Correlations of chromVar motif scores with mesenchymal scores were tested using Pearson’s correlation coefficient and *p* values were corrected using the Benjamini-Hochberg procedure. Spatial autocorrelations of chromVar motifs were tested using Moran.I from the ape package (5.6-2) in R[^86^](https://paperpile.com/c/64twp1/WmtFf), where the weights matrix was specified as 1/distance^2^.

Supplementary References

61. Fleming, S. J. et al. Unsupervised removal of systematic background noise from droplet-based single-cell experiments using CellBender. bioRxiv 791699 (2022) doi:10.1101/791699.

62. Ester, M., Kriegel, H.-P., Sander, J. & Xu, X. A density-based algorithm for discovering clusters in large spatial databases with noise. in Proceedings of the Second International Conference on Knowledge Discovery and Data Mining 226–231 (AAAI Press, 1996).

63. Hahsler, M., Piekenbrock, M. & Doran, D. dbscan: Fast Density-Based Clustering with R. J. Stat. Softw. 91, 1–30 (2019).

64. Bolotin, D. A. et al. MiXCR: software for comprehensive adaptive immunity profiling. Nat. Methods 12, 380–381 (2015).

65. Bolotin, D. A. et al. Antigen receptor repertoire profiling from RNA-seq data. Nat. Biotechnol. 35, 908–911 (2017).

66. Stuart, T. et al. Comprehensive Integration of Single-Cell Data. Cell vol. 177 1888–1902.e21 Preprint at https://doi.org/10.1016/j.cell.2019.05.031 (2019).

67. McInnes, L., Healy, J. & Melville, J. UMAP: Uniform Manifold Approximation and Projection for Dimension Reduction. arXiv [stat.ML] (2018).

68. Cid, E. et al. Sublayer- and cell-type-specific neurodegenerative transcriptional trajectories in hippocampal sclerosis. Cell Rep. 35, 109229 (2021).

69. Dong, H.-W., Swanson, L. W., Chen, L., Fanselow, M. S. & Toga, A. W. Genomic-anatomic evidence for distinct functional domains in hippocampal field CA1. Proc. Natl. Acad. Sci. U. S. A. 106, 11794–11799 (2009).

70. Li, B. & Dewey, C. N. RSEM: accurate transcript quantification from RNA-Seq data with or without a reference genome. BMC Bioinformatics 12, 323 (2011).

71. Dobin, A. et al. STAR: ultrafast universal RNA-seq aligner. Bioinformatics 29, 15–21 (2013).

72. Massoni-Badosa, R. et al. An Atlas of Cells in the Human Tonsil. Preprint at https://doi.org/10.1101/2022.06.24.497299.

73. Victora, G. D. et al. Identification of human germinal center light and dark zone cells and their relationship to human B-cell lymphomas. Blood 120, 2240–2248 (2012).

74. Chen, Y. A new methodology of spatial cross-correlation analysis. PLoS One 10, e0126158 (2015).

75. Choudhary, S. & Satija, R. Comparison and evaluation of statistical error models for scRNA-seq. Genome Biol. 23, 27 (2022).

76. Anselin, L. Local indicators of spatial association-LISA. Geogr. Anal. 27, 93–115 (2010).

77. Stuart, T., Srivastava, A., Madad, S., Lareau, C. A. & Satija, R. Single-cell chromatin state analysis with Signac. Nat. Methods 18, 1333–1341 (2021).

78. Zhang, Y. et al. Model-based analysis of ChIP-Seq (MACS). Genome Biol. 9, R137 (2008).

79. Arora, S., Morgan, M., Carlson, M. & Pagès, H. GenomeInfoDb: Utilities for manipulating chromosome names, including modifying them to follow a particular naming style. Preprint at https://bioconductor.org/packages/GenomeInfoDb (2023).

80. Amemiya, H. M., Kundaje, A. & Boyle, A. P. The ENCODE Blacklist: Identification of Problematic Regions of the Genome. Sci. Rep. 9, 9354 (2019).

81. Finak, G. et al. MAST: a flexible statistical framework for assessing transcriptional changes and characterizing heterogeneity in single-cell RNA sequencing data. Genome Biol. 16, 278 (2015).

82. Chen, E. Y. et al. Enrichr: interactive and collaborative HTML5 gene list enrichment analysis tool. BMC Bioinformatics 14, 128 (2013).

83. Kuleshov, M. V. et al. Enrichr: a comprehensive gene set enrichment analysis web server 2016 update. Nucleic Acids Res. 44, W90–7 (2016).

84. Xie, Z. et al. Gene Set Knowledge Discovery with Enrichr. Curr Protoc 1, e90 (2021).

85. Widmer, D. S. et al. Systematic classification of melanoma cells by phenotype-specific gene expression mapping. Pigment Cell Melanoma Res. 25, 343–353 (2012).

86. Paradis, E. & Schliep, K. ape 5.0: an environment for modern phylogenetics and evolutionary analyses in R. Bioinformatics 35, 526–528 (2019).
