# Supplementary material for: Slide-tags enables single-nucleus barcoding for multimodal spatial genomics
Source: Nature. 2023 Dec 13;625(7993):101–9. doi: 10.1038/s41586-023-06837-4 (PMC10764288; doi:10.1038/s41586-023-06837-4)
Supplement: Supplementary file 2 — Reporting Summary [file 41586_2023_6837_MOESM2_ESM.pdf]

nature portfolio

Corresponding author(s): Fei Chen
Evan Macosko

Last updated by author(s): Oct 2, 2023

Reporting Summary

Nature Portfolio wishes to improve the reproducibility of the work that we publish. This form provides structure for consistency and transparency in reporting. For further information on Nature Portfolio policies, see our Editorial Policies and the Editorial Policy Checklist.

Statistics

For all statistical analyses, confirm that the following items are present in the figure legend, table legend, main text, or Methods section.

n/a	Confirmed	
☐	☒	The exact sample size (*n*) for each experimental group/condition, given as a discrete number and unit of measurement
☐	☒	A statement on whether measurements were taken from distinct samples or whether the same sample was measured repeatedly
☐	☒	The statistical test(s) used AND whether they are one- or two-sided<br>*Only common tests should be described solely by name; describe more complex techniques in the Methods section.*
☒	☐	A description of all covariates tested
☐	☒	A description of any assumptions or corrections, such as tests of normality and adjustment for multiple comparisons
☐	☒	A full description of the statistical parameters including central tendency (e.g. means) or other basic estimates (e.g. regression coefficient) AND variation (e.g. standard deviation) or associated estimates of uncertainty (e.g. confidence intervals)
☐	☒	For null hypothesis testing, the test statistic (e.g. *F*, *t*, *r*) with confidence intervals, effect sizes, degrees of freedom and *P* value noted<br>*Give P values as exact values whenever suitable.*
☒	☐	For Bayesian analysis, information on the choice of priors and Markov chain Monte Carlo settings
☒	☐	For hierarchical and complex designs, identification of the appropriate level for tests and full reporting of outcomes
☐	☒	Estimates of effect sizes (e.g. Cohen's *d*, Pearson's *r*), indicating how they were calculated

Our web collection on statistics for biologists contains articles on many of the points above.

Software and code

Policy information about availability of computer code

Data collection	For alignment of sequencing reads, we used: Cell Ranger v6.1.2, CellBender v0.2.0, Cell Ranger-arc v2.0.2, seqtk v1.3-r106.
Data analysis	For processing of aligned data, MiXCR v4.l.0, MACS2 v2.2. 7.1, inferCNV vl.3.3.<br>The following R packages were used within R 4.1.1: DBSCAN vl.1-11, Seurat v4.3.0, Harmony v0.1.1, stats v4.2.2, Signac vl.9.0, GenomelnfoDb vl.35.15, LIANA v0.l.12, Enrichr v3.l, ape v5.6-2. The following R packages have been used in R 4.2.2: Seurat v4.3.0. Dependencies have not been listed for brevity.<br><br>Code for processing spatial sequencing libraries is available on Github: https://github.com/broadchenf/Slide-tags.

For manuscripts utilizing custom algorithms or software that are central to the research but not yet described in published literature, software must be made available to editors and reviewers. We strongly encourage code deposition in a community repository (e.g. GitHub). See the Nature Portfolio guidelines for submitting code & software for further information.

Data

Policy information about availability of data

All manuscripts must include a data availability statement. This statement should provide the following information, where applicable:

- Accession codes, unique identifiers, or web links for publicly available datasets
- A description of any restrictions on data availability
- For clinical datasets or third party data, please ensure that the statement adheres to our policy

Slide-tags datasets have been deposited on the Broad Institute Single Cell Portal, under the following accession numbers: mouse brain (SCP2162), mouse embryonic brain (SCP2170), human brain (SCP2167), human tonsil (SCP2169), human melanoma (SCP2171), human melanoma multiome (SCP2176). Raw and processed mouse data has been deposited in GEO under the accession number: GSE244355.

Human research participants

Policy information about studies involving human research participants and Sex and Gender in Research.

Reporting on sex and gender	Human brain data was from female. No gender available for human tonsil and melanoma specimens due to excess surgical collection from deidentified subjects.
Population characteristics	Human brain data was from 78 year old female. No age infomration available for human tonsil and melanoma specimens due to excess surgical collection from deidentified subjects. Melanoma specimens were acquired from a patient who underwent axillary lymphadenectomy for metastatic BRAF-mutant melanoma prior to starting PD-1 inhibitor.
Recruitment	No recruitment was done. Human tonsil and melanoma specimens collected from excess surgical material from deidentified subjects. Human brain sample was from post-mortem deidentified specimen.
Ethics oversight	This was determined to be non-human subject research by Broad IBC.

Note that full information on the approval of the study protocol must also be provided in the manuscript.

Field-specific reporting

Please select the one below that is the best fit for your research. If you are not sure, read the appropriate sections before making your selection.

☒ Life sciences ☐ Behavioural & social sciences ☐ Ecological, evolutionary & environmental sciences

For a reference copy of the document with all sections, see nature.com/documents/nr-reporting-summary-flat.pdf

Life sciences study design

All studies must disclose on these points even when the disclosure is negative.

Sample size	No sample size calculation was performed. Samples sizes were chosen primarily based on experiment length, sample availablity, and sequencing costs. These sample sizes are sufficient because each sample serves as a proof-of-concept for the new technology.
Data exclusions	No data was excluded.
Replication	All attempts at replication were successful. We performed replication on human brain Slide-tags datasets (2 technical datasets).
Randomization	Randomization was not applicable because the focus of this paper is the development of a new genomic technology and did not involve allocating samples/organisms/participants into experimental groups.
Blinding	Blinding was not applicable because because the focus of this paper is the development of a new genomic technology and did not involve group allocation.

Reporting for specific materials, systems and methods

We require information from authors about some types of materials, experimental systems and methods used in many studies. Here, indicate whether each material, system or method listed is relevant to your study. If you are not sure if a list item applies to your research, read the appropriate section before selecting a response.

Materials & experimental systems

n/a	Involved in the study
☒	☐ Antibodies
☒	☐ Eukaryotic cell lines
☒	☐ Palaeontology and archaeology
☐	☒ Animals and other organisms
☒	☐ Clinical data
☒	☐ Dual use research of concern

Methods

n/a	Involved in the study
☒	☐ ChIP-seq
☒	☐ Flow cytometry
☒	☐ MRI-based neuroimaging

Animals and other research organisms

Policy information about studies involving animals; ARRIVE guidelines recommended for reporting animal research, and Sex and Gender in Research

Laboratory animals	Mus musculus strain C57BL/6J 56 days old, Mus musculus strain C57 E14
Wild animals	This study did not involve wild animals.
Reporting on sex	Sex was not important for this study since the tissues are used to benchmark a new genomics protocol, which we anticipate would provide identical results regardless of sex.
Field-collected samples	No field-collected samples were used.
Ethics oversight	All procedures involving animals at the Broad Institute were conducted in accordance with the US National Institutes of Health Guide for the Care and Use of Laboratory Animals under protocol number 0120-09-16

Note that full information on the approval of the study protocol must also be provided in the manuscript.
