# Supplementary material for: Slide-tags enables single-nucleus barcoding for multimodal spatial genomics
Source: Nature. 2023 Dec 13;625(7993):101–9. doi: 10.1038/s41586-023-06837-4 (PMC10764288; doi:10.1038/s41586-023-06837-4)
Supplement: Supplementary file 3 — Supplementary Figs. 1–8. [file 41586_2023_6837_MOESM3_ESM.pdf]

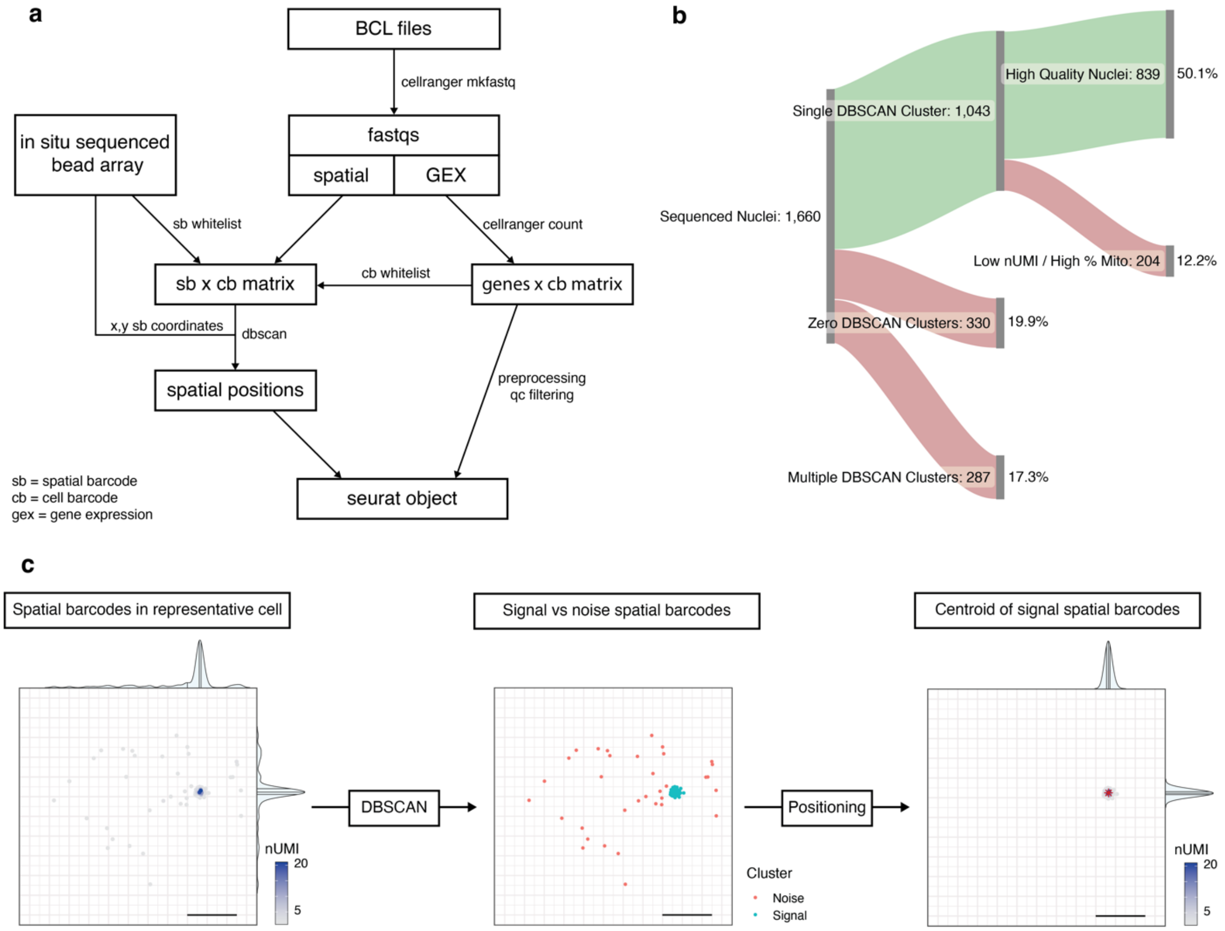

Supplementary Figure. 1. Computational pipeline for spatial positioning of Slide-tags nuclei. a, Summary of computational pipeline for processing Slide-tags data and spatially positioning nuclei. **b,** Nuclei loss steps quantified throughout Slide-tags data processing for the mouse hippocampus Slide-tags snRNA-seq experiment. Single DBSCAN cluster nuclei refer to those that can be confidently assigned a spatial position. Nuclei are not assigned a spatial position if they have zero DBSCAN clusters or multiple DBSCAN clusters. Nuclei with low UMI counts and high percentages of mitochondrial reads were removed. **c,** Example of a representative nucleus processed with DBSCAN. The red star denotes the spatial position of example cell 2, defined as the UMI-weighted centroid of signal spatial barcodes. Scale bars denote 500 μm.

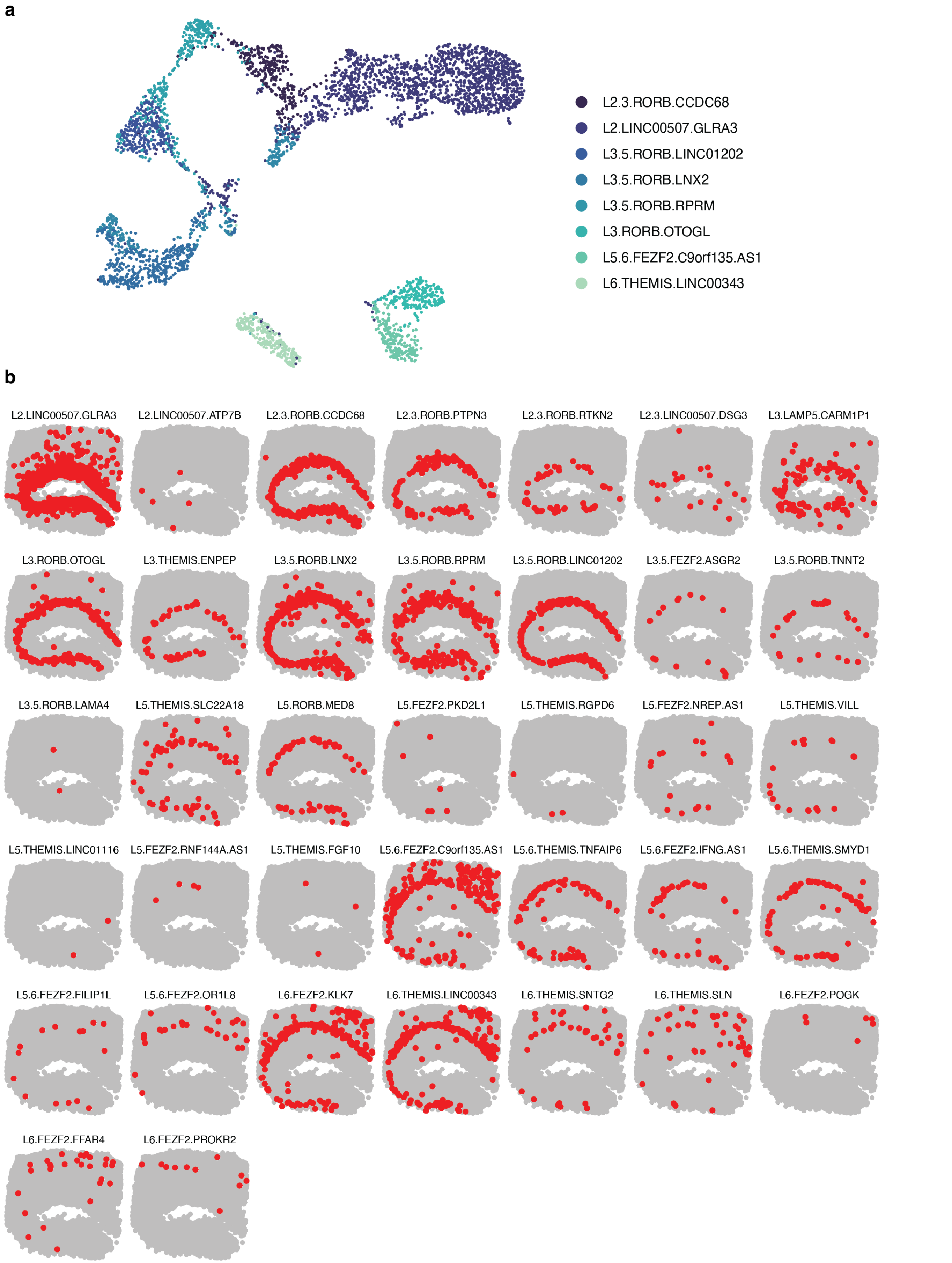

Supplementary Figure. 2. Slide-tags snRNA-seq in the human brain enables mapping of excitatory neuron sub-cell-types. a, UMAP and label transfer of principal excitatory subtypes. **b,** Excitatory neuron subtypes plotted by spatial location. Subtype names from Bakken et al., 2021.

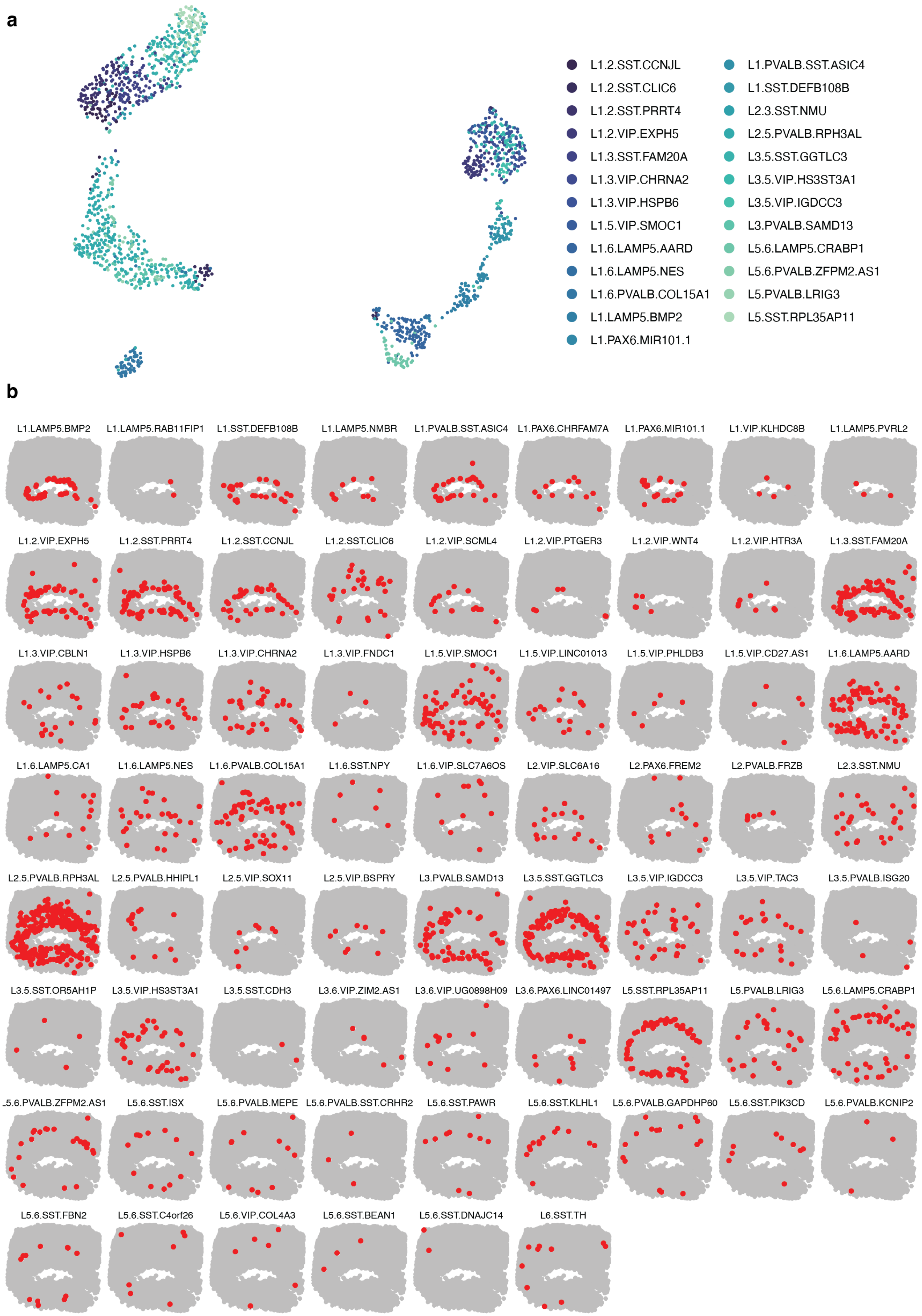

Supplementary Figure. 3. Slide-tags snRNA-seq in the human brain enables mapping of inhibitory neuron sub-cell-types. a, UMAP and label transfer of principal inhibitory subtypes. **b,** Inhibitory neuron subtypes plotted by spatial location. Subtype names from Bakken et al., 2021.

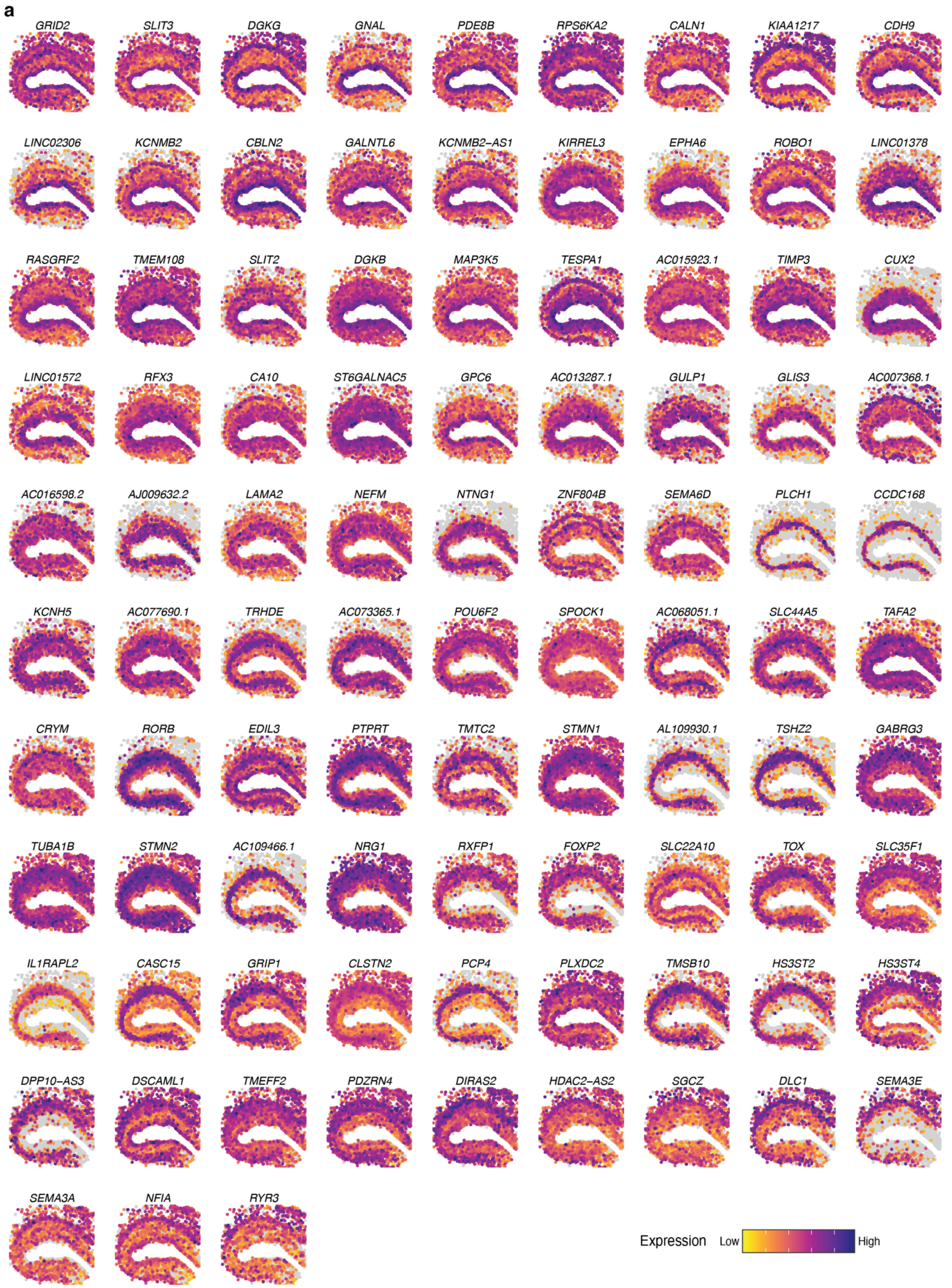

Supplementary Figure. 4. Slide-tags snRNA-seq in the human brain reveals cell-type specific spatial gradients of gene expression in excitatory neurons. Spatially varying genes identified in: **a,** Excitatory neurons

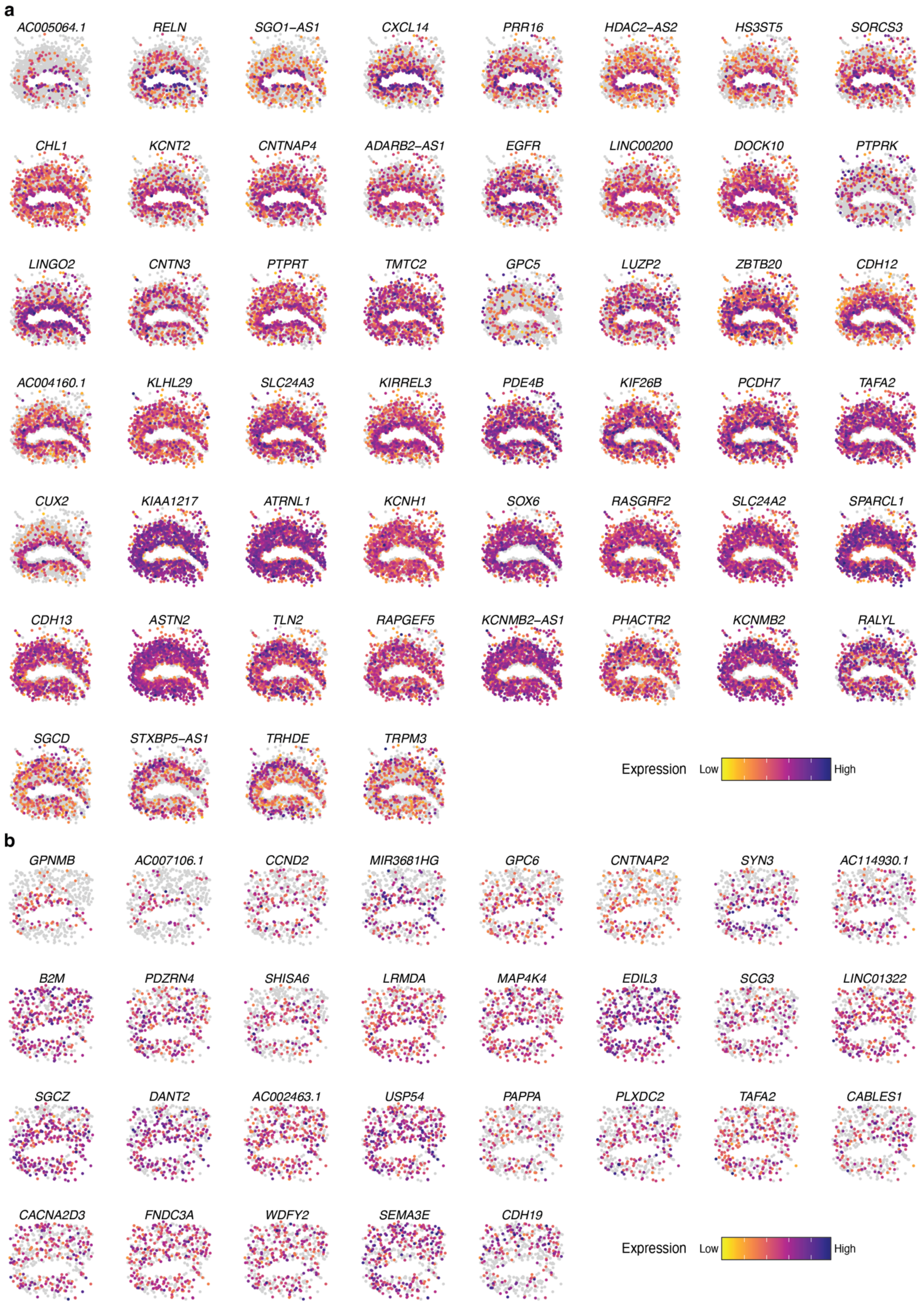

Supplementary Figure. 5. Slide-tags snRNA-seq in the human brain reveals cell-type specific spatial gradients of gene expression in inhibitory neurons and OPCs. Spatially varying genes identified in: **a,** Inhibitory neurons, **b,** Oligodendrocyte Precursor Cells (OPCs)

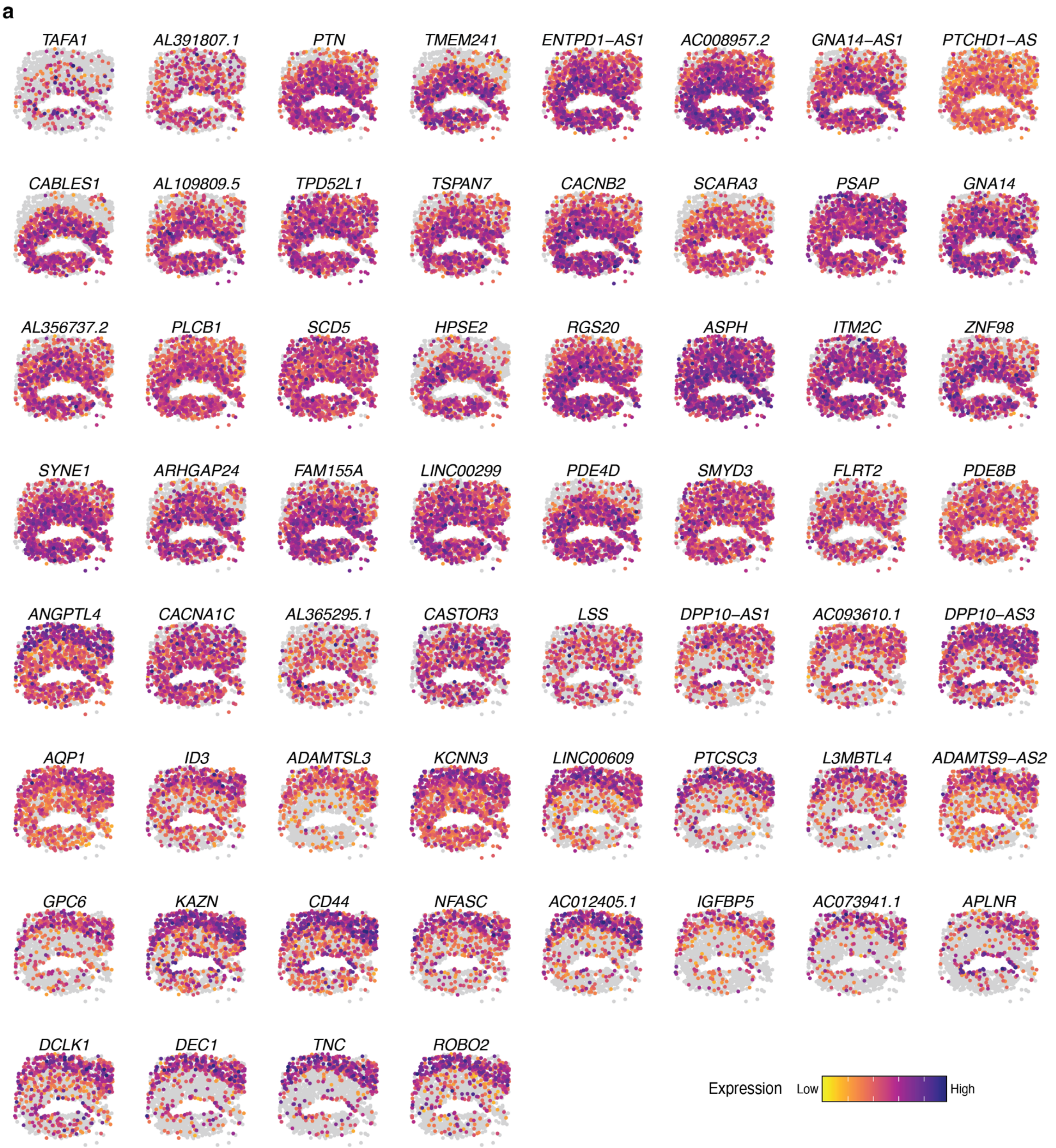

Supplementary Figure. 6. Slide-tags snRNA-seq in the human brain reveals cell-type specific spatial gradients of gene expression in astrocytes. Spatially varying genes identified in: **a,** Astrocytes.

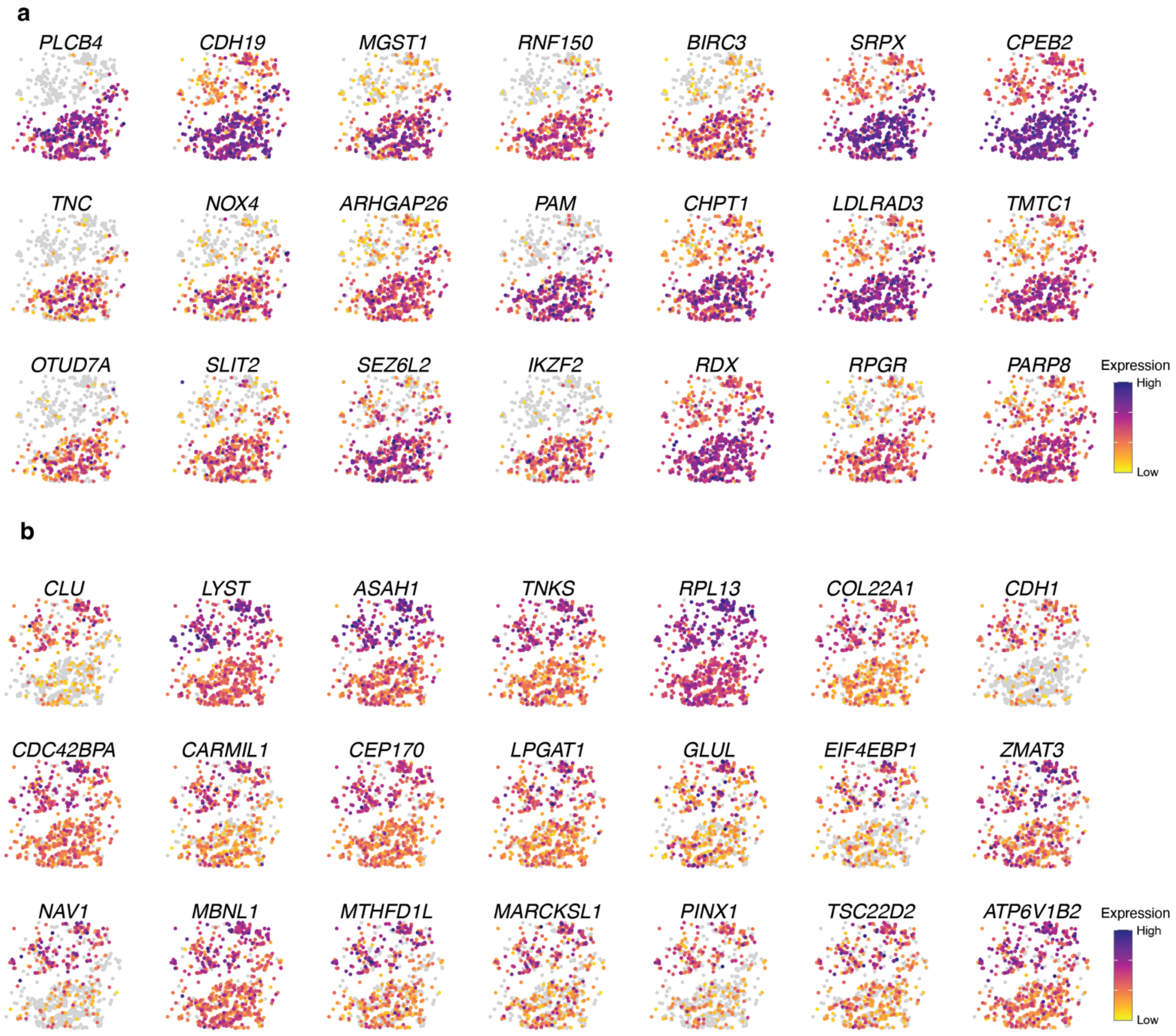

Supplementary Figure. 7. Spatial distribution of tumour 1 and tumour 2 marker genes in Slide-tags multiome. Gene expression plots for the top 21 genes significantly upregulated in tumour 1 (**a**) and tumour 2 (**b**) by both gene expression and chromatin genescore, ranked by gene expression $\log_2$FC.

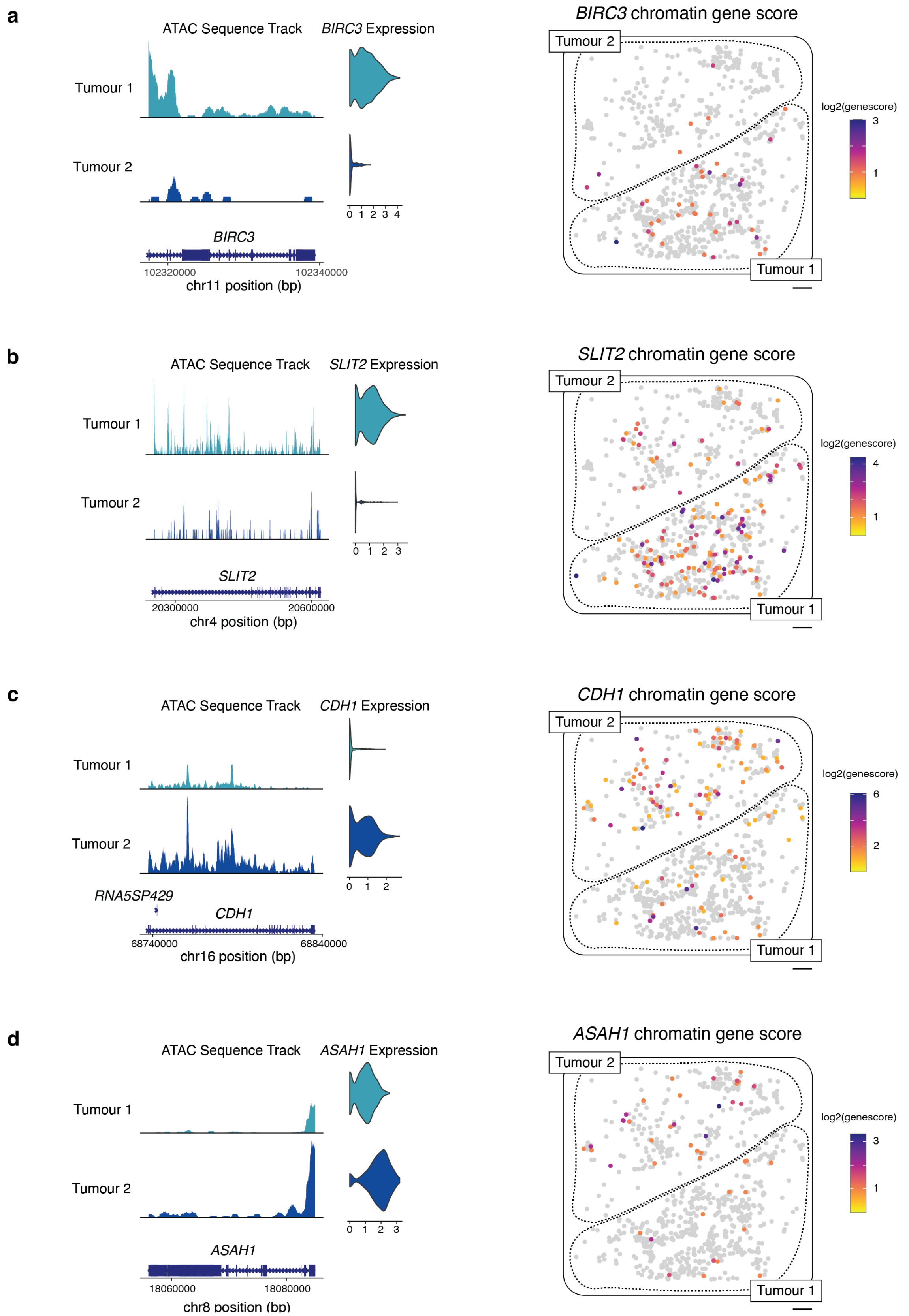

Supplementary Figure. 8. Mesenchymal-like and melanocytic-like cell state marker genes different between tumour 1 and tumour 2. a, *BIRC3* and **b,** *SLIT2* markers of mesenchymal-like cell state upregulated in tumour 1. **c,** CDH1 and **d,** ASAH1 markers of melanocytic-like cell state upregulated in tumour 2. Plots show ATAC sequence track, gene expression violin plot, and chromatin gene scores plotted in space.
